# Soil biota abundance supports ecosystem multifunctionality under carbon farming

**DOI:** 10.1093/nsr/nwag247

**Published:** 2026-04-29

**Authors:** Wenfeng Xue, Ting Liu, Saisai Cheng, Xiaoyun Chen, Joann K Whalen, Manqiang Liu

**Affiliations:** Centre for Grassland Microbiome, State Key Laboratory of Herbage Improvement and Grassland Agro-Ecosystems, College of Pastoral Agriculture Science and Technology, Lanzhou University, Lanzhou 730000, China; Soil Ecology Lab, College of Resources and Environmental Sciences, Nanjing Agricultural University, Nanjing 210095, China; Soil Ecology Lab, College of Resources and Environmental Sciences, Nanjing Agricultural University, Nanjing 210095, China; Centre for Grassland Microbiome, State Key Laboratory of Herbage Improvement and Grassland Agro-Ecosystems, College of Pastoral Agriculture Science and Technology, Lanzhou University, Lanzhou 730000, China; Soil Ecology Lab, College of Resources and Environmental Sciences, Nanjing Agricultural University, Nanjing 210095, China; Department of Natural Resource Sciences, McGill University, Montreal, Quebec H9X 3V9, Canada; Chair of Soil Science, Mohammed VI Polytechnic University, Ben Guerir 43150, Morocco; Centre for Grassland Microbiome, State Key Laboratory of Herbage Improvement and Grassland Agro-Ecosystems, College of Pastoral Agriculture Science and Technology, Lanzhou University, Lanzhou 730000, China; Soil Ecology Lab, College of Resources and Environmental Sciences, Nanjing Agricultural University, Nanjing 210095, China

**Keywords:** biodiversity–ecosystem functioning relationships, organic carbon inputs, cover crops, vermicompost, trade-offs

## Abstract

Organic substrates from plants and animals are crucial for sustainable agriculture, yet their effects on agroecosystem multifunctionality remain underexplored at the global scale. Using a meta-analysis of 8509 field observations, we examined how plant-based carbon inputs (legume cover crops) and animal-based carbon inputs (vermicompost) influence nine ecological functions. Both carbon inputs significantly improved agroecosystem multifunctionality, with legume cover crops increasing it by 17% and vermicompost by 31%. These improvements were concentrated in supporting and provisioning services. Soil biota abundance was strongly linked to multifunctionality and showed more frequent win–win relationships with other functions than species richness. Across agroecosystems, soil biota abundance was primarily associated with local soil properties, followed by climate factors. Our findings highlight soil biota abundance as a community attribute complementary to species richness that contributes to agroecosystem multifunctionality. We further propose site-specific management strategies that align plant- and animal-based carbon inputs with local environmental conditions in global croplands to enhance multifunctionality.

## Teaser

Global field evidence shows that legume cover crops and vermicompost strengthen agroecosystem multifunctionality by 17%–31%, with soil biota abundance emerging as a key driver of these benefits and fostering win–win relationships among multiple ecosystem functions.

## INTRODUCTION

Global food demand has intensified environmental challenges, including soil degradation, carbon decline, biodiversity loss, and greenhouse gas emissions [1,2]. Carbon farming is emerging as a sustainable agricultural practice that improves soil health by replenishing soil carbon and supporting soil biota [3,4]. This approach relies on organic inputs from either plant-based carbon sources, such as cover crops, or animal-based carbon sources, such as organic fertilizers [5–7]. Beyond their potential to increase carbon storage, carbon farming can support multiple ecosystem functions, referred to here as multifunctionality, that are essential for ecosystem services and human welfare. Understanding how these inputs influence multifunctionality is therefore important for optimizing sustainable and productive agroecosystems.

Soil biodiversity contributes to ecosystem multifunctionality by regulating nutrient cycling, carbon sequestration, and primary production through multiple soil biota-mediated pathways [8–10]. In natural ecosystems, strong aboveground–belowground linkages often reinforce biodiversity–ecosystem functioning relationships, which are commonly interpreted through changes in species richness [11–13]. However, these relationships can weaken at broader spatial scales, where increasing environmental heterogeneity, species turnover and shifts in community dominance may weaken the coupling between richness and ecosystem functioning [14–17]. This may be particularly relevant in agroecosystems, where monoculture, simplified resource inputs and frequent disturbance can strengthen dominance effects. Species richness records species presence, whereas abundance provides additional information on variation in community structure and dominance [18]. This distinction becomes especially important in communities with highly uneven, long-tailed abundance distributions, where a few taxa dominate and many remain rare [19,20]. In such communities, the traits of dominant taxa can disproportionately influence ecosystem processes through mass-ratio effects [21–23]. Carbon inputs may further strengthen these patterns by increasing soil biota abundance [24–26]. Moreover, abundance often responds more rapidly than richness to environmental change, as shifts in population size can precede species turnover [18,27]. It also reflects the active biomass pool and metabolic potential underlying decomposition, mineralization, and carbon stabilization [28]. We therefore hypothesize that, among complementary dimensions of soil biodiversity, soil biota abundance is more strongly linked to multifunctionality than species richness under carbon farming.

Carbon farming utilizes both plant- and animal-based sources, ranging from diverse cover crops and straw residues to livestock manure and composts. Legume cover crops often improve soil health more than non-legume cover crops by increasing nitrogen availability through biological nitrogen fixation [29–31], whereas vermicompost can outperform traditional compost because of its superior biological, nutritional, and physicochemical properties [32,33]. Yet, the effects of plant- and animal-based carbon inputs on soil biota and associated ecosystem functions vary widely across agroecosystems. This variation probably arises from interactions among climate, soil properties, and substrate characteristics. Temperature and precipitation regulate substrate decomposition and the energy supply available to soil biota [34,35], where soil pH, texture and nutrient status shape resource availability and the physicochemical habitat in which soil biota operate [36]. The nutrient stoichiometry and application rate of these carbon inputs may further modify their value as carbon and nutrient sources for soil biota [37,38]. We therefore hypothesize that the effects of plant- and animal-based carbon inputs on agroecosystem multifunctionality depend on how different inputs regulate soil biota under specific environmental conditions.

To test these hypotheses, we built a global dataset of 8509 observations on legume cover crops and vermicompost and quantified nine agroecosystem functions: soil biota richness, soil biota abundance, soil activity, soil fertility, soil physical structure, water regulation, climate regulation, plant productivity, and product quality (Fig. S1). These functions capture key dimensions of agroecosystem services related to biodiversity conservation, soil health preservation, pollution control, and food production [39–41]. Soil biota were represented by four major groups, bacteria, fungi, nematodes, and earthworms. These groups encompass major microbial and faunal compartments of soil food webs across key trophic and functional levels and are among the most frequently reported soil biota across studies [10,42], making them suitable for consistent comparison in a global meta-analysis. Soil biota abundance and richness were treated as functional components because they reflect the magnitude and structure of biological communities underlying ecosystem processes [39,43,44]. A total of 53 measurable variables were selected to characterize the nine functions (Table S1). We first quantified the relative contributions of soil biota abundance and richness to agroecosystem multifunctionality, and then used boosted regression tree (BRT) and mixed-effect meta-regression analyses to identify how climate, initial soil properties and substrate characteristics shaped soil biota abundance under each input type. Finally, we predicted the global benefits of soil biota abundance from various carbon inputs and derived site-specific recommendations for maximizing multifunctionality in global croplands.

## RESULTS

### Carbon farming supports multiple agroecosystem functions and services

**Figure 1.**
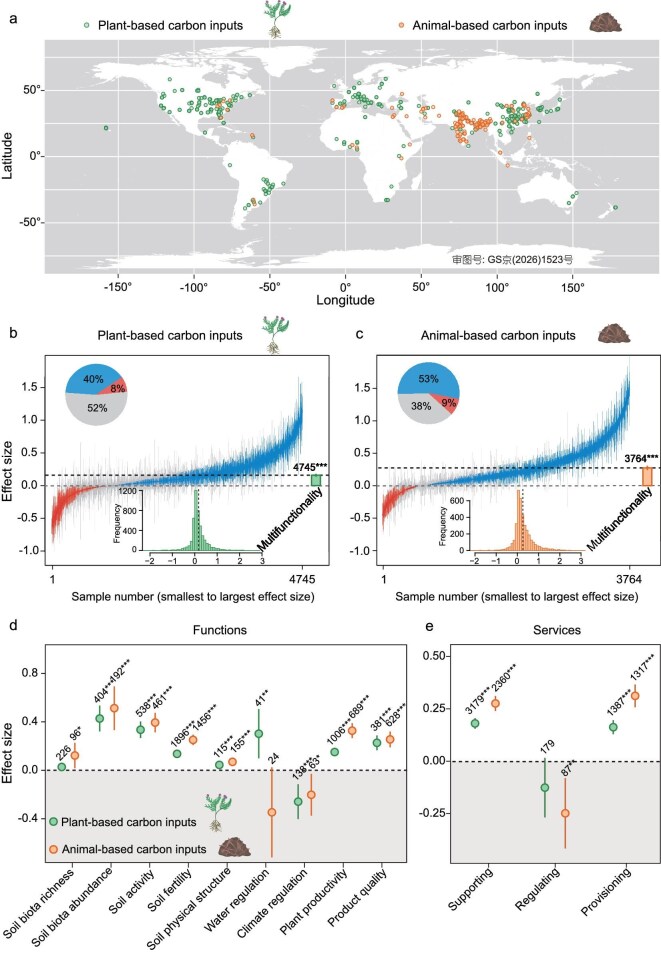
Global meta-analysis of agroecosystem multifunctionality in response to carbon farming. (a) Global distribution of 475 studies on plant-based carbon inputs (legume cover crops) and 198 studies on animal-based carbon inputs (vermicompost) in agroecosystems. (b and c) Effect sizes of the two carbon inputs on agroecosystem multifunctionality (calculated from 53 variables), shown as mean effect sizes with 95% CIs. Pie charts (upper left inset) show the number of samples with effect sizes greater than zero (blue, positive effect), less than zero (red, negative effect), and not significantly different from zero (gray). Histograms (lower right inset) show the frequency distribution of sample effect sizes. (d and e) Changes in agroecosystem functions (d) and agroecosystem services (e) in response to the two carbon inputs, based on mean effect sizes with 95% CIs. Numbers indicate sample sizes. Mean effect sizes were considered significant when the 95% CIs did not overlap zero (*P < 0.05, **P < 0.01, and ***P < 0.001).

We conducted a global meta-analysis to evaluate how plant- and animal-based carbon inputs, represented by legume cover crops and vermicompost, influence agroecosystem functions and services. Legume cover crops were widely used across Asia, Europe, and North America, whereas vermicompost was concentrated mainly in Asia, particularly in China and India (Fig. 1a). Both carbon inputs were frequently associated with improvements in soil fertility, such as soil organic carbon (C), and plant productivity, such as crop yield (Fig. S2).

Across the two carbon inputs, 78% of assessed ecosystem functions were positively affected (Fig. 1d). Overall multifunctionality increased by 17% under legumes and by 31% under vermicompost (*P* < 0.001; Fig. 1b and c, Tables S2 and S3). Soil biota abundance increased by 53%–67% under both carbon types (*P* < 0.001), whereas effects on soil biota richness were weaker: vermicompost increased richness by 13% (*P* < 0.05), whereas legumes had no significant effect (Fig. 1d, Fig. S3). These carbon inputs also promoted soil activity, soil fertility, soil physical structure, plant productivity, and product quality by 5%–48%, but reduced climate regulation (*P* < 0.05; Fig. 1d). At the ecosystem-service level, both carbon inputs increased supporting services by 20%–32% and provisioning services by 18%–37% (*P* < 0.001), whereas vermicompost reduced regulating services by 22% (*P* < 0.01; Fig. 1e, Tables S2 and S3).

### Soil biota abundance is the main predictor of multifunctionality under carbon farming

**Figure 2.**
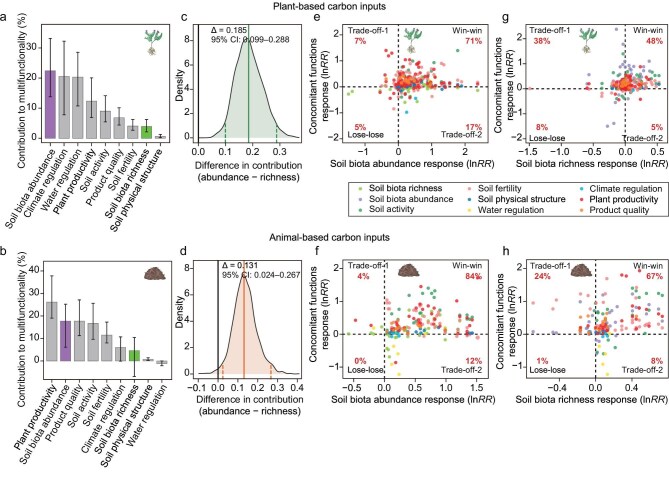
Soil biota abundance contributes more strongly than species richness to agroecosystem multifunctionality. (a and b) Relative contributions of individual functions to multifunctionality under plant-based (a) and animal-based (b) carbon inputs, estimated using covariance-based decomposition. Bars show mean contribution shares (%) and error bars indicate 95% bootstrap CIs (*n* = 2000). (c and d) Bootstrap distributions of the difference in contribution share between soil biota abundance and richness (Δ = *S*_abundance_ − *S*_richness_) under plant-based (c) and animal-based (d) carbon inputs. Solid lines indicate mean values, and dashed lines indicate 95% CIs. Positive Δ values indicate a greater contribution of abundance than richness. (e*–*h) Relationships between soil biota abundance or richness (*x*-axis) and concomitant ecosystem functions (*y*-axis), showing trade-off, lose–lose, and win–win patterns under plant-based (321 and 310 pairs of effect sizes, (e, g)) and animal-based (183 and 168 pairs of effect sizes, (f, h)) organic carbon inputs. Points represent paired ln*RR* values, each constructed by matching one effect size of soil biota abundance or richness with the effect size of a single ecosystem function within the same study. Values in each quadrant indicate the proportion of all paired effect-size observations in that panel. When multiple variables were measured for the same function, their mean effect size was used.

We next used a covariance-based decomposition approach to test whether soil biota abundance or richness contributed more strongly to multifunctionality. Under both carbon inputs, soil biota abundance accounted for a much larger share of the total covariance with multifunctionality than species richness (legumes: 22.4% vs. 3.9%; vermicompost: 17.8% vs. 4.6%; Fig. 2a and b). Under plant-based carbon inputs, the difference in contribution share between abundance and richness (Δ = *S*_abundance_  *− S*_richness_) was positive and stable (Δ = 0.185, 95% CI: 0.099–0.288; Fig. 2c), indicating an 18.5% greater contribution of abundance to multifunctionality. Under animal-based carbon inputs, this difference remained positive (Δ = 0.131, 95% CI: 0.024–0.267; Fig. 2d), corresponding to a 13.1% greater contribution of abundance.

We also compared trade-offs, lose–lose, and win–win relationships between soil biota abundance or richness and other ecosystem functions using paired effect sizes. Under both carbon inputs, win–win scenarios predominated, but they were more frequent for soil biota abundance, which showed win–win outcomes with 71%–84% of other ecosystem functions (Fig. 2e and f). In contrast, soil biota richness showed win–win outcomes with 48%–67% of functions and more trade-off and lose–lose relationships than abundance (Fig. 2g and h, Table S4).

### Soil biota abundance is strongly associated with local soil properties and climate

**Figure 3.**
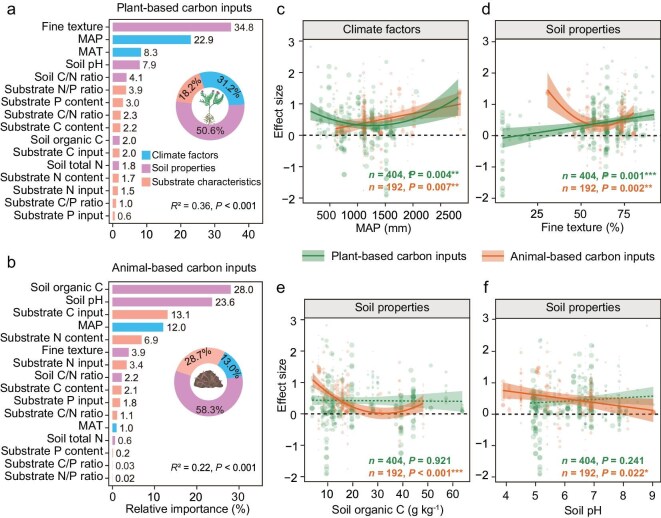
Soil biota abundance under carbon farming is mainly predicted by local soil properties and climate. (a and b) Relative importance of environmental variables affecting soil biota abundance under plant-based (a) and animal-based (b) carbon inputs, estimated using BRT analysis. Insets show the relative importance of climate factors, local soil properties, and substrate characteristics. Relationship between model-predicted and observed effects on soil biota abundance are shown in Fig. S4. (c–f) Relationships of soil biota abundance with climate factors and local soil properties, estimated using mixed-effects meta-regression. The best-fitting linear or quadratic model was selected using likelihood ratio tests. Point size is proportional to the inverse of the sampling variance of the response ratio. Significant trends (*P* < 0.05) are shown as solid bold lines with 95% CIs, while non-significant trends are shown as dashed lines. All regression models and fitted equations are provided in Tables S9 and S10. Abbreviations: MAT, mean annual temperature; MAP, mean annual precipitation; fine texture, percentage of clay plus silt.

Although carbon farming generally increased soil biota abundance, responses varied markedly across the meta-analysis dataset. We analyzed 596 observations (404 from legume cover crops and 192 from vermicompost) to assess how climate, initial soil properties and substrate factors influenced soil biota abundance (Table S5). The BRT analysis identified 16 predictors that together explained 22%–36% of the variation in soil biota abundance (Fig. 3, Fig. S4).

Under legume-based carbon inputs, initial soil properties were the dominant predictors of soil biota abundance, accounting for 51% of total predictor importance, whereas climate factors accounted for 31%. Fine texture (clay plus silt) and mean annual precipitation (MAP) were the strongest predictors (Fig. 3a). Soil biota abundance increased with fine texture, whereas its relationship with MAP was nonlinear, declining initially and then increasing at higher precipitation levels (*P* < 0.01; Fig. 3c and d).

Under vermicompost-based carbon inputs, initial soil properties were likewise the dominant predictors, accounting for 58% of total predictor importance (Fig. 3b). Soil organic C and soil pH were the strongest predictors, and both were negatively associated with soil biota abundance responses (*P* < 0.05; Fig. 3e and f). MAP was also strongly associated with soil biota abundance responses (*P* < 0.01; Fig. 3c), but its positive effects were weaker under lower precipitation conditions.

### Optimizing the benefits of carbon farming under site-specific conditions

**Figure 4.**
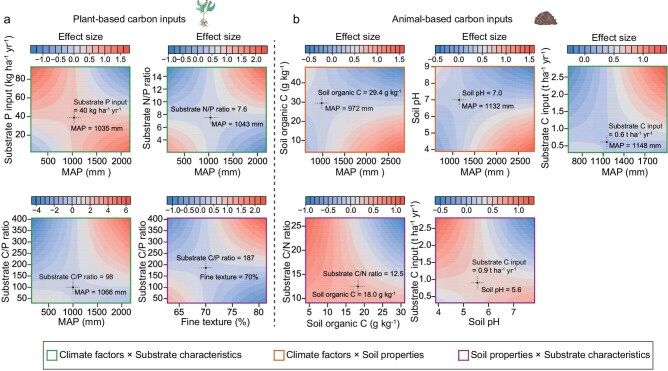
Soil biota abundance responses vary with interactions among local soil properties, climate factors, and substrate characteristics. The vertical and horizontal axes show the two interacting factors. Shaded surfaces indicate the effect sizes of plant-based (a) and animal-based (b) carbon inputs on soil biota abundance, with solid dots indicating threshold points where the effect of one factor changes along the gradient of the other. For example, in the top-left panel, MAP modifies the relationship between substrate P input and soil biota abundance, with the shift occurring at MAP = 1035 mm: abundance increases with P input under drier conditions but weakens or reverses under wetter conditions. All possible interactions are provided in Tables S6 and S7.

**Figure 5.**
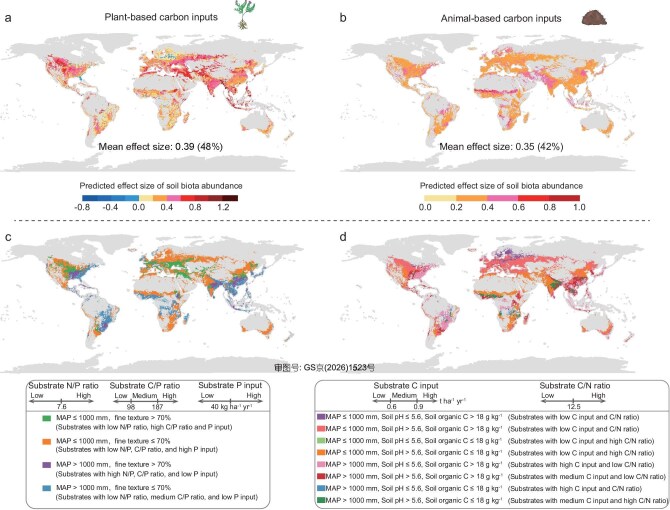
Predicted benefits of carbon farming for soil biota abundance and site-specific recommendations for maximizing multifunctionality in global croplands. (a and b) Predicted benefits of plant-based (a) and animal-based (b) carbon inputs for soil biota abundance. Mean values indicate mean effect sizes, with percentage changes provided in brackets. (c and d) Site-specific recommendations for optimizing two carbon inputs according to local environmental conditions. Colors indicate regions sharing similar environmental constraints and therefore similar recommended input characteristics (shown in parentheses in the legend). For example, regions with MAP <1000 mm and fine texture >70% are predicted to support higher soil biota abundance and multifunctionality under plant-based carbon inputs with low N/P (<7.6), high C/P (>187), and high P input (>40 kg ha^−1^ yr^−1^) (c). Similarly, regions with MAP <1000 mm, soil pH <5.6 and soil organic C >18 g kg^−1^ are predicted to benefit from vermicompost with a low C/N (12.5) and low C input (<0.6 t ha^−1^ yr^−1^) (d). Predictions were generated using a BRT model integrating climate and soil variables (MAT, MAP, fine texture, soil organic C, soil total N, soil C/N, and soil pH). Global cropland, climate, and soil data were mapped at a 10-km resolution.

Climate, local soil properties, and substrate characteristics interacted strongly in influencing soil biota abundance (Fig. 4, Tables S6 and S7). For legume cover crops, substrate characteristics (N/P ratio, C/P ratio, and P input) interacted with MAP and fine texture. High N/P (>7.6) and C/P (>187) ratios maximized abundance gains in warm, humid regions with fine-textured soils (>70%), whereas under drier conditions (MAP <1000 mm) and coarser soils, lower N/P and C/P ratios were more beneficial when P input exceeded 40 kg ha^−1^ yr^−1^ (Figs 4a and 5a, Fig. S5a).

For vermicompost, lower C/N ratios (<12.5) increased soil biota abundance most strongly in soils with high soil organic C (>18 g kg^−1^), yielding 62% greater abundance than under higher C/N substrates (Fig. 4b, Table S8). The effects of C input further depended on MAP and soil pH: higher C input was more beneficial under wetter conditions (MAP >1000 mm), whereas lower C input (<0.9 t ha^−1^ yr^−1^) was more beneficial under drier conditions or when soil pH exceeded 5.6 (Fig. 4b).

We next applied a global gridded model to predict spatial patterns of soil biota abundance responses to plant- and animal-based carbon inputs and their implications for agroecosystem multifunctionality (Fig. 5). Legumes and vermicompost increased soil biota abundance by 48% and 42%, respectively (Fig. 5a and b). Predicted benefits varied across regions. Plant-based carbon inputs were generally less effective than animal-based carbon inputs in Northern Europe, Eastern North America, and much of Africa and South America, particularly where fine texture was below 70%, soil pH was below 5.6, or soil organic C was high. In contrast, plant-based carbon inputs often outperformed animal-based inputs across large parts of Asia, Eastern Europe, and Southwestern North America, in many cases increasing soil biota abundance by >80% (mean effect size >0.60; Fig. 5a and b).

Based on these model predictions, we derived site-specific recommendations to optimize soil biota benefits and enhance agroecosystem multifunctionality (Fig. S6). In semi-arid regions with MAP <1000 mm and fine texture >70%, plant-based carbon inputs with low N/P (<7.5), high C/P (>180), and high P input (>40 kg ha^−1^ yr^−1^) were predicted to maximize soil biota abundance (Fig. 5c). In semi-arid regions with soil pH >5.6 and high organic C (>18 g kg^−1^), animal-based carbon inputs with low C/N (<12.5) and low C input (<0.6 t ha^−1^ yr^−1^) were predicted to be most effective (Fig. 5d). These results show that matching organic carbon inputs to local soil and climate conditions can maximize soil biota abundance and multifunctionality gains across croplands.

## DISCUSSION

Extensive research over the past two decades has established aboveground and belowground biodiversity as key drivers of ecosystem functioning [45–47]. However, how belowground biodiversity shapes ecosystem multifunctionality in managed agroecosystems remains poorly understood. Our study provides a global quantitative assessment of how carbon farming influences agroecosystem multifunctionality, showing that multifunctionality gains were more strongly associated with increases in soil biota abundance than with changes in species richness. We further identify local soil properties, followed by climate factors, as the main predictors of soil biota abundance, helping to clarify the environmental context in which biodiversity–ecosystem functioning relationships are expressed in agroecosystems.

### Soil biota abundance plays a major role in agroecosystem multifunctionality

Our study shows that soil biota abundance plays a stronger role than species richness in promoting agroecosystem multifunctionality under carbon farming practices. This finding supports our hypothesis and aligns with the well-established concept of biodiversity–ecosystem functioning relationships. One possible explanation is that abundance, by reflecting biomass and population size, provides a more sensitive and ecologically meaningful indicator of community responses to anthropogenic disturbances than richness alone [23,27,48]. In croplands, where soil biota are often constrained by limited organic carbon and energy, initial responses to organic inputs are likely increases in the abundance of existing populations as resource limitation is alleviated [49]. Changes in community composition and diversity are more likely to emerge later, as inputs accumulate and differences in substrate type and quality begin to shape community assembly [50–52]. Abundance therefore captures early changes in soil communities that are more tightly linked to ecosystem functioning [25].

A second explanation is that abundance may better capture changes in dominant taxa, whose traits disproportionately influence ecosystem functioning [53]. This interpretation is consistent with the mass-ratio hypothesis [22], which emphasizes the functional importance of dominant taxa and their traits. Accordingly, soil biota abundance becomes particularly informative when increases in abundance reflect shifts in community dominance toward functionally important taxa [23,27]. In contrast, the loss of rare species may have more limited immediate effects on net community functioning when their roles are weak, marginal, or functionally redundant [54]. Consistent with this, variation in ecosystem functioning is often driven more by changes in dominant taxa abundance than by species presence or absence alone [21,23,55].

A third explanation is that functional redundancy may weaken or saturate the relationship between richness and multifunctionality, contributing to nonlinear biodiversity–ecosystem functioning relationships [56]. Once key functional groups are present, adding more species may provide only limited additional functional gains. In contrast, increasing the abundance of organisms within these groups can increase biomass pools, intensify trophic interactions, and strengthen energy transfer across soil food webs [10,57,58], thereby improving resource-use efficiency and coupling multiple ecosystem processes, even under high functional redundancy [59,60]. Under these conditions, abundance is more likely than richness to translate into measurable gains in multifunctionality.

### Local soil properties and climate are strongly associated with soil biota abundance

Local soil properties were the main regulators of soil biota abundance in both plant- and animal-based carbon farming systems, although the key predictors differed between the two input types. Under plant-based carbon inputs, fine texture was the strongest positive predictor, likely because it improves water and nutrient retention and thereby supports soil biota activity and plant–soil interactions [61,62]. These conditions likely favor root exudation and biological nitrogen fixation during growth [63], and also promote residue decomposition after senescence by maintaining more stable moisture and nutrient conditions [64].

In contrast, under animal-based carbon inputs, soil organic C and pH were the dominant predictors, indicating that vermicompost effects depend more strongly on soil chemical conditions. Vermicompost was most effective in soils with low initial organic C and high acidity, where its labile organic matter and bioavailable nutrients likely alleviate carbon and nutrient limitation [65–67]. In acidic soils, its alkaline properties may further enhance soil biota abundance by neutralizing soil pH [66]. In soils with higher organic C, vermicompost responses depended more strongly on input stoichiometry, with lower C/N ratios being more beneficial [68]. In soils with higher pH, moderate total C input was also more favorable, indicating that vermicompost effects depend on how added carbon interacts with background soil conditions.

In addition to soil properties, MAP was another key predictor of soil biota abundance in carbon farming systems, mainly through interactions with substrate characteristics. Precipitation likely determines how efficiently substrate carbon and nutrients are converted into biomass. Under wetter conditions, organic carbon inputs are decomposed more efficiently, increasing resource availability and population growth [69]. This may explain why legumes with high N/P and C/P ratios were more beneficial in humid regions, where nutrient mineralization is likely to be enhanced. In contrast, in drier regions, where decomposition is limited by moisture, phosphorus availability appears to become more important, and legumes with low N/P and C/P ratios may be more beneficial because they promote P solubilization through root exudates [70–72]. A similar pattern was observed for vermicompost: the optimal level of carbon input shifted with precipitation, favoring lower inputs in drier regions and higher inputs under wetter conditions, probably because moisture availability determines how efficiently added carbon can be biologically processed [32,73].

### Site-specific recommendations for optimizing carbon farming in global croplands

The effectiveness of carbon farming in enhancing soil biota abundance varies strongly with climate and local soil conditions, indicating that management should be tailored to the regional context. In regions with coarse-textured soils, high soil organic C, and low pH, such as in parts of Northern Europe, Eastern North America, and South America, plant-based carbon inputs are generally less effective. Under these conditions, slow decomposition and limited nutrient mineralization are likely to constrain soil biota responses [74,75]. In such regions, legume inputs with low N/P ratios (<7.6) or a greater reliance on vermicompost may help alleviate these constraints by providing more bioavailable nutrients and partially buffering soil acidity, thereby enhancing soil biota abundance and multifunctionality. In contrast, in semi-arid regions with relatively high soil organic C, particularly across parts of Southwestern North America, Eastern Europe, and Northern Asia, moisture limitation appears to constrain microbial activity and the decomposition of plant-derived carbon [76]. Plant-based carbon inputs alone may therefore be constrained under these conditions. Combining them with vermicompost characterized by a low C/N ratio, while limiting total C input, may better support soil biota abundance by supplying more readily available nutrients.

## CONCLUSION

Our study provides a global-scale quantitative assessment of how organic carbon inputs enhance ecosystem multifunctionality, demonstrating that in agroecosystems, soil biota abundance contributes more strongly to multifunctionality gains than species richness. This does not mean that species richness is unimportant. Rather, its effects in agroecosystems may be more context dependent, indirect, or expressed over longer time scales than could be captured here. Our results therefore suggest that abundance and richness represent complementary dimensions of soil biodiversity, and extend biodiversity–ecosystem functioning theory developed largely in natural ecosystems to managed agroecosystems, where intensive management and large organic inputs may make multifunctionality more directly linked to abundance.

By identifying local soil properties, followed by climate factors, as the dominant determinants of soil biota responses, our study provides a data-driven basis for optimizing organic carbon inputs under site-specific conditions. The multifunctionality gains associated with organic carbon inputs were closely linked to soil biota abundance, which often showed win–win relationships with other ecosystem functions. Our results further suggest that plant- and animal-based carbon inputs should be matched to local soil and climate conditions rather than applied uniformly across agroecosystems. Such site-specific carbon-farming management can strengthen multiple ecological functions simultaneously, support more resilient agroecosystems and more stable crop production, and provide an ecological basis for agricultural management to meet broader One Health goals.

The limitations of this study include the focus of our synthesis on legume cover crops and vermicompost, without considering other organic amendments such as straw, crop residues, biochar, and manure. Therefore, the observed patterns may not extend directly to these organic amendments. In addition, as a global meta-analysis, our study identifies broad statistical relationships but cannot fully resolve the causal pathways and temporal dynamics linking organic carbon inputs, soil biota, and agroecosystem multifunctionality. Future studies should therefore combine broader comparisons of organic amendments with long-term experiments to clarify the causal pathways linking soil biota to agroecosystem multifunctionality.

## METHODS

### Meta-analysis dataset construction

To evaluate the global ecological performance of legume cover crops and vermicompost as plant- and animal-based organic carbon inputs, we searched Web of Science and Google Scholar from 1 January 2000 to 30 April 2024. Study screening followed Preferred Reporting Items for Systematic Reviews and Meta-Analyses (PRISMA) guidelines [77] (Fig. S7). We retained field studies with paired control and treatment groups, at least three replicates per group, and at least one ecosystem-function variable measured under the same conditions in control and treatment plots. For legume cover crops, studies had to include a no-cover-crop control and at least one identified legume cover crop treatment. For vermicompost, studies had to include at least one vermicompost treatment and a vermicompost-free control. To reduce confounding, treatment and control groups were compared only when they differed solely in the use of legume cover crops or vermicompost and were sampled simultaneously. Based on these criteria, we retained 475 studies for legume cover crops and 198 studies for vermicompost. Detailed search terms, screening steps, and inclusion criteria are provided in the Supplementary Methods.

### Data collection

We compiled 53 ecosystem variables representing nine agroecosystem functions: soil biota richness, soil biota abundance, soil activity, soil fertility, soil physical structure, water regulation, climate regulation, plant productivity, and product quality. These functions were further grouped into three ecosystem-service categories: supporting, regulating, and provisioning services (Fig. S1). Soil biota were represented by four major groups, bacteria, fungi, nematodes, and earthworms, which together capture key trophic and functional components of soil food webs. Soil biota abundance and richness were included as functional components because they reflect the size and structure of biological communities underlying ecosystem processes. For each study, we also recorded site location, climate variables, pre-experiment soil properties, and substrate characteristics (Table S5). Detailed variable definitions, measurement approaches, units, and data-extraction procedures are provided in Table S1 and the Supplementary Methods.

### Statistical analyses

To standardize comparisons across studies, we used the natural log-transformed response ratio (ln*RR*) as the effect-size metric [78], calculated from treatment and control means. For variables in which lower values indicate better ecosystem performance, effect sizes were sign-adjusted so that positive values consistently represented functional improvement. Mean effect sizes and 95% confidence intervals (CIs) were estimated using three-level hierarchical random-effects meta-analysis to account for dependence among multiple effect sizes reported within the same study [79,80]. Non-independence among effect sizes sharing a common control was further handled using variance–covariance structures [81]. ln*RR* values were also back-transformed to percentage change to facilitate interpretation of effect-size magnitude [78,82].

To compare the relative contributions of soil biota abundance and richness to multifunctionality, we first averaged multiple effect sizes for the same function within the same treatment-control comparison and then applied a covariance-based decomposition approach. This method quantified each function’s proportional share of the total covariance with the multifunctionality index, allowing direct comparison of the relative roles of soil biota abundance, soil biota richness, and other ecosystem functions while minimizing bias arising from structural overlap in index construction. We also assessed trade-off, lose–lose, and win–win relationships by pairing effect sizes of soil biota abundance or richness with those of other ecosystem functions.

To identify the determinants of soil biota abundance, we first used BRT analysis to rank the relative importance of climate variables, baseline soil properties, and substrate characteristics. We then used mixed-effects meta-regression to test linear, nonlinear, and pairwise interaction effects of these variables on soil biota abundance. For interaction models, response surfaces were visualized to show how the effect of one predictor changed along the gradient of another, and transition points were analytically derived from the fitted interaction models.

To extend our results to global croplands, we applied the fitted BRT models to gridded datasets of cropland, climate, and soil properties to predict the potential benefits of carbon farming for soil biota abundance worldwide. Detailed model specifications, equations, transition-point derivations, publication-bias tests, and robustness analyses are provided in the Supplementary Methods. All statistical analyses and figures were generated using R version 4.3.1 [83].

## Supplementary Material

nwag247_Supplemental_Files

## Data Availability

All the necessary data to validate the conclusions presented in the paper is available in the paper and/or the Supplementary Materials. The global cropland data for the year 2019 can be accessed at https://glad.umd.edu/dataset/croplands. Climate data were sourced from the WorldClim database (www.worldclim.org), while soil properties data—including clay content, silt content, soil organic C, total N and soil pH—were obtained from SoilGrids (http://www.isric.org/explore/soilgrids).

## References

[1] Wall DH, Nielsen UN, Six J. Soil biodiversity and human health. Nature 2015; 528: 69–76. 10.1038/nature15744 26595276

[2] Tilman D, Balzer C, Hill J et al. Global food demand and the sustainable intensification of agriculture. Proc Natl Acad Sci USA 2011; 108: 20260–4. 10.1073/pnas.1116437108 22106295 PMC3250154

[3] McDonald H, Frelih-Larsen A, Keenleyside C et al. Carbon farming, making agriculture fit for 2030. Study For the Committee on Environment, Public Health and Food Safety (ENVI), Policy Department For Economic, Scientific and Quality of Life Policies, European Parliament, Luxembourg. 2021 (PE 695.482).

[4] Thorsøe MH, Facq E, Criscuoli I et al. Carbon farming: the foundation for carbon farming schemes—lessons learned from 160 European schemes. Land Use Policy 2025; 158: 107747. 10.1016/j.landusepol.2025.107747

[5] Jansson C, Faiola C, Wingler A et al. Crops for carbon farming. Front Plant Sci 2021; 12: 636709. 10.3389/fpls.2021.636709 34149744 PMC8211891

[6] Gattinger A, Muller A, Haeni M et al. Enhanced top soil carbon stocks under organic farming. Proc Natl Acad Sci USA 2012; 109: 18226–31. 10.1073/pnas.1209429109 23071312 PMC3497757

[7] Bossio DA, Cook-Patton SC, Ellis PW et al. The role of soil carbon in natural climate solutions. Nat Sustain 2020; 3: 391–8. 10.1038/s41893-020-0491-z

[8] Wagg C, Bender SF, Widmer F et al. Soil biodiversity and soil community composition determine ecosystem multifunctionality. Proc Natl Acad Sci USA 2014; 111: 5266–70. 10.1073/pnas.1320054111 24639507 PMC3986181

[9] Wu D, Du E, Eisenhauer N et al. Global engineering effects of soil invertebrates on ecosystem functions. Nature 2025; 640: 120–9. 10.1038/s41586-025-08594-y 39939777

[10] Eisenhauer N, Sünnemann M, Pollierer MM et al. Soil biodiversity effects on ecosystems. Nat Rev Biodivers 2026; 2: 76–91. 10.1038/s44358-025-00123-z

[11] Zavaleta ES, Pasari JR, Hulvey KB et al. Sustaining multiple ecosystem functions in grassland communities requires higher biodiversity. Proc Natl Acad Sci USA 2010; 107: 1443–6. 10.1073/pnas.0906829107 20080690 PMC2824370

[12] Wang Y, Du J, Pang Z et al. Unimodal productivity-biodiversity relationship along the gradient of multidimensional resources across Chinese grasslands. Natl Sci Rev 2022; 9: nwac165. 10.1093/nsr/nwac165 36519072 PMC9743175

[13] Soliveres S, van der Plas F, Manning P et al. Biodiversity at multiple trophic levels is needed for ecosystem multifunctionality. Nature 2016; 536: 456–9. 10.1038/nature19092 27533038

[14] Thompson PL, Kefi S, Zelnik YR et al. Scaling up biodiversity–ecosystem functioning relationships: the role of environmental heterogeneity in space and time. Proc R Soc B: Biol Sci 2021; 288: 20202779. 10.1098/rspb.2020.2779 PMC7944106 33715425

[15] Mori AS, Isbell F, Seidl R. Beta-diversity, community assembly, and ecosystem functioning. Trends Ecol Evol 2018; 33: 549–64. 10.1016/j.tree.2018.04.012 29807839 PMC7612777

[16] Barnes AD, Weigelt P, Jochum M et al. Species richness and biomass explain spatial turnover in ecosystem functioning across tropical and temperate ecosystems. Phil Trans R Soc B 2016; 371: 20150279. 10.1098/rstb.2015.0279 27114580 PMC4843699

[17] van der Plas F, Manning P, Soliveres S et al. Biotic homogenization can decrease landscape-scale forest multifunctionality. Proc Natl Acad Sci USA 2016; 113: 3557–62. 10.1073/pnas.1517903113 26979952 PMC4822601

[18] Hordijk I, Maynard DS, Hart SP et al. Evenness mediates the global relationship between forest productivity and richness. J Ecol 2023; 111: 1308–26. 10.1111/1365-2745.14098

[19] Dangles O, Malmqvist B. Species richness-decomposition relationships depend on species dominance. Ecol Lett 2004; 7: 395–402. 10.1111/j.1461-0248.2004.00591.x

[20] McGill BJ, Etienne RS, Gray JS et al. Species abundance distributions: moving beyond single prediction theories to integration within an ecological framework. Ecol Lett 2007; 10: 995–1015. 10.1111/j.1461-0248.2007.01094.x 17845298

[21] Smith MD, Knapp AK. Dominant species maintain ecosystem function with non-random species loss. Ecol Lett 2003; 6: 509–17. 10.1046/j.1461-0248.2003.00454.x

[22] Grime JP . Benefits of plant diversity to ecosystems: immediate, filter and founder effects. J Ecol 1998; 86: 902–10. 10.1046/j.1365-2745.1998.00306.x

[23] Winfree R, Fox JW, Williams NM et al. Abundance of common species, not species richness, drives delivery of a real-world ecosystem service. Ecol Lett 2015; 18: 626–35. 10.1111/ele.12424 25959973

[24] Kim N, Zabaloy MC, Guan K et al. Do cover crops benefit soil microbiome? A meta-analysis of current research. Soil Biol Biochem 2020; 142: 107701. 10.1016/j.soilbio.2019.107701

[25] van Rijssel SQ, Koorneef GJ, Veen GFC et al. Conventional and organic farms with more intensive management have lower soil functionality. Science 2025; 388: 410–5. 10.1126/science.adr0211 40273235

[26] Fan K, Delgado-Baquerizo M, Guo X et al. Biodiversity of key-stone phylotypes determines crop production in a 4-decade fertilization experiment. ISME J 2021; 15: 550–61. 10.1038/s41396-020-00796-8 33028975 PMC8027226

[27] Hua F, Bruijnzeel LA, Meli P et al. The biodiversity and ecosystem service contributions and trade-offs of forest restoration approaches. Science 2022; 376: 839–44. 10.1126/science.abl4649 35298279

[28] Gaston KJ, Cox DTC, Canavelli SB et al. Population abundance and ecosystem service provision: the case of birds. Bioscience 2018; 68: 264–72. 10.1093/biosci/biy005 29686433 PMC5905662

[29] Vendig I, Guzman A, De La Cerda G et al. Quantifying direct yield benefits of soil carbon increases from cover cropping. Nat Sustain 2023; 6: 1125–34. 10.1038/s41893-023-01131-7

[30] Yao W, Yang Y, Beillouin D et al. Legume-rice rotations increase rice yields and carbon sequestration potential globally. One Earth 2025; 8: 101170. 10.1016/j.oneear.2024.12.006

[31] Lamichhane JR, Alletto L. Ecosystem services of cover crops: a research roadmap. Trends Plant Sci 2022; 27: 758–68. 10.1016/j.tplants.2022.03.014 35459600

[32] Lim SL, Wu TY, Lim PN et al. The use of vermicompost in organic farming: overview, effects on soil and economics. J Sci Food Agric 2015; 95: 1143–56. 10.1002/jsfa.6849 25130895

[33] Joshi R, Singh J, Vig AP. Vermicompost as an effective organic fertilizer and biocontrol agent: effect on growth, yield and quality of plants. Rev Environ Sci Biotechnol 2015; 14: 137–59. 10.1007/s11157-014-9347-1

[34] Outhwaite CL, McCann P, Newbold T. Agriculture and climate change are reshaping insect biodiversity worldwide. Nature 2022; 605: 97–102. 10.1038/s41586-022-04644-x 35444282

[35] Li Z, Wang F, Su F et al. Climate change drivers alter root controls over litter decomposition in a semi-arid grassland. Soil Biol Biochem 2021; 158: 108278. 10.1016/j.soilbio.2021.108278

[36] Hartmann M, Six J. Soil structure and microbiome functions in agroecosystems. Nat Rev Earth Environ 2022; 4: 4–18. 10.1038/s43017-022-00366-w

[37] Richard DB . Plant trait-based approaches for interrogating belowground function. Biol Environ Proc R Ir Acad 2017; 117B: 1–13. 10.3318/bioe.2017.03

[38] Bardgett RD, Mommer L, De Vries FT. Going underground: root traits as drivers of ecosystem processes. Trends Ecol Evol 2014; 29: 692–9. 10.1016/j.tree.2014.10.006 25459399

[39] Wittwer RA, Bender SF, Hartman K et al. Organic and conservation agriculture promote ecosystem multifunctionality. Sci Adv 2021; 7: eabg6995. 10.1126/sciadv.abg6995 34417179 PMC8378818

[40] Carpenter SR, Mooney HA, Agard J et al. Science for managing ecosystem services: beyond the millennium ecosystem assessment. Proc Natl Acad Sci USA 2009; 106: 1305–12. 10.1073/pnas.0808772106 19179280 PMC2635788

[41] Garland G, Banerjee S, Edlinger A et al. A closer look at the functions behind ecosystem multifunctionality: a review. J Ecol 2020; 109: 600–13. 10.1111/1365-2745.13511

[42] Potapov AM, Rozanova OL, Semenina EE et al. Size compartmentalization of energy channeling in terrestrial belowground food webs. Ecology 2021; 102: e03421. 10.1002/ecy.3421 34086977

[43] Liu S, Ward SE, Wilby A et al. Multiple targeted grassland restoration interventions enhance ecosystem service multifunctionality. Nat Commun 2025; 16: 3971. 10.1038/s41467-025-59157-8 40295479 PMC12037718

[44] Tamburini G, Bommarco R, Wanger TC et al. Agricultural diversification promotes multiple ecosystem services without compromising yield. Sci Adv 2020; 6: eaba1715. 10.1126/sciadv.aba1715 33148637 PMC7673676

[45] Loreau M, Naeem S, Inchausti P et al. Biodiversity and ecosystem functioning: current knowledge and future challenges. Science 2001; 294: 804–8. 10.1126/science.1064088 11679658

[46] Tilman D, Isbell F, Cowles JM. Biodiversity and ecosystem functioning. Annu Rev Ecol Evol Syst 2014; 45: 471–93. 10.1146/annurev-ecolsys-120213-091917

[47] Gamfeldt L, Roger F. Revisiting the biodiversity-ecosystem multifunctionality relationship. Nat Ecol Evol 2017; 1: 168. 10.1038/s41559-017-0168 28812584

[48] Saterberg T, Sellman S, Ebenman B. High frequency of functional extinctions in ecological networks. Nature 2013; 499: 468–70. 10.1038/nature12277 23831648

[49] Liu X, Han M, Zhang M et al. Fertilization managements mitigate microbial carbon and nitrogen limitations while preserving soil organic carbon in croplands compared to grasslands: a meta-analysis. J Environ Manage 2025; 395: 127927. 10.1016/j.jenvman.2025.127927 41205587

[50] Fu Y, Luo Y, Tang C et al. Succession of the soil bacterial community as resource utilization shifts from plant residues to rhizodeposits. Soil Biol Biochem 2022; 173: 108785. 10.1016/j.soilbio.2022.108785

[51] Maillard F, Colin Y, Viotti C et al. A cryptically diverse microbial community drives organic matter decomposition in forests. Appl Soil Ecol 2024; 193: 105148. 10.1016/j.apsoil.2023.105148

[52] Beidler KV, Phillips RP, Andrews E et al. Substrate quality drives fungal necromass decay and decomposer community structure under contrasting vegetation types. J Ecol 2020; 108: 1845–59. 10.1111/1365-2745.13385

[53] Gaston KJ . Common ecology. Bioscience 2011; 61: 354–62. 10.1525/bio.2011.61.5.4

[54] Bannar-Martin KH, Kremer CT, Ernest SKM et al. Integrating community assembly and biodiversity to better understand ecosystem function: the Community Assembly and the Functioning of Ecosystems (CAFE) approach. Ecol Lett 2018; 21: 167–80. 10.1111/ele.12895 29280282

[55] Lohbeck M, Bongers F, Martinez-Ramos M et al. The importance of biodiversity and dominance for multiple ecosystem functions in a human-modified tropical landscape. Ecology 2016; 97: 2772–9. 10.1002/ecy.1499 27859119

[56] Garcia FC, Bestion E, Warfield R et al. Changes in temperature alter the relationship between biodiversity and ecosystem functioning. Proc Natl Acad Sci USA 2018; 115: 10989–94. 10.1073/pnas.1805518115 30297403 PMC6205462

[57] Canard EF, Mouquet N, Mouillot D et al. Empirical evaluation of neutral interactions in host-parasite networks. Am Nat 2014; 183: 468–79. 10.1086/675363 24642492

[58] Lohrer AM, Thrush SF, Gibbs MM. Bioturbators enhance ecosystem function through complex biogeochemical interactions. Nature 2004; 431: 1092–5. 10.1038/nature03042 15470385

[59] Louca S, Polz MF, Mazel F et al. Function and functional redundancy in microbial systems. Nat Ecol Evol 2018; 2: 936–43. 10.1038/s41559-018-0519-1 29662222

[60] Allison SD, Martiny JB. Resistance, resilience, and redundancy in microbial communities. Proc Natl Acad Sci USA 2008; 105: 11512–9. 10.1073/pnas.0801925105 18695234 PMC2556421

[61] Wankmüller FJP, Delval L, Lehmann P et al. Global influence of soil texture on ecosystem water limitation. Nature 2024; 635: 631–8. 10.1038/s41586-024-08089-2 39443806 PMC11578876

[62] Dequiedt S, Saby NPA, Lelievre M et al. Biogeographical patterns of soil molecular microbial biomass as influenced by soil characteristics and management. Glob Ecol Biogeogr 2011; 20: 641–52. 10.1111/j.1466-8238.2010.00628.x

[63] Lacroix EM, Frei J, van der Loo E et al. Root exudation and fine texture interact to form anoxic microsites in rhizosphere soil. Soil Biol Biochem 2025; 211: 109974. 10.1016/j.soilbio.2025.109974

[64] Angst G, Pokorný J, Mueller CW et al. Soil texture affects the coupling of litter decomposition and soil organic matter formation. Soil Biol Biochem 2021; 159: 108302. 10.1016/j.soilbio.2021.108302

[65] Dominguez J, Aira M, Kolbe AR et al. Changes in the composition and function of bacterial communities during vermicomposting may explain beneficial properties of vermicompost. Sci Rep 2019; 9: 9657. 10.1038/s41598-019-46018-w 31273255 PMC6609614

[66] Chatterjee R, Debnath A, Mishra S. Vermicompost and soil health. In: Giri B, Varma A (eds.). Soil Health. Chem: Springer, 2020, 69–88.

[67] Zhu B, Whalen JK, Wu J et al. Soil food web structure coordinated by soil omnivores sustains soil multifunctionality in moderate vermicompost amended fields. Soil Biol Biochem 2024; 192: 109391. 10.1016/j.soilbio.2024.109391

[68] Crowther TW, Van Den Hoogen J, Wan J et al. The global soil community and its influence on biogeochemistry. Science 2019; 365: eaav0550. 10.1126/science.aav0550 31439761

[69] Allison SD . Microbial drought resistance may destabilize soil carbon. Trends Microbiol 2023; 31: 780–7. 10.1016/j.tim.2023.03.002 37059647

[70] Guo L, Ju C, Xu X et al. Unveiling pervasive soil microbial P limitation in terrestrial ecosystems worldwide. Ecol Lett 2024; 27: e70011. 10.1111/ele.70011 39623735

[71] Chai YN, Schachtman DP. Root exudates impact plant performance under abiotic stress. Trends Plant Sci 2021; 27: 80–91. 10.1016/j.tplants.2021.08.003 34481715

[72] Lambers H, Shane MW, Cramer MD et al. Root structure and functioning for efficient acquisition of phosphorus: matching morphological and physiological traits. Ann Bot 2006; 98: 693–713. 10.1093/aob/mcl114 16769731 PMC2806175

[73] Maestre FT, Delgado-Baquerizo M, Jeffries TC et al. Increasing aridity reduces soil microbial diversity and abundance in global drylands. Proc Natl Acad Sci USA 2015; 112: 15684–9. 10.1073/pnas.1516684112 26647180 PMC4697385

[74] Begill N, Schweizer SA, Don A et al. Increased retention of litter-derived organic carbon with increasing initial carbon content in temperate agricultural soils. Glob Change Biol 2025; 31: e70646. 10.1111/gcb.70646 PMC12683473 41355724

[75] Hu Z, Delgado-Baquerizo M, Fanin N et al. Nutrient-induced acidification modulates soil biodiversity–function relationships. Nat Commun 2024; 15: 2858. 10.1038/s41467-024-47323-3 38570522 PMC10991381

[76] Grunzweig JM, De Boeck HJ, Rey A et al. Dryland mechanisms could widely control ecosystem functioning in a drier and warmer world. Nat Ecol Evol 2022; 6: 1064–76. 10.1038/s41559-022-01779-y 35879539

[77] Page MJ, McKenzie JE, Bossuyt PM et al. The PRISMA 2020 statement: an updated guideline for reporting systematic reviews. Syst Rev 2021; 10: 89. 10.1186/s13643-021-01626-4 33781348 PMC8008539

[78] Hedges LV, Gurevitch J, Curtis PS. The meta-analysis of response ratios in experimental ecology. Ecology 1999; 80: 1150–6. 10.1890/0012-9658(1999)080[1150:TMAORR]2.0.CO;2

[79] Spake R, Mori AS, Beckmann M et al. Implications of scale dependence for cross-study syntheses of biodiversity differences. Ecol Lett 2021; 24: 374–90. 10.1111/ele.13641 33216440

[80] Lajeunesse MJ, Fitzjohn R. Facilitating systematic reviews, data extraction and meta-analysis with the metagear package for R. Methods Ecol Evol 2015; 7: 323–30. 10.1111/2041-210X.12472

[81] Lajeunesse MJ . On the meta-analysis of response ratios for studies with correlated and multi-group designs. Ecology 2011; 92: 2049–55. 10.1890/11-0423.1 22164829

[82] Zhang P, Li B, Wu J et al. Invasive plants differentially affect soil biota through litter and rhizosphere pathways: a meta-analysis. Ecol Lett 2019; 22: 200–10. 10.1111/ele.13181 30460738

[83] R Core Team . R: A Language and Environment for Statistical Computing. R Foundation for Statistical Computing, Vienna, Austria, 2023.

