# Supplementary material for: Soil biota abundance supports ecosystem multifunctionality under carbon farming
Source: Natl Sci Rev. 2026 Apr 29;13(10):nwag247. doi: 10.1093/nsr/nwag247 (PMC13248868; doi:10.1093/nsr/nwag247)
Supplement: nwag247_Supplemental_Files [file nwag247_supplemental_files.zip › Supplementary Information.docx]

Supplementary Materials for

**Soil biota abundance supports ecosystem multifunctionality under carbon farming**

Wenfeng Xue^1,2#^, Ting Liu^2#*^, Saisai Cheng^1#^, Xiaoyun Chen^2^, Joann K. Whalen^3,4^ & Manqiang Liu^1,2*^

^1^Centre for Grassland Microbiome, State Key Laboratory of Herbage Improvement and Grassland Agro-Ecosystems, College of Pastoral Agriculture Science and Technology, Lanzhou University, Lanzhou 730000, China

^2^Soil Ecology Lab, College of Resources and Environmental Sciences, Nanjing Agricultural University, Nanjing 210095, China

^3^Department of Natural Resource Sciences, McGill University, Montreal, Quebec, H9X 3V9, Canada

^4^College of Agriculture, Mohammed VI Polytechnic University, Lot 6620, Hay Moulay Rachid 43150, Ben Guerir, Morocco

^#^These authors contributed equally to this work.

^*^Correspondence: liumq@njau.edu.cn (Manqiang Liu); ting.liu@njau.edu.cn (Ting Liu)

**Table of Contents**

**Supplementary Methods**4

**Supplementary Figures**4

Supplementary Fig. 1. Conceptual cascade framework for agroecosystem multifunctionality analysis 13

Supplementary Fig. 2. Agroecosystem variable responses to plant- and animal-based carbon inputs in a global meta-analysis 14

Supplementary Fig. 3. Sample effect sizes of soil biota abundance and richness under plant- and animal-based carbon inputs 15

Supplementary Fig. 4. Relationships between model-predicted and the observed effects of soil biota abundance 16

Supplementary Fig. 5. Soil biota abundance responses to interactions between environmental variables and substrate characteristics 17

Supplementary Fig. 6. Conceptual framework for optimizing carbon farming strategy under different environmental conditions 18

Supplementary Fig. 7. PRISMA flow diagram showing the literature search and study screening procedure for the meta-analysis of agroecosystem multifunctionality under plant- and animal-based carbon inputs 19

Supplementary Fig. 8. Graphical illustration of the dataset structure and three-level hierarchical model 20

Supplementary Fig. 9. Comparison of agroecosystem variable responses to plant- and animal-based carbon inputs across different measurement methods 21

Supplementary Fig. 10. Alternative analytical treatments did not change the responses of key agroecosystem functions to plant- and animal-based carbon inputs 22

**Supplementary Tables**23

Supplementary Table 1. List of ecosystem variables included in the meta-analysis 23

Supplementary Table 2. Effect size estimates and model-based sensitivity analysis for the plant-based carbon inputs using multilevel and simple random-effects models 26

Supplementary Table 3. Effect size estimates and model-based sensitivity analysis for the animal-based carbon inputs using multilevel and simple random-effects models 27

Supplementary Table 4. Proportions of pairwise functional outcomes (win-win, trade-off, and lose-lose) between soil biota abundance or richness and individual ecosystem functions under plant- and animal-based carbon inputs 28

Supplementary Table 5. Summary of site and environmental characteristics for plant- and animal-based carbon inputs included in the meta-analysis 29

Supplementary Table 6. Multilevel meta-analysis of soil biota abundance responses to moderator interactions (climate factors × soil properties, climate factors × substrate characteristics, and soil properties × substrate characteristics) under plant-based carbon inputs30

Supplementary Table 7. Multilevel meta-analysis of soil biota abundance responses to moderator interactions (climate factors × soil properties, climate factors × substrate characteristics, and soil properties × substrate characteristics) under animal-based carbon inputs 35

Supplementary Table 8. Summary of moderator interaction effects on soil biota abundance under plant- and animal-based carbon inputs 40

Supplementary Table 9. Comparison of random-effects and mixed-effects models for soil biota abundance under plant-based carbon inputs 41

Supplementary Table 10. Comparison of random-effects and mixed-effects models for soil biota abundance under animal-based carbon inputs 43

Supplementary Table 11. Heterogeneity tests (Q statistics) for soil biota abundance under plant- and animal-based carbon inputs 45

**Supplementary References**46

**Supplementary Methods**

***Detailed literature search, study screening, and inclusion criteria***

We conducted the literature search and study screening in three steps.

First, we used a combination of keywords synonymous with cover crops (such as ‘catch crop’ and ‘green manure’) and specific keywords related to vermicompost. This search yielded 17,879 and 5,085 records for cover crops and vermicompost, respectively, including both peer-reviewed journal articles and theses.

Second, we scanned the titles and abstracts of each publication to remove duplicates, irrelevant studies and non-experimental papers (e.g., modeling studies, reviews/meta-analyses, book chapters, and proceedings). In this step, we excluded 3,528 records. The remaining 19,436 publications (15,108 for cover crops and 4,328 for vermicompost) were retained for further evaluation.

Third, candidate papers were examined individually to confirm that the data met the following criteria: (1) Only field studies were retained, whereas studies conducted in greenhouses or in vitro were excluded; (2) Studies had to include both control and treatment groups, with at least three replicate plots per group. Legume cover crop studies were required to include a no-cover-crop control and at least one identified legume cover crop treatment. Vermicompost studies had to include a vermicompost-free control and at least one level of vermicompost input; (3) Legume cover crop studies had to involve on-site cultivation and retention of plant residues, as commonly practiced in no-tillage, reduced tillage, and conventional tillage systems. This criterion was applied because the contribution of legume cover crops as plant carbon sources is expressed during both the growth phase and the decomposition of fresh residues, thereby affecting ecosystem functions. Intercropping studies were excluded to avoid potential interspecific competition effects from other crops; (4) Soil sampling had to focus on bulk soil, excluding studies of rhizosphere soil. Soil samples had to be collected from the top 0-25 cm of soil; (5) Studies had to report at least one ecosystem function variable in both control and treatment groups under the same conditions; (6) When a response variable was measured at multiple times, only the final measurement was used; (7) Data were used only once if the same dataset appeared in multiple papers; and (8) when a publication included experiments conducted at multiple sites over different time periods, each independent experiment was treated as a separate study (Table S1).

In each study, the treatment group was compared with the control group only when the two groups differed solely in the use of legume cover crops or vermicompost (e.g., identical tillage regime, synthetic fertilizer and pesticide application), and were sampled simultaneously. This design helped reduce confounding arising from differences among studies in environmental conditions, management practices, or soil properties. When no control group matched the fertilization level exactly, we selected the control group with the highest fertilization level. This approach was justified because demonstrating that carbon farming practices can enhance multifunctionality under reduced or no fertilization would support their adoption in sustainable agriculture and reduce reliance on synthetic fertilizers. Based on these criteria, we selected 475 studies for legume cover crops and 198 studies for vermicompost (see Source Data file for the full list of references).

***Detailed variable classification and contextual data extraction***

We extracted 53 ecosystem variables to characterize nine ecosystem functions: soil biota richness, soil biota abundance, soil activity, soil fertility, soil physical structure, water regulation, climate regulation, plant productivity, and product quality. These functions were further grouped into three service categories, including supporting, regulating and provisioning services, following the MA framework [1,2] (Fig. S1).

Soil biota richness was represented by four components: bacterial richness, fungal richness, nematode richness and earthworm richness. Soil biota abundance was quantified as absolute abundance (i.e., number of individuals or density) for these major groups. Soil activity was assessed using variables including MBC, MBN, soil basal respiration, α-glucosidase (AG), β-glucosidase (BG), cellobiohydrolase (CB), β-xylosidase (XYL), L-leucine aminopeptidase (LAP), β-1,4-N-acetylglucosaminidase (NAG) and phosphatase (PHOS). Soil fertility was evaluated using variables such as soil organic C, soil total N, soil total P, dissolved organic C, soil available N, soil nitrate, soil ammonium, soil available P, soil moisture, water-holding capacity, soil pH, and soil EC. Soil physical structure was represented by soil bulk density. Water regulation included N and P leaching potential, calculated from the concentrations of NH_4_-N, NO_3_-N, and P in the leachate together with the total leachate volume collected during the case crop growth period. Climate regulation included cumulative CH_4_, CO_2_ and N_2_O emissions per unit area over the same period. Plant productivity was quantified using above- and below-ground biomass and crop yield. Product quality was assessed using C and nutrient (N, P) concentrations, soluble protein, soluble sugars, vitamin C, organic acids, sugars-acids ratio, and nitrate content in harvested products.

We also recorded contextual information for each study, including the site location (latitude and longitude), climate variables (MAT and MAP), pre-experiment soil properties (fine texture, expressed as the percentage of clay plus silt; soil organic C; soil total N; soil pH; and soil C/N ratio), and substrate characteristics (substrate N, P, C content; total substrate N, P, C inputs; and substrate C/N, N/P, C/P ratios) (Table S5). These data were extracted from the main text, tables, or figures of each publication, with figure data digitized using WebPlotDigitizer. When geographical locations were not provided, latitude and longitude were obtained from Google Maps using the location names provided in the articles; when only country names were reported, we used the coordinates of the capital city. When climate variables or background soil properties were unavailable, they were extracted from the WorldClim and SoilGrids databases, respectively, using geographical information. Substrate characteristics were recorded as missing when unavailable. All extracted data were double-checked to minimize transcription errors.

***Detailed definitions and measurements of soil biota richness and abundance***

For soil biota richness, we considered four components: bacterial richness, fungal richness, nematode richness, and earthworm richness. Bacterial richness was assessed as the number of operational taxonomic units (OTUs) using 16S rRNA genes and phospholipid fatty acids (PLFAs) methods. Fungal richness was quantified as the number of OTUs based on ITS sequencing. Nematode richness was determined by counting and identifying genera, whereas earthworm richness was determined by manual sorting. We excluded biodiversity metrics such as Shannon-Wiener and Simpson indices because they are derived metrics that combine information on richness and abundance [3]. Because bacterial and fungal richness were measured using different methods, we conducted supplementary analyses to assess the appropriateness of pooling these methods (described below).

For soil biota abundance, we used the absolute abundance of each major group, that is, the number of individuals or density of soil bacteria, fungi, nematodes and earthworms. This metric was preferred over relative abundance because it provides a more direct basis for comparison across groups. Bacterial and fungal abundance were determined using qPCR and PLFAs-based measurements of absolute abundance. Nematode abundance was quantified by counting the number of individuals per 100 g dry soil for each functional group (bacterivore, fungivore, herbivore, omnivore and predator) or for the overall community (total nematodes). Earthworm abundance was determined by counting earthworm density (individuals m^-2^).

***Calculation of effect size***

To standardize comparisons across results, we used the natural log-transformed response ratio (ln*RR*) [4] as the effect-size metric for comparing means between treatment and control groups. ln*RR* was chosen because it is relatively robust to non-independence and small sample sizes [5], and is widely used in meta-analysis. It was calculated as follows:

ln*RR* = ln (X_t_/X_c_) (Equation 1)

where X_t_ and X_c_ represent the mean values of agroecosystem variables in the treatment groups (i.e., with legume cover crop or vermicompost inputs) and control groups (i.e., without these inputs), respectively.

For ecosystem variables such as soil bulk density, N leaching potential, P leaching potential, CO_2_ emissions, N_2_O emissions and fruit nitrate, where lower values indicate better ecosystem quality, ln*RR* values were multiplied by −1 to ensure that greater values consistently represented enhanced ecosystem functions and services [6]. The variance (vi) of each effect size was calculated as follows:

$\text{ vi = }\frac{{\text{S}_{\text{t}}}^{\text{2}}}{\text{n}_{\text{t}}{\text{X}_{\text{t}}}^{\text{2}}}\text{+ }\frac{{\text{S}_{\text{c}}}^{\text{2}}}{\text{n}_{\text{c}}{\text{X}_{\text{c}}}^{\text{2}}}$ (Equation 2)

where S_t_ and S_c_ are the standard deviations (SDs) of X_t_ and X_c_, respectively, while n_t_ and n_c_ are the corresponding sample sizes. If studies reported standard errors (SEs) instead of SDs, SDs were calculated as SD = SE × $\sqrt{\text{n}}$. When neither SD nor SE was reported, the ‘Bracken1992’ approach in the R package ‘*metagear*’ was used to impute SDs based on the coefficient of variation from all cases with complete variance information [7]. Effect sizes were calculated using the ‘escalc’ function in the R package ‘*metafor*’ [8].

***Calculation of mean effect size***

To assess the effects of legume and vermicompost treatments on individual ecosystem functions, ecosystem services, and overall ecosystem performance (i.e., multifunctionality), we calculated the overall mean effect sizes (ln*RR*_++_) with 95% CIs using inverse-variance-weighted mixed-effects models in the ‘*metafor*’ package. Effect sizes were considered statistically significant when the 95% CIs did not overlap zero. ln*RR*_++_ was calculated as follows [4]:

$\text{ }\text{ln}\text{RR}\text{++}\text{ = }\frac{\sum_{i=1}^{m} \sum_{j=1}^{n} \frac{1}{v_{ij}}{\ln RR}_{ij}}{\sum_{i=1}^{m} \sum_{j=1}^{n} \frac{1}{v_{ij}}}$ (Equation 3)

where ‘m’ and ‘n’ represent the numbers of treatment and control groups, respectively, and ln*RR*_ij_ and v_ij_ represent the effect size and its variance for each response in each case study.

A major objective of meta-analysis is to calculate mean or pooled effect sizes across studies [9,10]. Ensuring independence among observations is an important prerequisite for pooling effect sizes and conducting meta-regression analysis [11]. To account for heterogeneity both between and within studies, we conducted a three-level hierarchical meta-analysis random-effects model. In our dataset, many studies reported multiple effect sizes associated with different levels of legume or vermicompost treatment, experimental sites, or categories of ecosystem variables. This created a hierarchical structure of effect sizes, as illustrated in Fig. S8, with the overall pooled effect size at the top (level 3), followed by study-specific effect sizes at the intermediate level (level 2), and replicate-level effect sizes at the bottom (level 1).

Within this framework, we used a three-level model to calculate pooled effect sizes for each combination of treatment group, environmental site, and category of ecosystem variables. The model was expressed as follows [12]:

$\hat{{lnRR}_{ijk}}=\mu+ \theta_{\left( 3 \right)i}+ \zeta_{\left( 2 \right)ij}+ \varepsilon_{ijk}$ (Equation 4)

Here, $\hat{{lnRR}_{ijk}}$ is the estimated effect size, *µ* is the overall mean effect size, Ɛ_𝑖𝑗𝑘_ represents level 1 heterogeneity caused by sampling errors, 𝜁_(2)𝑖𝑗_ represents the level 2 heterogeneity within studies, and 𝜃_(3)𝑖_ represents level 3 heterogeneity among studies. Briefly, the random-effects structure included study identity (i.e., study ID) and the effect-size identity (i.e., effect size ID) nested within study. This three-level model explicitly accounted for non-independence among effect sizes.

Because some studies reported multiple treatment levels sharing a common control, the assumption of independence among effect sizes could be violated [13]. To account for this, we partitioned such cases into independent control-treatment pairs and calculated a variance-covariance (VCV) matrix for each dataset [14]. The VCV matrix accounts for the dependency created by shared controls when multiple effect sizes are estimated from the same experiment [15,16]. The inverse of the sampling variance (vi) in the VCV matrix was used to weight the precision of the effect size [14]. For two treatment groups (A and B) sharing a common control, the VCV matrix was calculated as follows:

$\text{ VCV = }\left[ \begin{matrix} \frac{{\text{S}_{\text{t}}}^{\text{2}}}{\text{n}_{\text{t}}{\text{(X}_{\text{t}}^{\text{A}}\text{)}}^{\text{2}}}\text{+ }\frac{{\text{S}_{\text{c}}}^{\text{2}}}{\text{n}_{\text{c}}{\text{X}_{\text{c}}}^{\text{2}}} & \frac{{\text{S}_{\text{t}}}^{\text{2}}}{\text{n}_{\text{c}}{\text{X}_{\text{c}}}^{\text{2}}} \\ \frac{{\text{S}_{\text{c}}}^{\text{2}}}{\text{n}_{\text{c}}{\text{X}_{\text{c}}}^{\text{2}}} & \frac{{\text{S}_{\text{t}}}^{\text{2}}}{\text{n}_{\text{t}}{\text{(X}_{\text{t}}^{\text{B}}\text{)}}^{\text{2}}}\text{+ }\frac{{\text{S}_{\text{c}}}^{\text{2}}}{\text{n}_{\text{c}}{\text{X}_{\text{c}}}^{\text{2}}} \end{matrix} \right]$ (Equation 5)

where S_t_ and S_c_ represent the standard deviations (SDs) of X_t_ and X_c_, respectively, while n_t_ and n_c_ are the corresponding sample sizes. To facilitate interpretation, ln*RR* values were back-transformed into percentage change as follows [4,17]:

$\text{ Percentage change }\left( \text{\%} \right)\text{ = }\left( \text{e}^{{\text{ln}\text{RR}}_{\text{++}}}\text{-1} \right)\text{ ×100\%}$ (Equation 6)

Percentage change and response ratio describe different aspects of treatment effects. Percentage change quantifies effect size in intuitive percentage terms, whereas ln*RR* was used as the statistical metric for significance testing. Multilevel hierarchical random-effects meta-analyses were conducted using the rma.mv() function in the ‘*metafor*’ package with restricted maximum likelihood (REML) estimation, weighted by sampling variance.

***Publication bias and supplementary analyses***

Publication bias is a potential concern in meta-analyses because statistically significant results are more likely to be published, which can bias pooled effect-size estimates [18]. To evaluate its potential influence, we conducted multiple supplementary analyses for each main effect-size metric. First, we evaluated publication bias using Rosenthal’s fail-safe number [19] and Egger’s regression test [20]. A fail-safe number, which estimates the number of null-effect sizes required to render the overall effect non-significant, is generally considered relatively robust to publication bias when it exceeds 5𝑛 + 10, where 𝑛 is the number of observed effect sizes [19]. Second, we also compared random-effects models with (multilevel models) and without modelling non-independence (simple models), and assessed the impact of potential publication bias on simple models by the trim and fill method [18]. Third, although all ecosystem variables followed standardized protocols, the same variable could be measured using different technical methods across studies (Table S1). To assess whether measurement methods influenced our results, we conducted cumulative meta-analyses (Fig. S9), treating method as a fixed effect, and effect size nested within study as a random effect.

Although publication bias was detected for some variables, the corrected effect sizes obtained using the trim and fill method retained the same direction as the original results (Tables S2 and S3), indicating that our overall conclusions were robust to publication bias. Methodological differences also had no significant effect on most results, except for fungal abundance under plant-based carbon inputs and bacterial abundance and soil available N under animal-based carbon inputs. To assess whether these methodological differences could affect our overall conclusions, we conducted additional analyses focused on soil biota abundance. For the plant-based carbon inputs dataset, we repeated cumulative meta-analyses after separately excluding subsets in which fungal abundance was measured using qPCR or PLFAs methods (Fig. S10a). For the animal-based carbon inputs dataset, we repeated the analysis after excluding subsets in which bacterial abundance or soil available N had been measured using different methods (Fig. S10b). In both cases, the effect sizes remained unchanged, indicating that methodological differences did not alter the overall conclusions. These analyses were performed using the ‘*metafor*’ package.

***Assessing the relative contribution of soil biota abundance and richness to agroecosystem multifunctionality***

We applied two complementary approaches to assess the relative contributions of soil biota abundance and richness to multifunctionality. Prior to analysis, when multiple effect sizes were available for the same function within the same treatment-control comparison, they were averaged to obtain one mean effect size per function.

First, we used a covariance-based decomposition analysis to compare the relative importance of soil biota abundance, soil biota richness, and the other ecosystem functions for multifunctionality. For each function (*f_i_*), the contribution share was calculated as:

$\text{S}_{\text{i}}\text{ = }\frac{\text{Cov}\text{(}\text{f}_{\text{i}}\text{, }\text{MF}\text{)}}{\sum_{\text{j}\text{=1}}^{\text{9}} \text{Cov}\text{(}\text{f}_{\text{j}}\text{, }\text{MF}\text{)}}$ (Equation 7)

where *S_i_* represents the proportional contribution of function i, Cov(*f_i_*, *MF*) is the covariance between function 𝑖 and multifunctionality, and the denominator is the summed covariance across all nine functions. This analysis partitions the covariance structure of multifunctionality across functions and expresses their relative contributions as proportions of total covariance, allowing comparison among functions while minimizing potential bias arising from structural overlap in index construction.

To directly compare soil biota abundance and richness, we calculated the difference in contribution share (*S_i_*) as:

Δ = *S*_abundance_ − *S*_richness_ (Equation 8)

where positive values indicate a stronger contribution of soil biota abundance than richness. Uncertainty was estimated using a study-level bootstrap procedure with 2,000 iterations. In each iteration, studies were resampled with replacement, all observations within each selected study were retained, and *S_i_* and Δ were recalculated. The 95% confidence intervals were derived from the 2.5th and 97.5th percentiles of the bootstrap distributions.

Second, we visualized trade-off, lose-lose, and win-win relationships by plotting pairwise combinations of effect sizes for soil biota abundance or richness and other individual ecosystem functions. Each paired observation consisted of one effect size for soil biota abundance or richness matched with the effect size of a single ecosystem function within the same study. This analysis was based on 504 paired observations for soil biota abundance (321 for legume cover crops and 183 for vermicompost) and 478 paired observations for soil biota richness (310 for legume cover crops and 168 for vermicompost). The percentage of observations in each category (trade-off, lose-lose, or win-win) was calculated as the proportion of all paired observations and displayed on a Cartesian plane [6].

***Assessing the determinants of soil biota abundance benefits for each carbon source***

We assessed the heterogeneity in soil biota abundance effect sizes using Cochran's Q statistic, calculated as the weighted sum of squares. The resulting values were compared with a chi-squared distribution to determine whether the variance in observed effect sizes explained by the model exceeded that expected by chance. A significant Q statistic indicates heterogeneity in effect sizes. Our analyses revealed substantial between-study heterogeneity in the effect sizes of soil biota abundance (*P* < 0.001, see Table S11 for details). We therefore used boosted regression tree (BRT) analysis and mixed-effect meta-regression analysis to explore potential moderators of soil biota abundance and explain the observed between-study heterogeneity.

We first used BRT analysis to assess the relative importance of potential moderators, including climate variables (MAT and MAP), pre-experiment soil properties (fine texture, soil organic C, soil total N, soil pH, and soil C/N ratio), and substrate characteristics (substrate N, P, C content; total substrate N, P, C inputs; and substrate C/N, N/P, C/P ratios), in shaping soil biota abundance. To minimize prediction error, the optimal number of trees was determined using 10-fold cross-validation. BRT parameters included a Gaussian error distribution, tree complexity of 2, learning rate of 0.001, step size of 50, and bag fraction of 0.75 [21]. The optimal model consisted of 5,850 regression trees for legume cover crops and 1,450 for vermicompost. The relative importance of each factor was quantified as its contribution to the overall model. All BRT analyses were conducted using the ‘*gbm*’ and ‘*dismo*’ packages.

We then conducted mixed-effect meta-regressions to assess the individual and interaction effects of climate factors, soil properties, and substrate characteristics on soil biota abundance (Table S5). Individual effects were evaluated using both linear models (f(*X*) = *β*_0_ + *β*_1_*X*) and quadratic models (i.e., f(*X*) = *β*_0_ + *β*_1_*X* + *β*_2_*X*^2^) to test for linear and nonlinear relationships between moderator variables and soil biota abundance. The random-effects and mixed-effects models are summarized in Tables S9 and S10. All models were fitted using maximum-likelihood estimation, and the significance of each fixed effect, including interaction terms, was evaluated using likelihood ratio tests (LRTs) [9]:

$\text{ L}\text{ = }\text{P}\text{ (}\text{D}\text{|}\text{θ}\text{, M}\text{)}$ (Equation 9)

$\text{ LRT = 2 ×}\text{ }\text{ln(}\frac{L(M_{full})}{L(M_{reduced})}\text{)}$ (Equation 10)

where 𝐿 is the likelihood of the observed data (𝐷) under the model 𝑀 with parameters 𝜃, and 𝐿(𝑀∗) denotes the likelihood of the corresponding model. 𝑀_𝑓𝑢𝑙𝑙_ refers to the model with more parameters (e.g., mixed-effects model), whereas 𝑀_𝑟𝑒𝑑𝑢𝑐𝑒𝑑_ refers to the model with fewer parameters (e.g., random-effects model). A significant LRT statistic (*P* < 0.05) indicates that the corresponding fixed effect improves model fit. For example, if the comparisons of *M_6_* versus *M_4_* and *M_6_* versus *M_5_* are both significant (Table S9), we infer that the relationship between MAP and soil biota abundance under legume cover crops is nonlinear. By contrast, if the comparison of *M_9_* versus *M_8_* is not significant but that of *M_8_* versus *M_7_* is significant, we infer a linear relationship.

We assessed the interaction effects among climate factors, initial soil properties and substrate characteristics on soil biota abundance using the mixed-effects meta-regression model (see Tables S6 and S7 for details):

$\text{ mods = \textasciitilde}\text{X}\text{1}\text{ }\text{+ }\text{X}\text{2}\text{ }\text{+ }\text{X}\text{1}\text{:}\text{X}\text{2}$ (Equation 11)

where *X_1_* and *X_2_* are fixed-effect predictors. We used the same random-effect structures as in the main meta-analyses. We considered only interactions between factors from different categories, such as climate factors, soil properties, and substrate characteristics, because these interactions provide more direct and practical guidance for land managers seeking to enhance soil biota abundance and agroecosystem multifunctionality. We then extracted the fixed-effect coefficients and their confidence intervals to quantify the strength and direction of the interaction effects and visualized the predicted response surfaces using the ‘visreg2d’ function from the ‘*visreg*’ package [22] and the ‘ggPredict’ function from the ‘*ggiraphExtra*’ package. These visualizations show how the effect of one predictor on soil biota abundance changes along the gradient of the interacting variable. They were generated from the fitted model parameters and did not require refitting the original rma.mv model.

To further characterize the interaction surfaces, we derived transition points from the fitted interaction models. For models of the form $y= \beta_{0}+ \beta_{1}\text{X}\text{1}+\beta_{2}\text{X}\text{2}+ \beta_{3}\text{X}\text{1}\text{X}\text{2}$, the marginal effect of each predictor depends on the value of the interacting variable. Transition points were defined as the predictor values at which the marginal effect equals zero, indicating that the direction of the predictor-response relationship may change depending on the interacting factor (i.e., $\beta_{2}+ \beta_{3}\text{X}\text{1}= 0$ or $\beta_{1}+ \beta_{3}\text{X}\text{2}= 0$).

In meta-regression, total heterogeneity can be partitioned into the variance explained by moderators (Q_m_, Q-statistic) and the residual error variance (Q_e_). The Q_m_ statistic is a Wald-type test of model coefficients, and a significant Q_m_ statistic indicates that the moderator explains a significant portion of the variation in soil biota abundance effect sizes [8].

***Predicting global benefits of carbon farming for soil biota abundance***

To extend our findings to global croplands, we used a machine-learning approach to generate spatially explicit predictions of the potential benefits of carbon farming for soil biota abundance worldwide. Global cropland regions were defined using the 2019 global cropland database (https://glad.umd.edu/dataset/croplands) [23]. We developed BRT models to upscale the estimated effects of carbon farming on soil biota abundance while accounting for spatial variation in climate and soil properties. The models incorporated climate variables (MAT and MAP) and pre-experiment soil properties (fine texture, SOC, TN, soil pH, and C/N ratio) as predictors to capture nonlinear relationships and interactions. The final optimized models consisted of 7,100 regression trees for legume cover crops and 2,550 for vermicompost, using the same BRT parameter settings described above. The fitted BRT models were subsequently applied to global predictor datasets to estimate the effects of plant- and animal-based carbon inputs on soil biota abundance. Global gridded soil data, including clay content, silt content, soil organic C, soil total N, and soil pH, were obtained from the SoilGrids database. Fine texture was derived from the global clay and silt grids, and gridded soil C/N ratios were calculated from soil organic C and total N using the ‘*raster*’ package.

**Supplementary Figures**

**
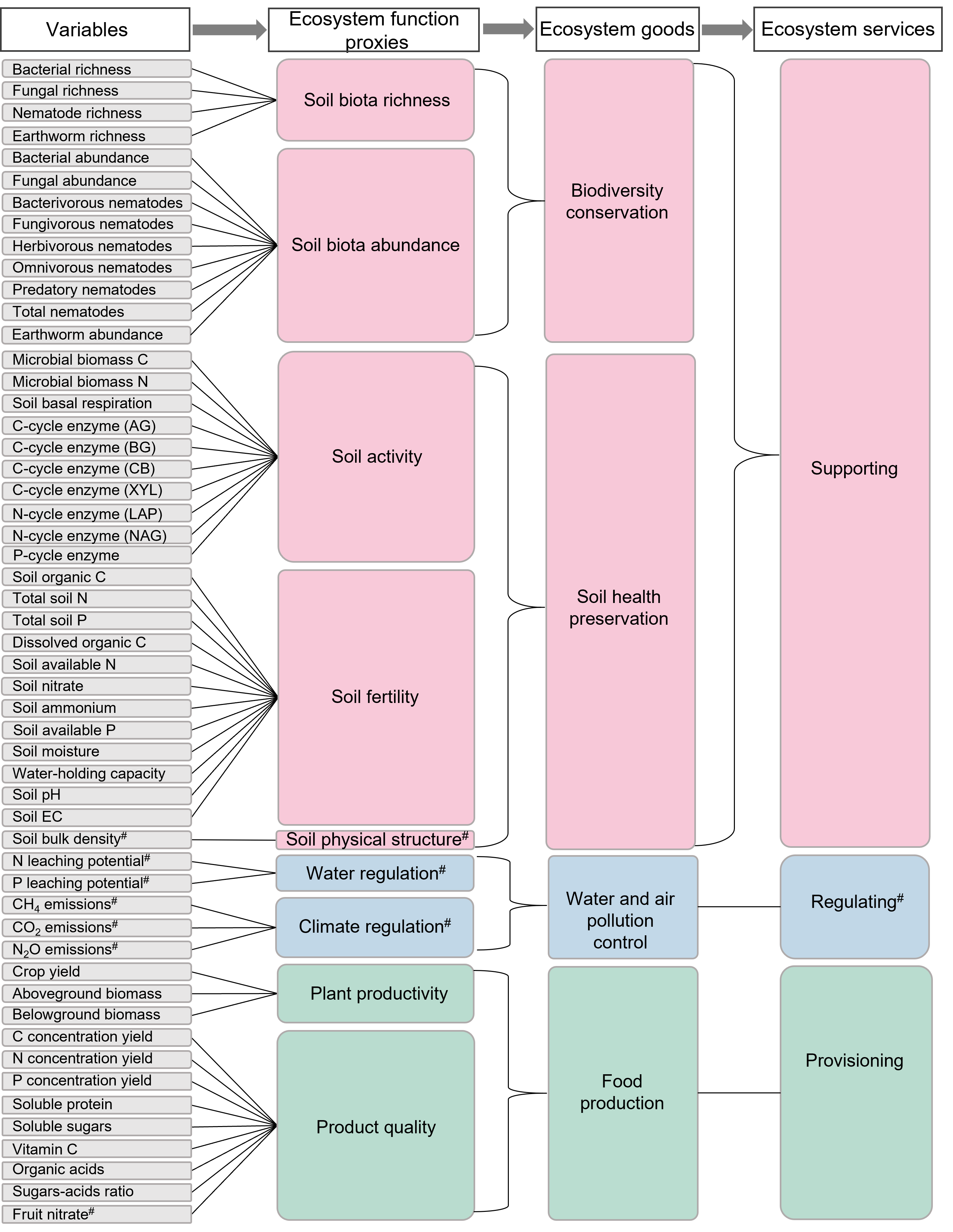
**

**Supplementary Fig. 1. Conceptual cascade framework for agroecosystem multifunctionality analysis.** Variables on the left were used individually or in combination as proxies for ecosystem functions and grouped into the supporting, regulating, and provisioning categories of the Millennium Ecosystem Assessment. ^#^, values multiplied by -1 to ensure directional consistency, with higher values indicating a more desirable state. In total, 53 ecosystem variables were included in this meta-analysis. This figure was adapted from the framework outlined in a previous study [24].

**
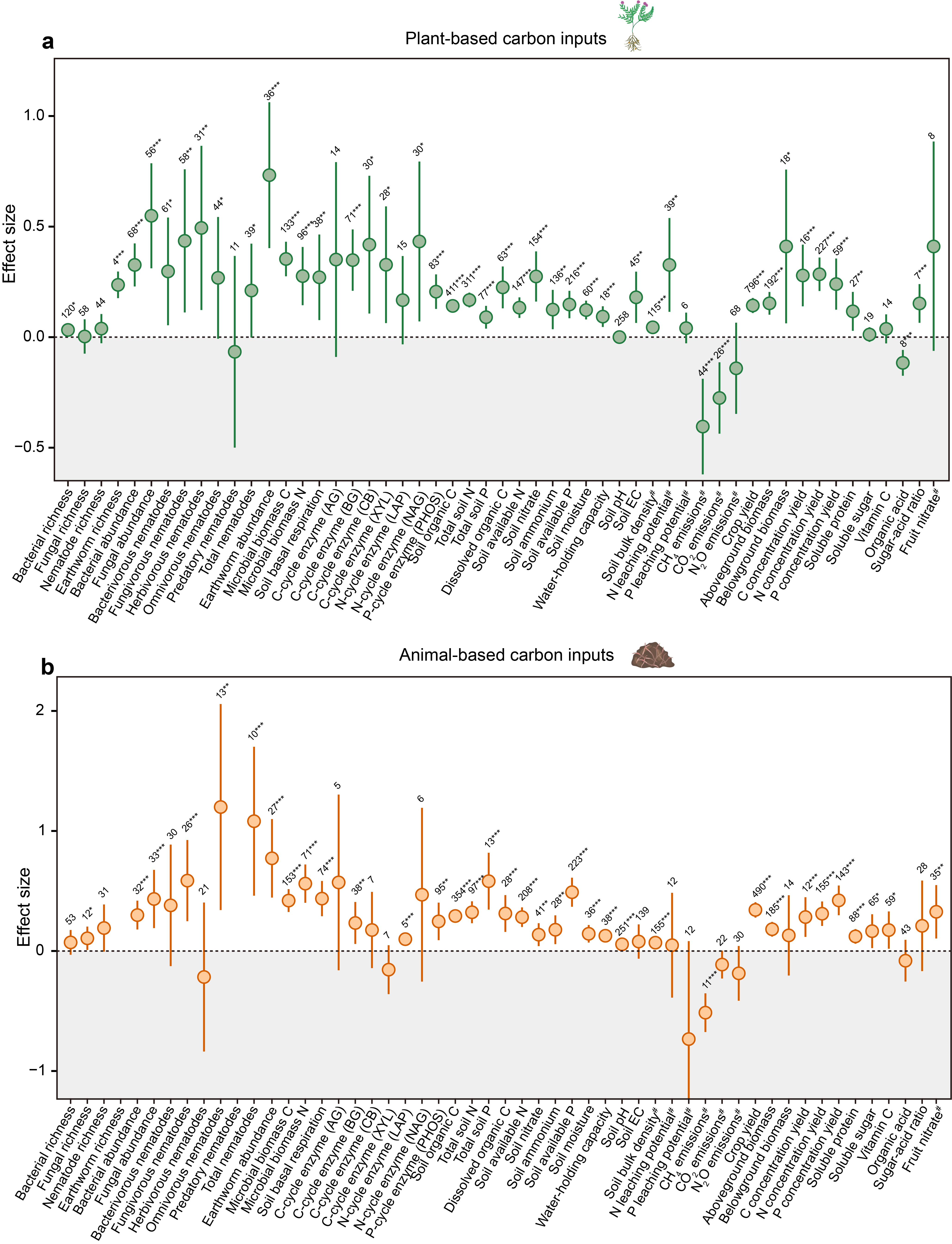
**

**Supplementary Fig. 2.** **Agroecosystem variable responses to plant- and animal-based carbon inputs in a global meta-analysis.** Effect sizes of plant-based carbon inputs (legume cover crops, a) and animal-based carbon inputs (vermicompost, b) on 53 ecosystem variables. Values represent mean effect sizes with 95% confidence intervals (CIs). Numbers indicate sample sizes. Mean effect sizes were considered significant when the 95% CIs did not overlap zero (**P* < 0.05, ***P* < 0.01, and ****P* < 0.001).

**
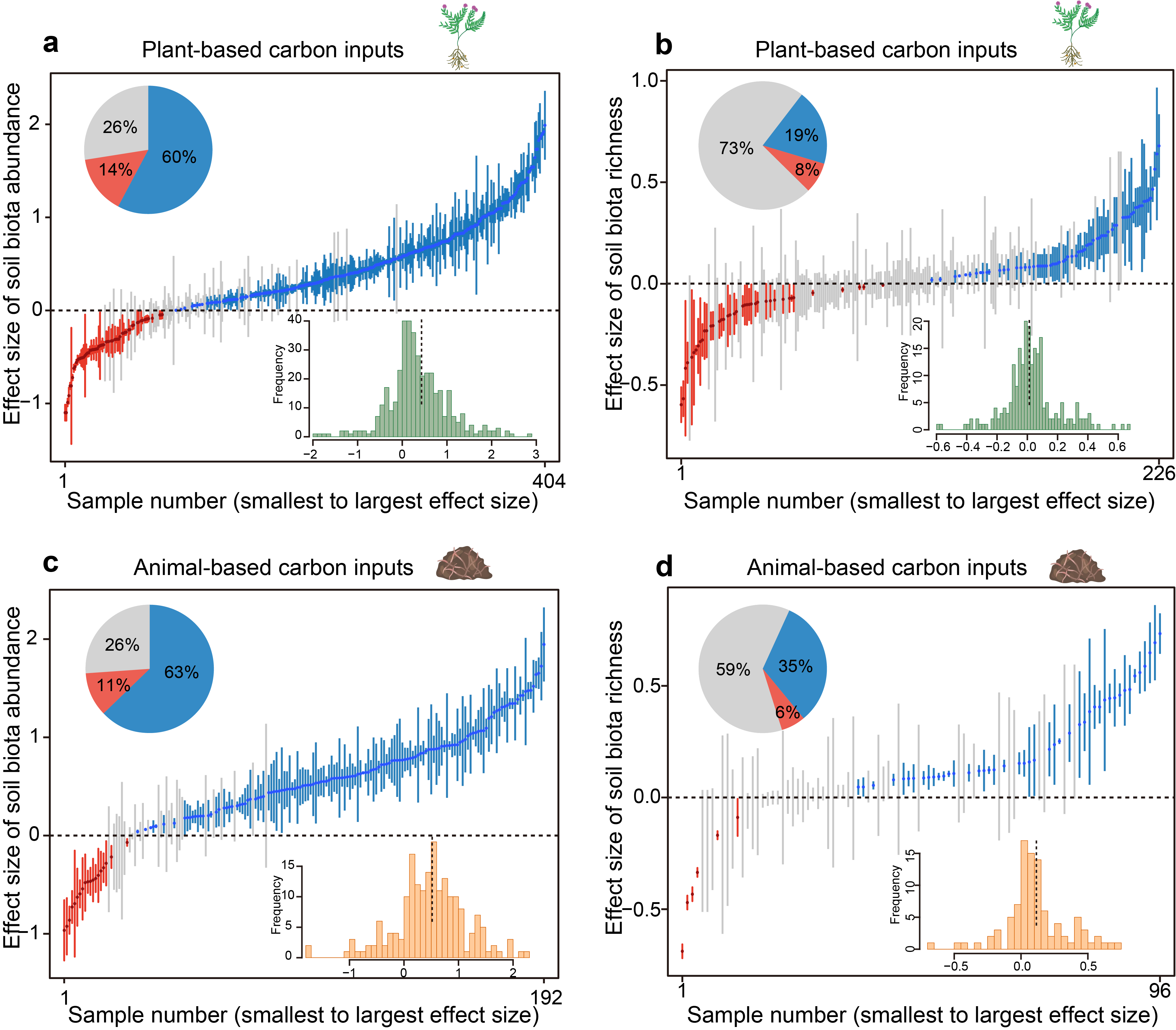
**

**Supplementary Fig. 3. Sample effect sizes of soil biota abundance and richness under plant- and animal-based carbon inputs.** Sample effect sizes of plant-based carbon inputs (legume cover crops, a-b) and animal-based carbon inputs (vermicompost, c-d) for soil biota abundance and richness, shown with 95% confidence intervals (CIs). Blue and red points indicate significantly positive and negative effects, respectively (*P* < 0.05). Pie charts (upper left inset) show the number of samples with effect sizes greater than zero (blue, positive effect), less than zero (red, negative effect), or not significantly different from zero (gray). Histogram plots (lower right inset) show the frequency distribution of sample effect sizes.


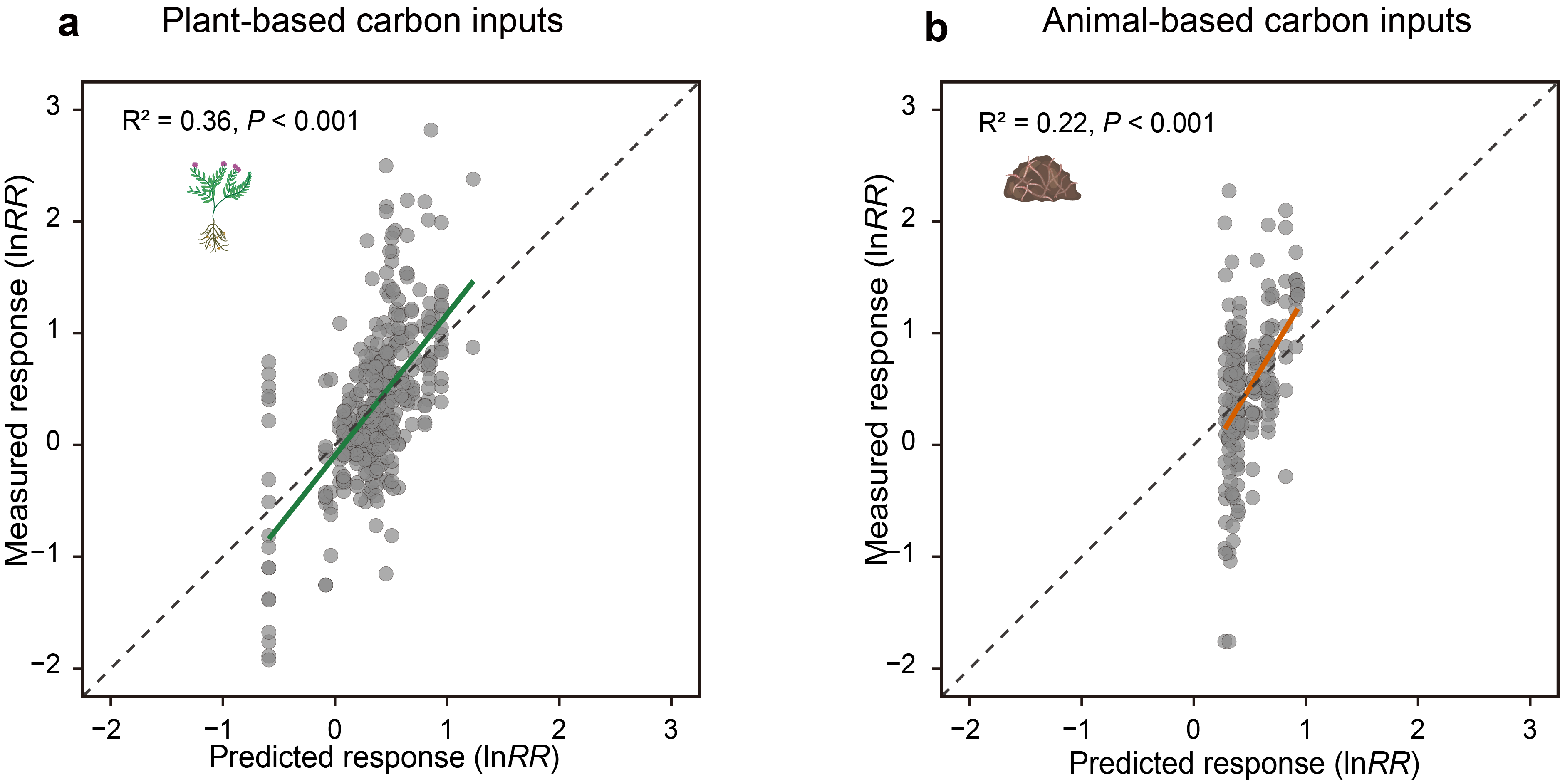


**Supplementary Fig. 4.** **Relationships between model-predicted and the observed effects of soil biota abundance.** (a) Relationship under plant-based carbon inputs: observed effect of soil biota abundance (ln*RR*) = 1.26 × predicted effect of soil biota abundance (ln*RR*) - 0.09, R^2^ = 0.36, *P* < 0.001, N = 404. (b) Relationship under animal-based carbon inputs: observed effect of soil biota abundance (ln*RR*) = 1.65 × predicted effect of soil biota abundance (ln*RR*) - 0.311, R^2^ = 0.22, *P* < 0.001, N = 192. Green and orange lines show the fitted relationships, and the dashed gray line indicates the 1:1 relationship.


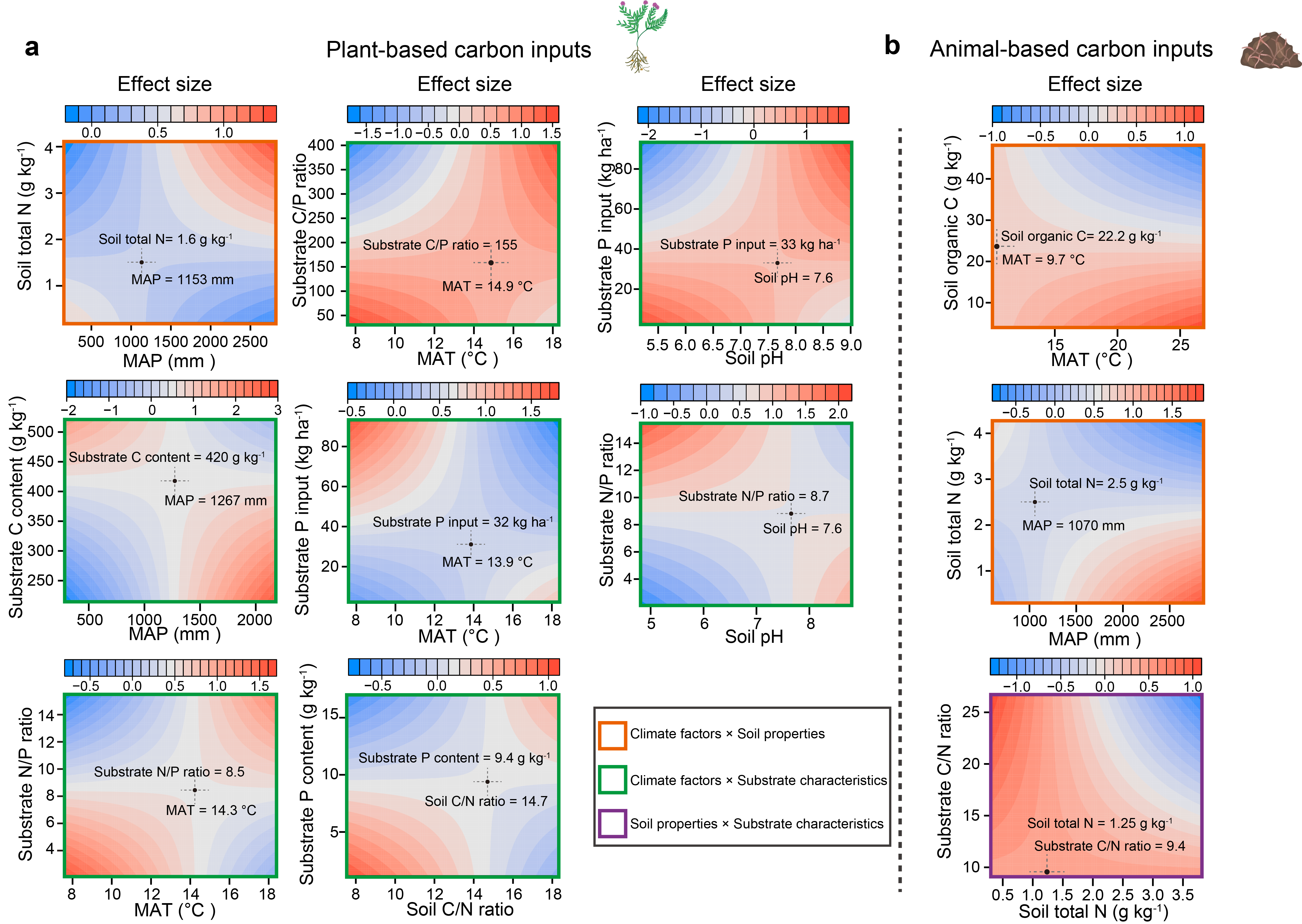


**Supplementary Fig. 5.** **Soil biota abundance responses to interactions between environmental variables and substrate characteristics.** Filled contour plots show soil biota abundance responses to interactions among climate factors, soil properties and substrate characteristics. The vertical and horizontal axes show the two interacting variables. Blue and red shading indicate low and high effect sizes, respectively, for plant-based carbon inputs (legume cover crops, a) and animal-based carbon inputs (vermicompost, b). Black dots mark transition points where the soil biota abundance response changes along the interaction gradient. Only significant interactions are shown (*P* < 0.05). Abbreviation: MAT, mean annual temperature; MAP, mean annual precipitation. All tested interactions are provided in Tables S6-S7.


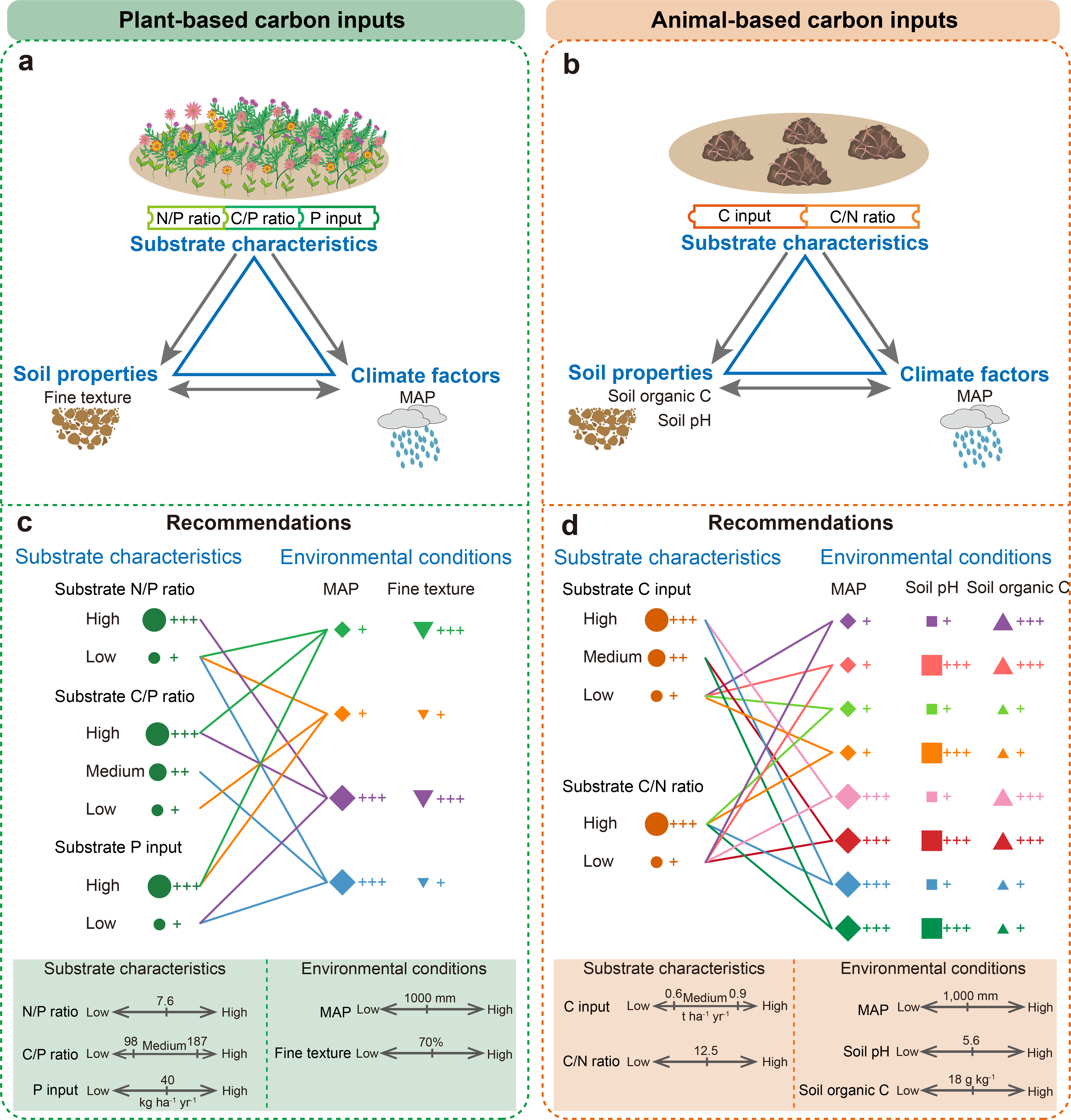


**Supplementary Fig. 6. Conceptual framework for optimizing carbon farming** **strategy under different environmental conditions.** The framework involves three steps: (i) diagnosing local climate and soil conditions (for example, MAP, soil texture, pH, and soil organic C), (ii) identifying the dominant environmental constraints within the local climate–soil system, and (iii) selecting plant- or animal-based carbon inputs by matching substrate characteristics to those constraints. For plant-based carbon sources (a, c), substrate N/P ratio, C/P ratio, and P input are matched with local MAP and fine texture to optimize soil biota abundance and thereby enhance agroecosystem multifunctionality. For example, plant-based carbon sources with lower N/P and C/P ratios and higher P input were more favorable in arid or semi-arid regions with coarse-textured soils (c). For animal-based carbon sources (b, d), substrate C input and C/N ratio are matched with MAP, soil pH, and soil organic C to optimize soil biota abundance and thereby enhance agroecosystem multifunctionality. Abbreviations: MAP, mean annual precipitation; fine texture, percentage of clay plus silt.


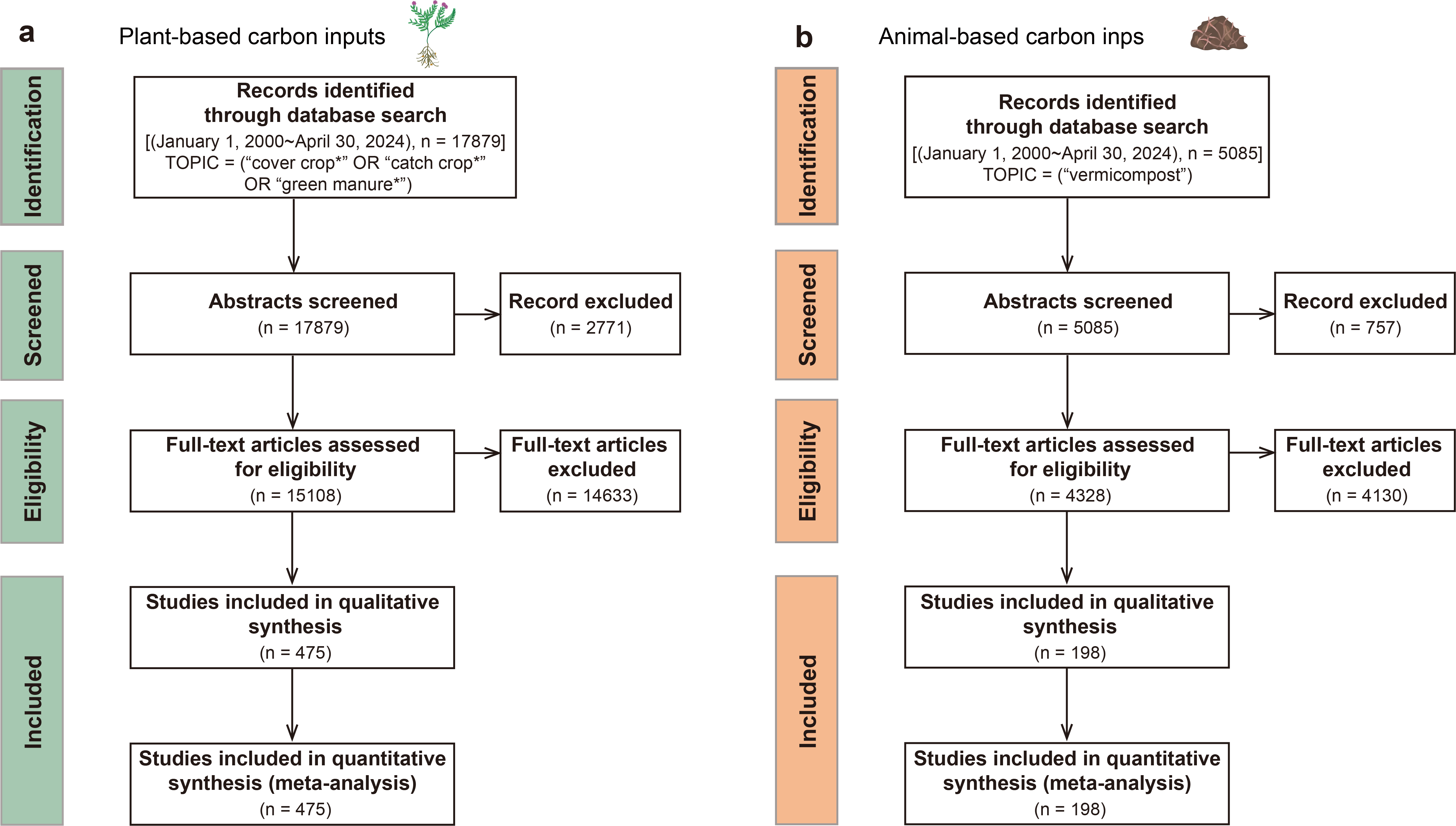


**Supplementary Fig. 7.** **PRISMA flow diagram showing the literature search and study screening procedure for the meta-analysis of agroecosystem multifunctionality under plant- and animal-based carbon inputs.**

**
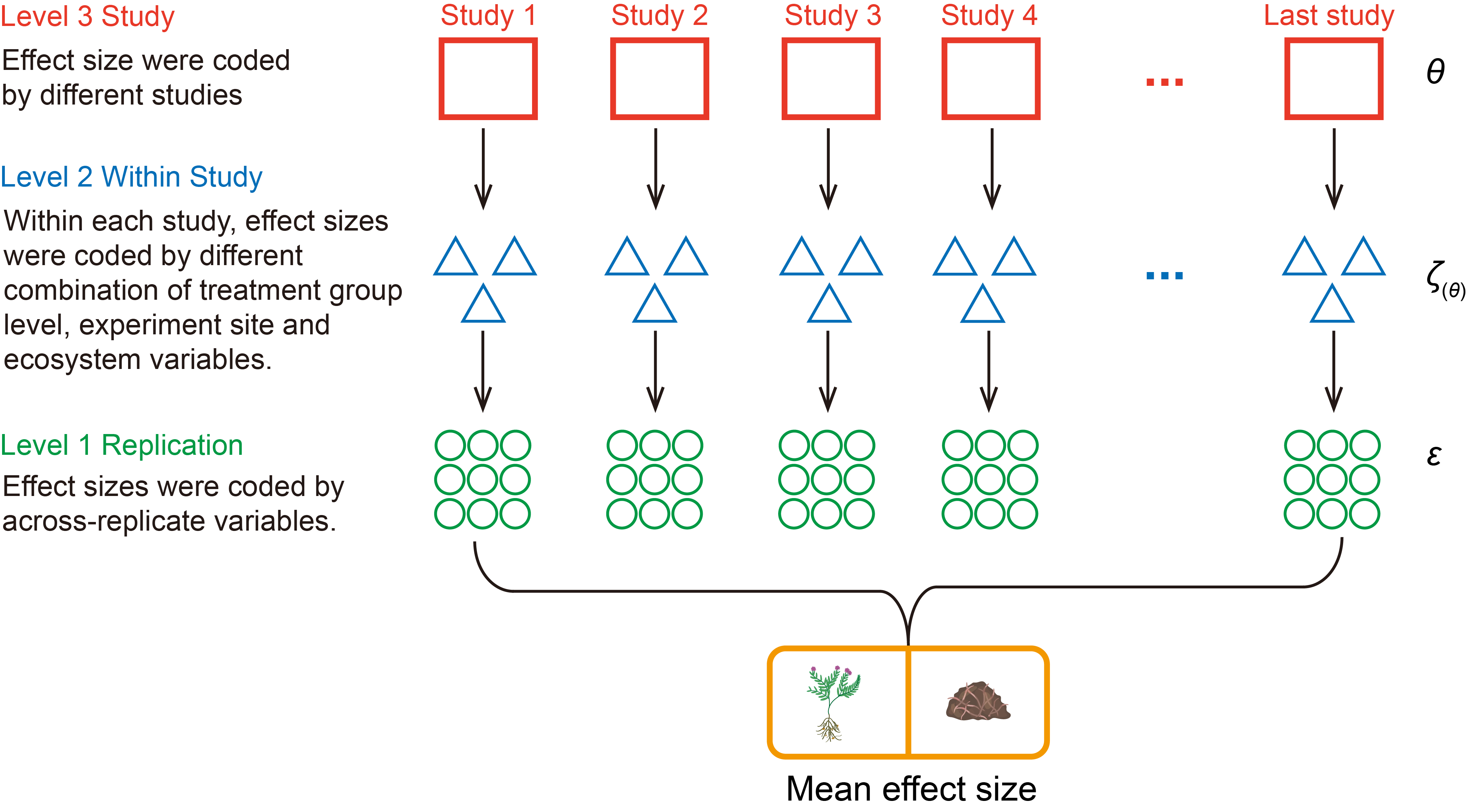
**

**Supplementary Fig. 8. Graphical illustration of the dataset structure and three-level hierarchical model.**

**
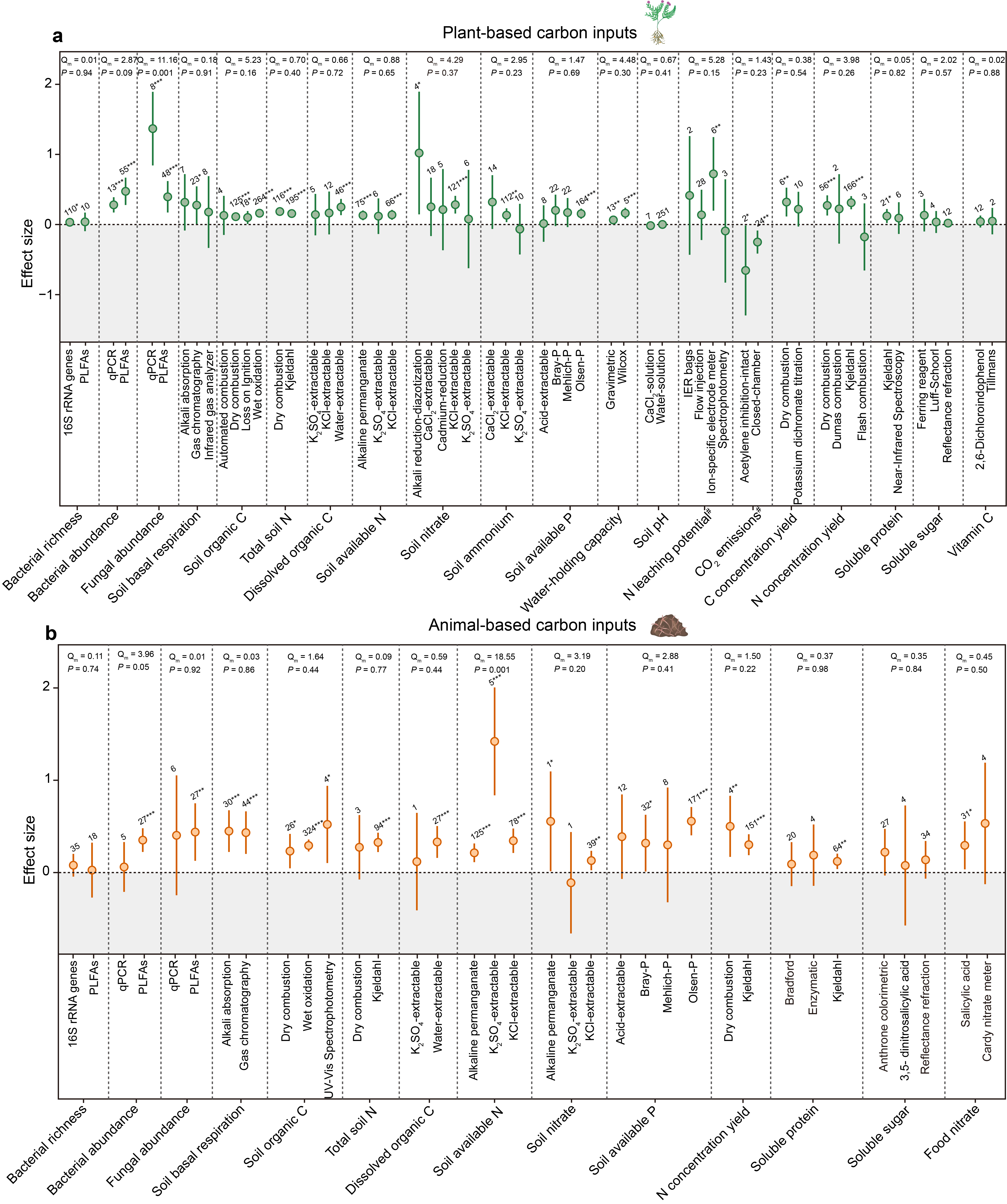
**

**Supplementary Fig. 9. Comparison of agroecosystem variable responses to plant- and animal-based carbon inputs across different measurement methods.** Mean effect sizes of plant-based carbon inputs (legume cover crops, a) and animal-based carbon inputs (vermicompost, b) for agroecosystem variables, shown with 95% confidence intervals (CIs). Numbers indicate sample sizes. Mean effect sizes were considered significant when the 95% CIs did not overlap zero (**P* < 0.05, ***P* < 0.01, and ****P* < 0.001). Method effects were tested using the omnibus test (Q_m_).

**
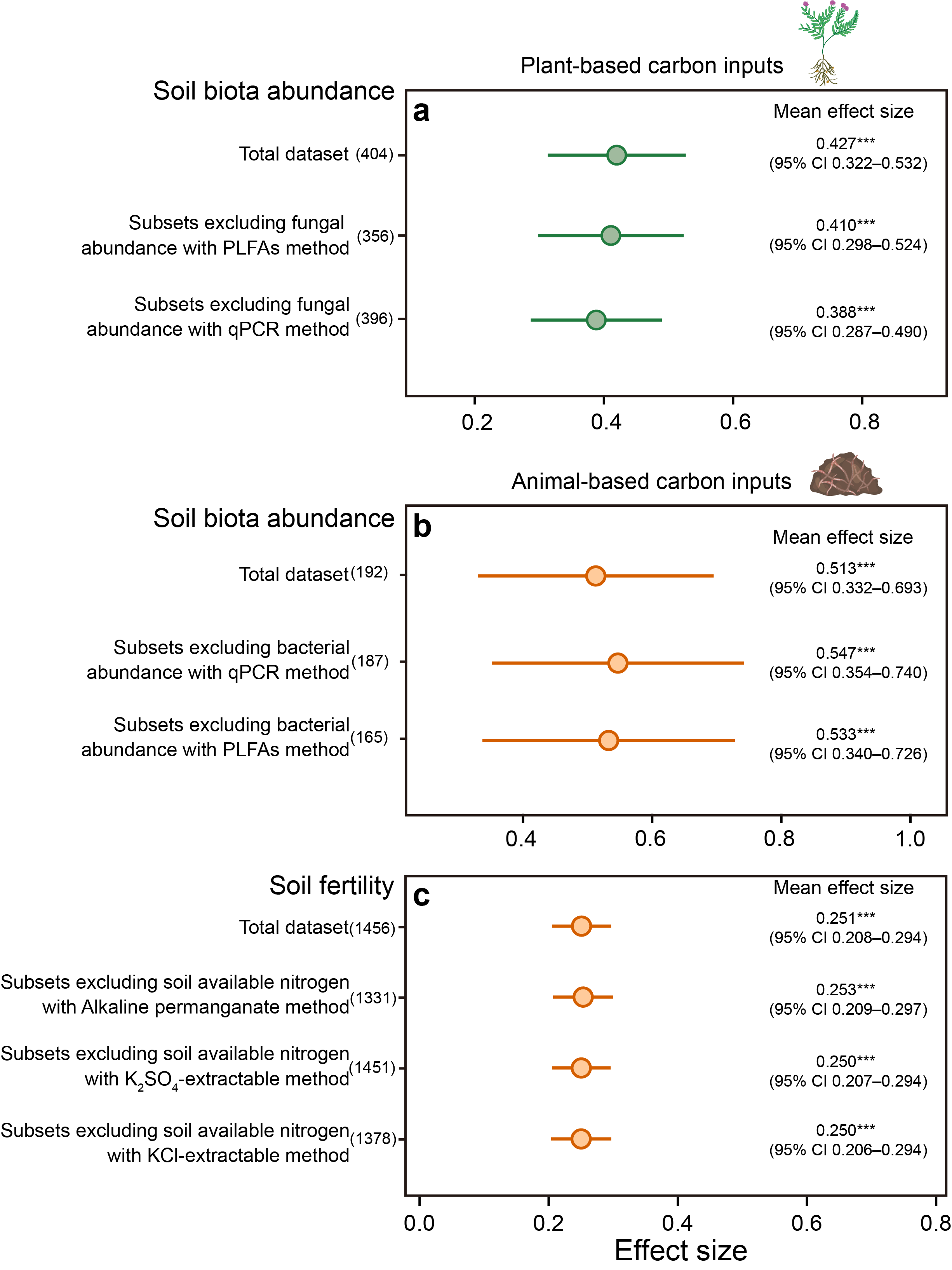
**

**Supplementary Fig. 10. Alternative analytical treatments did not change the responses of key agroecosystem functions to plant- and animal-based carbon inputs.** (a-b) Soil biota abundance under plant-based inputs (legume cover crops, a) and animal-based inputs (vermicompost, b), based on the full dataset and subsets excluding microbial abundance measured by qPCR or PLFA. (c) Soil fertility under animal-based carbon inputs (vermicompost), based on the full dataset and subsets excluding soil available nitrogen measured by alkaline permanganate, K_2_SO_4_-extractable, or KCl-extractable. Mean effect sizes were significantly different when the 95% CIs did not overlap zero (****P* < 0.001). Values in parentheses indicate sample sizes.

**Supplementary Tables**

**Supplementary Table 1.** List of ecosystem variables included in the meta-analysis. ^#^, values multiplied by -1 to ensure directional consistency with other ecosystem variables, with higher values indicating a more desirable state.

| **Ecosystem function proxies** | **Ecosystem variable** | **Method and Unit** |
| --- | --- | --- |
| **Soil biota richness** | Bacterial richness | Bacterial richness was measured using 16S rRNA genes and PLFA analysis methods. |
|  | Fungal richness | Fungal richness was measured using ITS genes and PLFAs methods. |
|  | Nematode richness | Nematode richness was measured using manual counting method. |
|  | Earthworm richness | Earthworm richness was measured using manual counting method. |
| **Soil biota abundance** | Bacterial abundance | Bacterial abundance was measured using qPCR and PLFAs methods, with the units reported as copies g^-1^ dry soil and nmol PLFA g^-1^ dry soil, respectively. |
|  | Fungal abundance | Fungal abundance was measured using qPCR and PLFAs methods, with the units reported as copies g^-1^ dry soil and nmol PLFA g^-1^ dry soil, respectively. |
|  | Bacterivorous nematodes | Bacterivorous nematodes abundance was measured using manual counting method, with the units reported as individuals 100 g^-1^ dry soil. |
|  | Fungivorous nematodes | Fungivorous nematodes abundance was measured using manual counting method, with the units reported as individuals 100 g^-1^ dry soil. |
|  | Herbivorous nematodes | Herbivorous nematodes abundance was measured using manual counting method, with the units reported as individuals 100 g^-1^ dry soil. |
|  | Omnivorous nematodes | Omnivorous nematodes abundance was measured using manual counting method, with the units reported as individuals 100 g^-1^ dry soil. |
|  | Predatory nematodes | Predatory nematodes abundance was measured using manual counting method, with the units reported as individuals 100 g^-1^ dry soil. |
|  | Total nematodes | Total nematodes abundance was measured using manual counting method, with the units reported as individuals 100 g^-1^ dry soil. |
|  | Earthworm abundance | Earthworm abundance was measured using manual counting method, while the unit was individuals m^-2^. |
| **Soil activity** | Microbial biomass C | Microbial biomass C was measured using the chloroform fumigation-extraction method, with the units reported as mg MBC kg^-1^ dry soil. |
|  | Microbial biomass N | Microbial biomass N was measured using the chloroform fumigation-extraction method, with the units reported as mg MBN kg^-1^ dry soil. |
|  | Soil basal respiration | Soil basal respiration was measured using Alkali absorption, gas chromatography, and infrared gas analyzer, with the units reported as the CO_2_ emission per gram of soil within a unit of time (i.e., mg CO_2_-C g^-1^ soil h^-1^, mg CO_2_-C g^-1^ soil day^-1^). |
|  | α-1,4-glucosidase  (C-cycle enzyme, AG) | AG was measured using p-nitrophenyl method, with the unit reported as µg pNP g^-1^ soil h^-1^. |
|  | β-1,4-glucosidase  (C-cycle enzyme, BG) | BG was measured using p-nitrophenyl method, with the unit reported as µg pNP g^-1^ soil h^-1^. |
|  | β-d-cellobiohydrolase  (C-cycle enzyme, CB) | CB was measured using p-nitrophenyl method, with the unit reported as µg pNP g^-1^ soil h^-1^. |
|  | β-xylosidase  (C-cycle enzyme, XYL) | XYL was measured using p-nitrophenyl method, with the unit reported as µg pNP g^-1^ soil h^-1^. |
|  | L-leucine aminopeptidase  (N-cycle enzyme, LAP) | LAP was measured using p-nitrophenyl method, with the unit reported as µg pNP g^-1^ soil h^-1^. |
|  | β-1,4-N-acetylglucosaminidase  (N-cycle enzyme, NAG) | NAG was measured using p-nitrophenyl method, with the unit reported as µg pNP g^-1^ soil h^-1^. |
|  | Phosphate  (P-cycle enzyme, PHOS) | PHOS was measured using p-nitrophenyl method, with the unit reported as µg pNP g^-1^ soil h^-1^. |
| **Soil fertility** | Soil organic C | Soil organic C was measured using wet oxidation, dry-combustion, loss on ignition, automated combustion technique, and UV-Vis Spectrophotometry methods, with the unit reported as g C kg^-1^ dry soil. Soil organic carbon data initially reported in stocks (in grams per square meter) were transformed to concentration (in per kilogram dry soil) using soil bulk density. Studies without reported bulk density and for which conversion was not possible were excluded. |
|  | Total soil N | Total soil N was measured using dry-combustion and Kjeldahl methods, with the unit reported as g N kg^-1^ dry soil. |
|  | Total soil P | Total soil P was measured using acid digestion, with the unit reported as g P kg^-1^ dry soil. |
|  | Dissolved organic C | Dissolved organic C was measured using K_2_SO_4_-extractable, KCl-extractable, and water-extractable methods, with the unit reported as mg C kg^-1^ dry soil. |
|  | Soil available N | Soil available N was measured using alkaline hydrolysis diffusion method, H3A-extractable, K_2_SO_4_-extractable, KCl-extractable, and alkaline permanganate, with the unit reported as mg N kg^-1^ dry soil. |
|  | Soil nitrate | Soil nitrate was measured using alkali reduction-diazotization, alkaline permanganate, CaCl_2_-extractable, cadmium-reduction, K_2_SO_4_-extractable, and KCl-extractable, with the unit reported as mg NO_3_-N kg^-1^ dry soil. |
|  | Soil ammonium | Soil ammonium was measured using CaCl_2_-extractable, K_2_SO_4_-extractable, and KCl-extractable, with the unit reported as mg NH_4_-N kg^-1^ dry soil. |
|  | Soil available P | Soil available phosphorus was measured using acid-extractable, Bray-P, Mehlich-P, and Olsen-P, with the unit reported as mg P kg^-1^ dry soil. |
|  | Soil moisture | Soil moisture (in %) was measured using gravimetric method. |
|  | Water-holding capacity | Water-holding capacity (in %)—the amount of water that a given soil can retain—was measured using the gravimetric and Wilcox method. |
|  | Soil pH | Soil pH was measured using pH meter in different solutions (for example, H_2_O or CaCl_2_). |
|  | Soil EC | Soil EC (in us cm^-1^) was determined using a conductivity meter in water solutions. |
| **Soil physical structure** | Soil bulk density^#^ | Soil bulk density (in g cm^-3^) was measured using the core sample method. |
| **Water regulation** | N leaching potential^#^ | N leaching potential represents the cumulative loss of nitrogen per unit area during the main crop growth period, and it was measured using flow injection analyzer, ion-exchange resin (IER) bags, ion-specific electrode meter, and spectrophotometry, with the unit reported as kg N ha^-1^ per growth period. |
|  | P leaching potential^#^ | P leaching potential represents the cumulative loss of P per unit area during the main crop growth period, and it was measured using flow injection analyzer, with the unit is kg P ha^-1^ per growth period. |
| **Climate regulation** | CH_4_ emissions^#^ | CH_4_ emissions represented the cumulative emissions of CH_4_ per unit area during the main crop growth period, and it was measured using the static chamber method, with the unit reported as kg C-CH_4_ ha^-1^ per growth period. |
|  | CO_2_ emissions^#^ | CO_2_ emissions represented the cumulative emissions of CO_2_ per unit area during the main crop growth period, and it was measured using the acetylene inhibition-intact and static chamber method, with the unit reported as kg C-CO_2_ ha^-1^ per growth period. |
|  | N_2_O emissions^#^ | N_2_O emissions represented the cumulative emissions of N_2_O per unit area during the main crop growth period, and it was measured using the static chamber method, with the unit reported as kg N-N_2_O ha^-1^ per growth period. |
| **Plant productivity** | Crop yield | Crop yield (t ha^-1^) was a metric of the quantity of harvested crop production per land area, and was measured using biomass determination methods. |
|  | Aboveground biomass | Aboveground biomass (t ha^-1^) was a metric of the quantity of crop aboveground production per land area, and was measured using biomass determination methods. |
|  | Belowground biomass | Belowground biomass (t ha^-1^) was a metric of the quantity of crop belowground production per land area, and was measured using biomass determination methods. |
| **Product quality** | C concentration yield | C concentration yield (in kg C ha^-1^) was defined as the harvested crop C, and calculated as the grain C content multiplied by grain yield. It was measured using dry-combustion and potassium dichromate titration. |
|  | N concentration yield | N concentration yield (in kg N ha^-1^) is defined as the harvested crop N, and calculated as the grain N content multiplied by grain yield. It was measured using dry-combustion, Dumas combustion, flash combustion, and Kjeldahl methods. |
|  | P concentration yield | P concentration yield (in kg P ha^-1^) is defined as the harvested crop P, and calculated as the grain P content multiplied by grain yield. It was measured using the acid digestion method. |
|  | Soluble protein | Soluble protein was measured using Kjeldahl, Near-Infrared Spectroscopy, enzymatic hydrolysis, and Bradford methods, with the unit reported as mg g^-1^. |
|  | Soluble sugar | Soluble sugar (%) was measured using Luff-Schoorl, anthrone colorimetric, 3,5-dinitrosalicylic acid, Ferring Reagent, and reflectance refraction methods. |
|  | Vitamin C | Vitamin C (mg 100 g^-1^) was measured using the 2,6-dichloroindophenol and Tillman's methods. |
|  | Organic acid | Organic acid (%) was measured using acid-base titration. |
|  | Sugar-acid ratio | Sugar-acid ratio (%) represents the ratio of soluble sugar to organic acid. |
|  | Fruit nitrate^#^ | Fruit nitrate (mg g^-1^) was measured using the salicylic acid method and Cardy nitrate meter method. |

**Supplementary Table 2.** Effect size estimates and model-based sensitivity analysis for the plant-based carbon inputs using multilevel and simple random-effects models. Mean effect sizes (ESs) with 95% confidence intervals (CIs) are provided. Publication bias in the simple random-effects models was assessed using the fail-safe number, Egger’s regression test, and the trim and fill method.

| **Category** | **n** | **Multilevel random-effect model** | | | **Fail-safe number** | **Egger's z** | **Simple random-effect model** | |  | **Trim and fill model^#^** | | |
| --- | --- | --- | --- | --- | --- | --- | --- | --- | --- | --- | --- | --- |
|  |  | **ESs** | **95% CIs** | **Changes %** |  |  | **ESs** | **95% CIs** |  | **ESs** | **95% CIs** | **n_T_** |
| **Overall (Multifunctionality)** | 4745 | 0.160 | (0.137, 0.183) | 17.35 | 183255266 | 4.753* | 0.156 | (0.145, 0.167) |  | 0.156 | (0.145, 0.167) | 4745 |
| **Functions** |  |  |  |  |  |  |  |  |  |  |  |  |
| Soil biota richness | 226 | 0.028 | (-0.007, 0.063) | 2.83 | 11199 | 0.490 | 0.027 | (0.005, 0.049) |  | 0.027 | (0.005, 0.049) | 226 |
| Soil biota abundance | 404 | 0.427 | (0.322, 0.532) | 53.27 | 2906312 | 3.955* | 0.362 | (0.298, 0.426) |  | 0.362 | (0.298, 0.426) | 404 |
| Soil activity | 538 | 0.335 | (0.266, 0.403) | 39.74 | 5375469 | -0.988 | 0.274 | (0.240, 0.307) |  | 0.405 | (0.370, 0.440) | 658 |
| Soil fertility | 1896 | 0.135 | (0.116, 0.154) | 14.45 | 23269795 | 2.005* | 0.131 | (0.119, 0.143) |  | 0.131 | (0.119, 0.143) | 1896 |
| Soil physical structure | 115 | 0.045 | (0.027, 0.062) | 4.55 | 36816 | 1.734 | 0.044 | (0.033, 0.054) |  | 0.044 | (0.033, 0.054) | 115 |
| Water regulation | 41 | 0.314 | (0.110, 0.517) | 36.88 | 5540 | -0.262 | 0.296 | (0.123, 0.469) |  | 0.478 | (0.297, 0.659) | 50 |
| Climate regulation | 138 | -0.259 | (-0.403, -0.115) | -22.79 | 2664195 | -0.261 | -0.345 | (-0.447, -0.243) |  | -0.345 | (-0.447, -0.243) | 138 |
| Plant production | 1006 | 0.151 | (0.117, 0.185) | 16.26 | 12834360 | 1.263 | 0.145 | (0.125, 0.165) |  | 0.145 | (0.125, 0.165) | 1006 |
| Product quality | 381 | 0.239 | (0.178, 0.299) | 26.95 | 5595779 | -1.130 | 0.210 | (0.182, 0.239) |  | 0.310 | (0.281, 0.339) | 481 |
| **Services** |  |  |  |  |  |  |  |  |  |  |  |  |
| Supporting | 3179 | 0.182 | (0.157, 0.207) | 19.96 | 83658277 | 6.707* | 0.172 | (0.160, 0.185) |  | 0.172 | (0.160, 0.185) | 3179 |
| Regulating | 179 | -0.126 | (-0.268, 0.016) | -11.83 | 2425864 | 1.684 | -0.201 | (-0.297, -0.105) |  | -0.396 | (-0.500, -0.292) | 212 |
| Provisioning | 1387 | 0.162 | (0.129, 0.196) | 17.63 | 35380508 | 0.162 | 0.163 | (0.146, 0.179) |  | 0.163 | (0.146, 0.179) | 1387 |

* denotes *P* < 0.05, which suggests publication bias.

^#^ Trim and fill model does not run for hierarchical models in ‘*metafor*’ package of R (rma.mv), they were based on random model without hierarchical structure (rma).

n_T_: number of imputed studies estimated using the trim and fill method.

**Supplementary Table 3.** Effect size estimates and model-based sensitivity analysis for the animal-based carbon inputs using multilevel and simple random-effects models. Mean effect sizes (ESs) with 95% confidence intervals (CIs) are provided. Publication bias in the simple random-effects models was assessed using the fail-safe number, Egger’s regression test, and the trim and fill method.

| **Category** | **n** | **Multilevel random-effect model** | | | **Fail-safe number** | **Egger's z** | **Simple random-effect model** | |  | **Trim and fill model^#^** | | |
| --- | --- | --- | --- | --- | --- | --- | --- | --- | --- | --- | --- | --- |
|  |  | **ESs** | **95% CIs** | **Changes %** |  |  | **ESs** | **95% CIs** |  | **ESs** | **95% CIs** | **n_T_** |
| **Overall (Multifunctionality)** | 3764 | 0.270 | (0.237, 0.303) | 30.98 | 353933177 | 9.516* | 0.267 | (0.253, 0.280) |  | 0.267 | (0.253, 0.28) | 3764 |
| **Functions** |  |  |  |  |  |  |  |  |  |  |  |  |
| Soil biota richness | 96 | 0.122 | (0.017, 0.227) | 12.98 | 18504 | 0.776 | 0.113 | (0.065, 0.162) |  | 0.113 | (0.065, 0.162) | 96 |
| Soil biota abundance | 192 | 0.513 | (0.332, 0.693) | 66.96 | 831891 | -0.148 | 0.488 | (0.397, 0.579) |  | 0.488 | (0.397, 0.579) | 192 |
| Soil activity | 461 | 0.394 | (0.313, 0.474) | 48.23 | 9593718 | 4.115* | 0.425 | (0.385, 0.466) |  | 0.425 | (0.385, 0.466) | 461 |
| Soil fertility | 1456 | 0.251 | (0.208, 0.294) | 28.51 | 52883564 | 5.110* | 0.234 | (0.214, 0.253) |  | 0.234 | (0.214, 0.253) | 1456 |
| Soil physical structure | 155 | 0.070 | (0.057, 0.082) | 7.20 | 450318 | 2.520* | 0.070 | (0.061, 0.079) |  | 0.070 | (0.061, 0.079) | 155 |
| Water regulation | 24 | -0.347 | (-1.219, 0.525) | -29.31 | 17466 | 3.896* | -0.481 | (-0.783, -0.180) |  | -0.555 | (-0.851, -0.26) | 26 |
| Climate regulation | 63 | -0.202 | (-0.375, -0.029) | -18.28 | 14559 | -0.384 | -0.204 | (-0.274, -0.134) |  | -0.204 | (-0.274, -0.134) | 63 |
| Plant production | 689 | 0.327 | (0.265, 0.389) | 38.65 | 12599478 | 5.511* | 0.303 | (0.272, 0.333) |  | 0.303 | (0.272, 0.333) | 689 |
| Product quality | 628 | 0.255 | (0.190, 0.320) | 29.02 | 11745869 | 2.728* | 0.272 | (0.24, 0.303) |  | 0.272 | (0.240, 0.303) | 628 |
| **Services** |  |  |  |  |  |  |  |  |  |  |  |  |
| Supporting | 2360 | 0.276 | (0.240, 0.312) | 31.76 | 146151052 | 9.395* | 0.275 | (0.258, 0.291) |  | 0.275 | (0.258, 0.291) | 2360 |
| Regulating | 87 | -0.249 | (-0.417, -0.081) | -22.00 | 64008 | 2.382* | -0.281 | (-0.381, -0.181) |  | -0.431 | (-0.528, -0.334) | 110 |
| Provisioning | 1317 | 0.312 | (0.258, 0.367) | 36.63 | 48677038 | 5.749* | 0.288 | (0.266, 0.310) |  | 0.288 | (0.266, 0.31) | 1317 |

* denotes *P* < 0.05, which suggests publication bias.

^#^ Trim and Fill model does not run for hierarchical models in ‘*metafor*’ package of R (rma.mv), they were based on random model without hierarchical structure (rma).

n_T_: number of imputed studies estimated using the trim and fill method.

**Supplementary Table 4.** Proportions of pairwise functional outcomes (win-win, trade-off, and lose-lose) between soil biota abundance or richness and individual ecosystem functions under plant- and animal-based carbon inputs. Values show the percentage (%) of paired observations classified into each outcome category based on the direction of effect sizes (ln*RR*).

| **Outcome type*** | **Soil biota richness** | **Soil activity** | **Soil fertility** | **Soil physical structure** | **Water regulation** | **Climate regulation** | **Plant production** | **Product quality** |
| --- | --- | --- | --- | --- | --- | --- | --- | --- |
| **Plant-based carbon inputs — Soil biota abundance** | | | | | | | | |
| Win-win (+/+) | 47.06 | 82.22 | 73.12 | 71.43 | 100.00 | 50.00 | 72.22 | 92.86 |
| Trade-off1 (−/+) | 1.96 | 2.22 | 10.75 | 0.00 | 0.00 | 0.00 | 12.50 | 7.14 |
| Trade-off2 (+/−) | 37.25 | 15.56 | 11.83 | 25.00 | 0.00 | 50.00 | 11.11 | 0.00 |
| Lose-lose (−/−) | 13.73 | 0.00 | 4.30 | 3.57 | 0.00 | 0.00 | 4.17 | 0.00 |
| **Plant-based carbon inputs — Soil biota richness** | | | | | | | | |
| Win-win (+/+) | 47.06 | 54.39 | 50.94 | 37.04 | 50.00 | 50 | 43.10 | 53.33 |
| Trade-off1 (−/+) | 37.26 | 31.58 | 33.96 | 59.26 | 50.00 | 25 | 44.83 | 40.00 |
| Trade-off2 (+/−) | 1.96 | 7.02 | 4.72 | 0.00 | 0.00 | 25 | 6.90 | 0.00 |
| Lose-lose (−/−) | 13.73 | 7.02 | 10.38 | 3.70 | 0.00 | 0 | 5.17 | 6.67 |
| **Animal-based carbon inputs — Soil biota abundance** | | | | | | | | |
| Win-win (+/+) | 63.89 | 100.00 | 82.93 | 100.00 | 37.50 | 66.67 | 91.89 | 100.00 |
| Trade-off1 (−/+) | 11.11 | 0.00 | 0.00 | 0.00 | 12.50 | 0.00 | 5.41 | 0.00 |
| Trade-off2 (+/−) | 25.00 | 0.00 | 17.07 | 0.00 | 50.00 | 33.33 | 2.70 | 0.00 |
| Lose-lose (−/−) | 0.00 | 0.00 | 0.00 | 0.00 | 0.00 | 0.00 | 0.00 | 0.00 |
| **Animal-based carbon inputs — Soil biota richness** | | | | | | | | |
| Win-win (+/+) | 63.89 | 66.67 | 76.74 | 62.50 | 25.00 | 0.00 | 82.14 | 66.67 |
| Trade-off1 (−/+) | 25.00 | 18.52 | 20.93 | 37.50 | 25.00 | 50.00 | 17.86 | 33.33 |
| Trade-off2 (+/−) | 11.11 | 14.81 | 0.00 | 0.00 | 50.00 | 50.00 | 0.00 | 0.00 |
| Lose-lose (−/−) | 0.00 | 0.00 | 2.33 | 0.00 | 0.00 | 0.00 | 0.00 | 0.00 |

* Win-win (+/+) indicates simultaneous positive responses of both variables, whereas lose-lose (−/−) indicates simultaneous negative responses. Trade-offs represent cases where one variable increases while the other decreases, with (+/−) indicating positive effects on soil biota attributes and negative effects on ecosystem functions, and (−/+) indicating the opposite pattern.

**Supplementary Table 5.** Summary of site and environmental characteristics for plant- and animal-based carbon inputs included in the meta-analysis.

| **Factors** | **Details** | **Observed range**  **(legume; vermicompost)** |
| --- | --- | --- |
| **Climate factors** |  |  |
| MAT | mean annual temperature (°C) | 1.92-26.53; 10.02-26.88 |
| MAP | mean annual precipitation (mm) | 150.00-2815.50; 602.23-2862.10 |
| **Soil properties** |  |  |
| Fine texture | Percentage of clay plus silt (%) | 4.80-92.00; 30.73-80.27 |
| Soil organic C | Soil organic carbon (g kg^-1^ dry soil) | 3.73-63.74; 4.00-48.13 |
| Soil total N | Soil total nitrogen (g kg^-1^ dry soil) | 0.20-4.11; 0.30-4.27 |
| Soil pH | Soil pH | 4.81-9.02; 3.89-9.02 |
| Soil C/N ratio | Soil C/N ratio | 5.75-26.57; 5.07-15.00 |
| **Substrate characteristics** |  |  |
| Substrate N content | N content of substrate (g kg^-1^) | 8.31-43.27; 6.42-21.00 |
| Substrate P content | P content of substrate (g kg^-1^) | 1.13-17.00; 2.68-57.60 |
| Substrate C content | C content of substrate (g kg^-1^) | 214.57-521.00; 93.60-464.90 |
| Substrate N input | Total N input from substrate (kg N ha^-1^ year^-1^) | 18.59-388.74; 14.63-309.00 |
| Substrate P input | Total P input from substrate (kg P ha^-1^ year^-1^) | 2.30-93.30; 12.10-475.28 |
| Substrate C input | Total C input from substrate (t C ha^-1^ year^-1^) | 0.22-6.49; 0.31-2.81 |
| Substrate C/N ratio | C/N ratio of substrate | 9.90-32.64; 9.09-26.72 |
| Substrate N/P ratio | N/P ratio of substrate | 2.06-15.47; 0.18-3.84 |
| Substrate C/P ratio | C/P ratio of substrate | 30.65-405.45; 3.60-39.81 |

**Supplementary Table 6.** Multilevel meta-analysis of soil biota abundance responses to moderator interactions (climate factors × soil properties, climate factors × substrate characteristics, and soil properties × substrate characteristics) under plant-based carbon inputs. Q_m_, test statistic for individual moderator effects derived from multilevel meta-analysis models. Abbreviations: MAT, mean annual temperature; MAP, mean annual precipitation; fine texture, percentage of clay plus silt.

| **Model** | **Main and interaction effect** | **Q_m_** | ***P* value** |
| --- | --- | --- | --- |
| **Climate factors × Soil properties** | | | |
| MAT * Fine texture | MAT | 0.449 | 0.503 |
|  | Fine texture | 0.179 | 0.672 |
|  | MAT:Fine texture | 0.622 | 0.430 |
| MAT * Soil organic C | MAT | 0.027 | 0.869 |
|  | Soil organic C | 0.105 | 0.745 |
|  | MAT:Soil organic C | 0.089 | 0.766 |
| MAT * Soil total N | MAT | 0.298 | 0.585 |
|  | Soil total N | 0.526 | 0.468 |
|  | MAT:Soil total N | 0.522 | 0.470 |
| MAT * Soil pH | MAT | 0.953 | 0.329 |
|  | Soil pH | 0.302 | 0.583 |
|  | MAT:Soil pH | 1.192 | 0.275 |
| MAT * Soil C/N ratio | MAT | 1.289 | 0.256 |
|  | Soil C/N ratio | 1.042 | 0.307 |
|  | MAT:Soil C/N ratio | 1.288 | 0.258 |
| MAP * Fine texture | MAP | 0.115 | 0.734 |
|  | Fine texture | 0.848 | 0.357 |
|  | MAP:Fine texture | 0.115 | 0.735 |
| MAP * Soil organic C | MAP | 2.132 | 0.144 |
|  | Soil organic C | 2.563 | 0.109 |
|  | MAP:Soil organic C | 2.793 | 0.095 |
| MAP * Soil total N | MAP | 3.437 | 0.064 |
|  | Soil total N | 3.877 | 0.049 |
|  | MAP:Soil total N | 4.297 | 0.038 |
| MAP * Soil pH | MAP | 0.753 | 0.386 |
|  | Soil pH | 1.771 | 0.183 |
|  | MAP:Soil pH | 0.631 | 0.427 |
| MAP * Soil C/N ratio | MAP | 1.781 | 0.182 |
|  | Soil C/N ratio | 1.363 | 0.243 |
|  | MAP:Soil C/N ratio | 2.052 | 0.152 |
| **Climate factors × Substrate characteristics** | | | |
| MAT * Substrate N content | MAT | 0.027 | 0.880 |
|  | Substrate N content | 0.001 | 0.975 |
|  | MAT:Substrate N content | 0.001 | 0.981 |
| MAT * Substrate P content | MAT | 1.920 | 0.166 |
|  | Substrate P content | 2.023 | 0.155 |
|  | MAT:Substrate P content | 2.610 | 0.106 |
| MAT * Substrate C content | MAT | 0.352 | 0.553 |
|  | Substrate C content | 0.329 | 0.566 |
|  | MAT:Substrate C content | 0.289 | 0.591 |
| MAT * Substrate N input | MAT | 0.201 | 0.654 |
|  | Substrate N input | 1.079 | 0.299 |
|  | MAT:Substrate N input | 0.820 | 0.365 |
| MAT * Substrate P input | MAT | 1.756 | 0.185 |
|  | Substrate P input | 6.761 | 0.009 |
|  | MAT:Substrate P input | 7.731 | 0.005 |
| MAT * Substrate C input | MAT | 1.495 | 0.222 |
|  | Substrate C input | 1.805 | 0.179 |
|  | MAT:Substrate C input | 1.943 | 0.163 |
| MAT * Substrate C/N ratio | MAT | 0.024 | 0.878 |
|  | Substrate C/N ratio | 0.002 | 0.962 |
|  | MAT:Substrate C/N ratio | 0.001 | 0.989 |
| MAT * Substrate N/P ratio | MAT | 15.186 | < 0.001 |
|  | Substrate N/P ratio | 5.965 | 0.015 |
|  | MAT:Substrate N/P ratio | 7.659 | 0.006 |
| MAT * Substrate C/P ratio | MAT | 8.531 | 0.004 |
|  | Substrate C/P ratio | 4.474 | 0.034 |
|  | MAT:Substrate C/P ratio | 4.958 | 0.026 |
| MAP * Substrate N content | MAP | 1.571 | 0.210 |
|  | Substrate N content | 1.395 | 0.238 |
|  | MAP:Substrate N content | 1.395 | 0.238 |
| MAP * Substrate P content | MAP | 0.763 | 0.382 |
|  | Substrate P content | 0.388 | 0.534 |
|  | MAP:Substrate P content | 0.234 | 0.628 |
| MAP * Substrate C content | MAP | 11.308 | < 0.001 |
|  | Substrate C content | 11.625 | < 0.001 |
|  | MAP:Substrate C content | 11.103 | < 0.001 |
| MAP * Substrate N input | MAP | 0.956 | 0.328 |
|  | Substrate N input | 1.852 | 0.174 |
|  | MAP:Substrate N input | 1.565 | 0.211 |
| MAP * Substrate P input | MAP | 6.545 | 0.011 |
|  | Substrate P input | 11.050 | < 0.001 |
|  | MAP:Substrate P input | 12.775 | < 0.001 |
| MAP * Substrate C input | MAP | 2.559 | 0.110 |
|  | Substrate C input | 2.844 | 0.092 |
|  | MAP:Substrate C input | 3.101 | 0.078 |
| MAP * Substrate C/N ratio | MAP | 0.308 | 0.579 |
|  | Substrate C/N ratio | 0.112 | 0.738 |
|  | MAP:Substrate C/N ratio | 0.212 | 0.646 |
| MAP * Substrate N/P ratio | MAP | 13.740 | < 0.001 |
|  | Substrate N/P ratio | 10.119 | 0.002 |
|  | MAP:Substrate N/P ratio | 15.513 | < 0.001 |
| MAP * Substrate C/P ratio | MAP | 10.245 | 0.001 |
|  | Substrate C/P ratio | 12.000 | < 0.001 |
|  | MAP:Substrate C/P ratio | 12.830 | < 0.001 |
| **Soil properties × Substrate characteristics** | | | |
| Fine texture * Substrate N content | Fine texture | 0.001 | 0.975 |
|  | Substrate N content | 0.008 | 0.929 |
|  | Fine texture:Substrate N content | 0.012 | 0.913 |
| Fine texture * Substrate P content | Fine texture | 0.016 | 0.905 |
|  | Substrate P content | 0.722 | 0.395 |
|  | Fine texture:Substrate P content | 0.943 | 0.331 |
| Fine texture * Substrate C content | Fine texture | 1.413 | 0.235 |
|  | Substrate C content | 1.467 | 0.226 |
|  | Fine texture:Substrate C content | 1.509 | 0.219 |
| Fine texture * Substrate N input | Fine texture | 0.457 | 0.499 |
|  | Substrate N input | 0.515 | 0.473 |
|  | Fine texture:Substrate N input | 0.419 | 0.518 |
| Fine texture * Substrate P input | Fine texture | 0.570 | 0.450 |
|  | Substrate P input | 2.765 | 0.096 |
|  | Fine texture:Substrate P input | 2.778 | 0.096 |
| Fine texture * Substrate C input | Fine texture | 0.279 | 0.597 |
|  | Substrate C input | 0.162 | 0.687 |
|  | Fine texture:Substrate C input | 0.182 | 0.670 |
| Fine texture * Substrate C/N ratio | Fine texture | 0.039 | 0.843 |
|  | Substrate C/N ratio | 0.397 | 0.529 |
|  | Fine texture:Substrate C/N ratio | 0.287 | 0.592 |
| Fine texture * Substrate N/P ratio | Fine texture | 2.470 | 0.116 |
|  | Substrate N/P ratio | 0.664 | 0.415 |
|  | Fine texture:Substrate N/P ratio | 0.776 | 0.378 |
| Fine texture * Substrate C/P ratio | Fine texture | 15.034 | < 0.001 |
|  | Substrate C/P ratio | 13.596 | < 0.001 |
|  | Fine texture:Substrate C/P ratio | 14.331 | < 0.001 |
| Soil organic C * Substrate N content | Soil organic C | 0.001 | 0.971 |
|  | Substrate N content | 0.121 | 0.728 |
|  | Soil organic C:Substrate N content | 0.245 | 0.621 |
| Soil organic C * Substrate P content | Soil organic C | 0.660 | 0.417 |
|  | Substrate P content | 0.697 | 0.404 |
|  | Soil organic C:Substrate P content | 1.565 | 0.211 |
| Soil organic C * Substrate C content | Soil organic C | 0.658 | 0.417 |
|  | Substrate C content | 0.064 | 0.800 |
|  | Soil organic C:Substrate C content | 0.217 | 0.642 |
| Soil organic C * Substrate N input | Soil organic C | 0.545 | 0.461 |
|  | Substrate N input | 0.426 | 0.514 |
|  | Soil organic C:Substrate N input | 0.081 | 0.776 |
| Soil organic C * Substrate P input | Soil organic C | 0.660 | 0.417 |
|  | Substrate P input | 1.019 | 0.313 |
|  | Soil organic C:Substrate P input | 0.982 | 0.322 |
| Soil organic C * Substrate C input | Soil organic C | 1.529 | 0.216 |
|  | Substrate C input | 0.029 | 0.865 |
|  | Soil organic C:Substrate C input | 0.079 | 0.778 |
| Soil organic C * Substrate C/N ratio | Soil organic C | 2.090 | 0.148 |
|  | Substrate C/N ratio | 1.295 | 0.255 |
|  | Soil organic C:Substrate C/N ratio | 1.094 | 0.296 |
| Soil organic C * Substrate N/P ratio | Soil organic C | 0.002 | 0.965 |
|  | Substrate N/P ratio | 0.107 | 0.744 |
|  | Soil organic C:Substrate N/P ratio | 0.004 | 0.947 |
| Soil organic C * Substrate C/P ratio | Soil organic C | 1.560 | 0.212 |
|  | Substrate C/P ratio | 1.224 | 0.269 |
|  | Soil organic C:Substrate C/P ratio | 1.366 | 0.243 |
| Soil total N * Substrate N content | Soil total N | 0.048 | 0.827 |
|  | Substrate N content | 0.194 | 0.660 |
|  | Soil total N:Substrate N content | 0.416 | 0.519 |
| Soil total N * Substrate P content | Soil total N | 0.279 | 0.597 |
|  | Substrate P content | 0.269 | 0.604 |
|  | Soil total N:Substrate P content | 1.781 | 0.182 |
| Soil total N * Substrate C content | Soil total N | 0.579 | 0.447 |
|  | Substrate C content | 0.117 | 0.734 |
|  | Soil total N:Substrate C content | 0.287 | 0.592 |
| Soil total N * Substrate N input | Soil total N | 0.089 | 0.767 |
|  | Substrate N input | 0.507 | 0.477 |
|  | Soil total N:Substrate N input | 0.188 | 0.664 |
| Soil total N * Substrate P input | Soil total N | 0.029 | 0.865 |
|  | Substrate P input | 0.001 | 0.976 |
|  | Soil total N:Substrate P input | 0.001 | 0.987 |
| Soil total N * Substrate C input | Soil total N | 0.595 | 0.441 |
|  | Substrate C input | 0.001 | 0.973 |
|  | Soil total N:Substrate C input | 0.007 | 0.932 |
| Soil total N * Substrate C/N ratio | Soil total N | 2.204 | 0.138 |
|  | Substrate C/N ratio | 1.596 | 0.207 |
|  | Soil total N:Substrate C/N ratio | 1.470 | 0.225 |
| Soil total N * Substrate N/P ratio | Soil total N | 0.186 | 0.666 |
|  | Substrate N/P ratio | 0.003 | 0.959 |
|  | Soil total N:Substrate N/P ratio | 0.081 | 0.777 |
| Soil total N * Substrate C/P ratio | Soil total N | 0.001 | 0.972 |
|  | Substrate C/P ratio | 0.068 | 0.795 |
|  | Soil total N:Substrate C/P ratio | 0.022 | 0.883 |
| Soil pH * Substrate N content | Soil pH | 0.369 | 0.544 |
|  | Substrate N content | 0.513 | 0.474 |
|  | Soil pH:Substrate N content | 0.528 | 0.467 |
| Soil pH * Substrate P content | Soil pH | 2.598 | 0.107 |
|  | Substrate P content | 2.098 | 0.148 |
|  | Soil pH:Substrate P content | 1.966 | 0.161 |
| Soil pH * Substrate C content | Soil pH | 0.985 | 0.321 |
|  | Substrate C content | 1.142 | 0.285 |
|  | Soil pH:Substrate C content | 1.331 | 0.249 |
| Soil pH * Substrate N input | Soil pH | 0.273 | 0.602 |
|  | Substrate N input | 0.002 | 0.966 |
|  | Soil pH:Substrate N input | 0.010 | 0.993 |
| Soil pH * Substrate P input | Soil pH | 20.540 | < 0.001 |
|  | Substrate P input | 22.583 | < 0.001 |
|  | Soil pH:Substrate P input | 23.582 | < 0.001 |
| Soil pH * Substrate C input | Soil pH | 0.456 | 0.500 |
|  | Substrate C input | 0.043 | 0.835 |
|  | Soil pH:Substrate C input | 0.026 | 0.873 |
| Soil pH * Substrate C/N ratio | Soil pH | 0.306 | 0.580 |
|  | Substrate C/N ratio | 0.001 | 0.993 |
|  | Soil pH:Substrate C/N ratio | 0.005 | 0.943 |
| Soil pH * Substrate N/P ratio | Soil pH | 11.462 | 0.001 |
|  | Substrate N/P ratio | 12.373 | 0.001 |
|  | Soil pH:Substrate N/P ratio | 11.029 | 0.001 |
| Soil pH * Substrate C/P ratio | Soil pH | 0.323 | 0.570 |
|  | Substrate C/P ratio | 0.098 | 0.754 |
|  | Soil pH:Substrate C/P ratio | 0.085 | 0.770 |
| Soil C/N ratio * Substrate N content | Soil C/N ratio | 0.024 | 0.878 |
|  | Substrate N content | 0.001 | 0.995 |
|  | Soil C/N ratio:Substrate N content | 0.001 | 0.972 |
| Soil C/N ratio * Substrate P content | Soil C/N ratio | 5.220 | 0.022 |
|  | Substrate P content | 7.314 | 0.007 |
|  | Soil C/N ratio:Substrate P content | 6.346 | 0.012 |
| Soil C/N ratio * Substrate C content | Soil C/N ratio | 1.151 | 0.283 |
|  | Substrate C content | 0.847 | 0.357 |
|  | Soil C/N ratio:Substrate C content | 0.964 | 0.326 |
| Soil C/N ratio * Substrate N input | Soil C/N ratio | 0.650 | 0.420 |
|  | Substrate N input | 0.004 | 0.953 |
|  | Soil C/N ratio:Substrate N input | 0.029 | 0.866 |
| Soil C/N ratio * Substrate P input | Soil C/N ratio | 1.296 | 0.255 |
|  | Substrate P input | 0.033 | 0.856 |
|  | Soil C/N ratio:Substrate P input | 0.097 | 0.756 |
| Soil C/N ratio * Substrate C input | Soil C/N ratio | 0.413 | 0.521 |
|  | Substrate C input | 0.005 | 0.945 |
|  | Soil C/N ratio:Substrate C input | 0.015 | 0.902 |
| Soil C/N ratio * Substrate C/N ratio | Soil C/N ratio | 0.141 | 0.708 |
|  | Substrate C/N ratio | 0.089 | 0.766 |
|  | Soil C/N ratio:Substrate C/N ratio | 0.026 | 0.873 |
| Soil C/N ratio * Substrate N/P ratio | Soil C/N ratio | 0.048 | 0.827 |
|  | Substrate N/P ratio | 1.156 | 0.212 |
|  | Soil C/N ratio:Substrate N/P ratio | 1.156 | 0.282 |
| Soil C/N ratio * Substrate C/P ratio | Soil C/N ratio | 0.766 | 0.382 |
|  | Substrate C/P ratio | 0.127 | 0.722 |
|  | Soil C/N ratio:Substrate C/P ratio | 0.136 | 0.712 |

**Supplementary Table 7.** Multilevel meta-analysis of soil biota abundance responses to moderator interactions (climate factors × soil properties, climate factors × substrate characteristics, and soil properties × substrate characteristics) under animal-based carbon inputs. Q_m_, test statistic for individual moderator effects derived from multilevel meta-analysis models. Abbreviations: MAT, mean annual temperature; MAP, mean annual precipitation; fine texture, percentage of clay plus silt.

| **Model** | **Main and interaction effect** | **Q_m_** | ***P* value** |
| --- | --- | --- | --- |
| **Climate factors × Soil properties** | | | |
| MAT * Fine texture | MAT | 3.174 | 0.075 |
|  | Fine texture | 1.324 | 0.250 |
|  | MAT:Fine texture | 1.85 | 0.174 |
| MAT * Soil organic C | MAT | 8.617 | 0.003 |
|  | Soil organic C | 3.684 | 0.055 |
|  | MAT: Soil organic C | 6.406 | 0.011 |
| MAT * Soil total N | MAT | 3.831 | 0.050 |
|  | Soil total N | 0.534 | 0.465 |
|  | MAT:Soil total N | 1.480 | 0.224 |
| MAT * Soil pH | MAT | 1.243 | 0.265 |
|  | Soil pH | 0.115 | 0.735 |
|  | MAT:Soil pH | 0.530 | 0.467 |
| MAT * Soil C/N ratio | MAT | 0.471 | 0.492 |
|  | Soil C/N ratio | 0.005 | 0.945 |
|  | MAT:Soil C/N ratio | 0.064 | 0.800 |
| MAP * Fine texture | MAP | 5.169 | 0.023 |
|  | Fine texture | 1.562 | 0.211 |
|  | MAP:Fine texture | 2.458 | 0.117 |
| MAP * Soil organic C | MAP | 14.158 | < 0.001 |
|  | Soil organic C | 1.922 | 0.166 |
|  | MAP: Soil organic C | 4.061 | 0.044 |
| MAP * Soil total N | MAP | 14.443 | < 0.001 |
|  | Soil total N | 3.264 | 0.071 |
|  | MAP:Soil total N | 5.462 | 0.019 |
| MAP * Soil pH | MAP | 6.074 | 0.014 |
|  | Soil pH | 2.545 | 0.111 |
|  | MAP:Soil pH | 4.060 | 0.044 |
| MAP * Soil C/N ratio | MAP | 4.823 | 0.028 |
|  | Soil C/N ratio | 0.067 | 0.796 |
|  | MAP:Soil C/N ratio | 1.288 | 0.257 |
| **Climate factors × Substrate characteristics** | | | |
| MAT * Substrate N content | MAT | 0.096 | 0.757 |
|  | Substrate N content | 0.316 | 0.574 |
|  | MAT:Substrate N content | 0.017 | 0.898 |
| MAT * Substrate P content | MAT | 0.370 | 0.543 |
|  | Substrate P content | 0.770 | 0.380 |
|  | MAT:Substrate P content | 1.637 | 0.201 |
| MAT * Substrate C content | MAT | 0.015 | 0.901 |
|  | Substrate C content | 1.078 | 0.299 |
|  | MAT:Substrate C content | 0.875 | 0.350 |
| MAT * Substrate N input | MAT | 0.810 | 0.368 |
|  | Substrate N input | 0.539 | 0.463 |
|  | MAT:Substrate N input | 0.516 | 0.473 |
| MAT * Substrate P input | MAT | 1.441 | 0.230 |
|  | Substrate P input | 0.067 | 0.795 |
|  | MAT:Substrate P input | 0.249 | 0.618 |
| MAT * Substrate C input | MAT | 0.062 | 0.803 |
|  | Substrate C input | 0.950 | 0.330 |
|  | MAT:Substrate C input | 1.537 | 0.215 |
| MAT * Substrate C/N ratio | MAT | 0.335 | 0.563 |
|  | Substrate C/N ratio | 2.305 | 0.129 |
|  | MAT:Substrate C/N ratio | 3.286 | 0.070 |
| MAT * Substrate N/P ratio | MAT | 6.052 | 0.014 |
|  | Substrate N/P ratio | 0.175 | 0.676 |
|  | MAT:Substrate N/P ratio | 0.005 | 0.946 |
| MAT * Substrate C/P ratio | MAT | 2.783 | 0.095 |
|  | Substrate C/P ratio | 0.167 | 0.683 |
|  | MAT:Substrate C/P ratio | 0.536 | 0.464 |
| MAP * Substrate N content | MAP | 1.117 | 0.291 |
|  | Substrate N content | 0.063 | 0.801 |
|  | MAP:Substrate N content | 0.075 | 0.785 |
| MAP * Substrate P content | MAP | 5.884 | 0.015 |
|  | Substrate P content | 0.203 | 0.653 |
|  | MAP:Substrate P content | 0.175 | 0.676 |
| MAP * Substrate C content | MAP | 4.163 | 0.041 |
|  | Substrate C content | 1.780 | 0.182 |
|  | MAP:Substrate C content | 2.745 | 0.098 |
| MAP * Substrate N input | MAP | 4.020 | 0.045 |
|  | Substrate N input | 0.028 | 0.867 |
|  | MAP:Substrate N input | 0.002 | 0.968 |
| MAP * Substrate P input | MAP | 4.373 | 0.037 |
|  | Substrate P input | 0.002 | 0.963 |
|  | MAP:Substrate P input | 0.280 | 0.596 |
| MAP * Substrate C input | MAP | 0.887 | 0.346 |
|  | Substrate C input | 2.709 | 0.100 |
|  | MAP:Substrate C input | 4.220 | 0.040 |
| MAP * Substrate C/N ratio | MAP | 0.109 | 0.741 |
|  | Substrate C/N ratio | 0.909 | 0.340 |
|  | MAP:Substrate C/N ratio | 1.598 | 0.206 |
| MAP * Substrate N/P ratio | MAP | 4.432 | 0.018 |
|  | Substrate N/P ratio | 0.139 | 0.671 |
|  | MAP:Substrate N/P ratio | 0.16 | 0.594 |
| MAP * Substrate C/P ratio | MAP | 1.311 | 0.252 |
|  | Substrate C/P ratio | 0.126 | 0.723 |
|  | MAP:Substrate C/P ratio | 0.004 | 0.953 |
| **Soil properties × Substrate characteristics** | | | |
| Fine texture * Substrate N content | Fine texture | 4.032 | 0.045 |
|  | Substrate N content | 2.739 | 0.098 |
|  | Fine texture:Substrate N content | 4.363 | 0.037 |
| Fine texture * Substrate P content | Fine texture | 1.035 | 0.309 |
|  | Substrate P content | 1.692 | 0.193 |
|  | Fine texture:Substrate P content | 1.798 | 0.180 |
| Fine texture * Substrate C content | Fine texture | 1.096 | 0.295 |
|  | Substrate C content | 0.377 | 0.539 |
|  | Fine texture:Substrate C content | 0.471 | 0.493 |
| Fine texture * Substrate N input | Fine texture | 1.348 | 0.246 |
|  | Substrate N input | 1.926 | 0.165 |
|  | Fine texture:Substrate N input | 2.244 | 0.134 |
| Fine texture * Substrate P input | Fine texture | 0.916 | 0.339 |
|  | Substrate P input | 3.617 | 0.057 |
|  | Fine texture:Substrate P input | 4.754 | 0.029 |
| Fine texture * Substrate C input | Fine texture | 0.006 | 0.937 |
|  | Substrate C input | 1.354 | 0.245 |
|  | Fine texture:Substrate C input | 1.987 | 0.159 |
| Fine texture * Substrate C/N ratio | Fine texture | 0.035 | 0.851 |
|  | Substrate C/N ratio | 0.069 | 0.793 |
|  | Fine texture:Substrate C/N ratio | 0.153 | 0.696 |
| Fine texture * Substrate N/P ratio | Fine texture | 0.387 | 0.534 |
|  | Substrate N/P ratio | 0.526 | 0.468 |
|  | Fine texture:Substrate N/P ratio | 0.515 | 0.473 |
| Fine texture * Substrate C/P ratio | Fine texture | 2.237 | 0.134 |
|  | Substrate C/P ratio | 0.045 | 0.831 |
|  | Fine texture: Substrate C/P ratio | 0.186 | 0.666 |
| Soil organic C * Substrate N content | Soil organic C | 0.058 | 0.810 |
|  | Substrate N content | 0.429 | 0.513 |
|  | Soil organic C: Substrate N content | 0.052 | 0.820 |
| Soil organic C * Substrate P content | Soil organic C | 0.201 | 0.654 |
|  | Substrate P content | 0.201 | 0.647 |
|  | Soil organic C: Substrate P content | 0.026 | 0.871 |
| Soil organic C * Substrate C content | Soil organic C | 0.037 | 0.848 |
|  | Substrate C content | 0.500 | 0.479 |
|  | Soil organic C:Substrate C content | 0.936 | 0.333 |
| Soil organic C * Substrate N input | Soil organic C | 1.708 | 0.191 |
|  | Substrate N input | 0.035 | 0.852 |
|  | Soil organic C:Substrate N input | 0.001 | 0.980 |
| Soil organic C * Substrate P input | Soil organic C | 2.806 | 0.094 |
|  | Substrate P input | 0.076 | 0.784 |
|  | Soil organic C:Substrate P input | 0.534 | 0.465 |
| Soil organic C * Substrate C input | Soil organic C | 1.645 | 0.200 |
|  | Substrate C input | 0.021 | 0.884 |
|  | Soil organic C:Substrate C input | 0.039 | 0.843 |
| Soil organic C * Substrate C/N ratio | Soil organic C | 0.935 | 0.334 |
|  | Substrate C/N ratio | 4.105 | 0.043 |
|  | Soil organic C:Substrate C/N ratio | 5.197 | 0.023 |
| Soil organic C * Substrate N/P ratio | Soil organic C | 1.961 | 0.162 |
|  | Substrate N/P ratio | 0.004 | 0.951 |
|  | Soil organic C:Substrate N/P ratio | 0.007 | 0.935 |
| Soil organic C * Substrate C/P ratio | Soil organic C | 4.901 | 0.027 |
|  | Substrate C/P ratio | 0.001 | 0.970 |
|  | Soil organic C:Substrate C/P ratio | 0.002 | 0.966 |
| Soil total N * Substrate N content | Soil total N | 0.792 | 0.374 |
|  | Substrate N content | 2.182 | 0.140 |
|  | Soil total N:Substrate N content | 0.276 | 0.599 |
| Soil total N * Substrate P content | Soil total N | 0.141 | 0.707 |
|  | Substrate P content | 2.211 | 0.137 |
|  | Soil total N:Substrate P content | 1.311 | 0.252 |
| Soil total N * Substrate C content | Soil total N | 0.142 | 0.706 |
|  | Substrate C content | 0.013 | 0.910 |
|  | Soil total N:Substrate C content | 0.104 | 0.747 |
| Soil total N * Substrate N input | Soil total N | 0.998 | 0.318 |
|  | Substrate N input | 0.163 | 0.687 |
|  | Soil total N:Substrate N input | 0.005 | 0.943 |
| Soil total N * Substrate P input | Soil total N | 0.523 | 0.470 |
|  | Substrate P input | 0.269 | 0.604 |
|  | Soil total N:Substrate P input | 0.010 | 0.920 |
| Soil total N * Substrate C input | Soil total N | 1.498 | 0.221 |
|  | Substrate C input | 0.022 | 0.883 |
|  | Soil total N:Substrate C input | 0.404 | 0.525 |
| Soil total N * Substrate C/N ratio | Soil total N | 1.141 | 0.285 |
|  | Substrate C/N ratio | 3.200 | 0.074 |
|  | Soil total N:Substrate C/N ratio | 3.572 | 0.059 |
| Soil total N * Substrate N/P ratio | Soil total N | 2.198 | 0.138 |
|  | Substrate N/P ratio | 0.249 | 0.618 |
|  | Soil total N:Substrate N/P ratio | 0.193 | 0.661 |
| Soil total N * Substrate C/P ratio | Soil total N | 4.245 | 0.039 |
|  | Substrate C/P ratio | 2.477 | 0.116 |
|  | Soil total N:Substrate C/P ratio | 0.896 | 0.344 |
| Soil pH * Substrate N content | Soil pH | 1.144 | 0.285 |
|  | Substrate N content | 0.573 | 0.449 |
|  | Soil pH:Substrate N content | 0.216 | 0.642 |
| Soil pH * Substrate P content | Soil pH | 0.631 | 0.427 |
|  | Substrate P content | 0.266 | 0.606 |
|  | Soil pH:Substrate P content | 0.284 | 0.594 |
| Soil pH * Substrate C content | Soil pH | 2.119 | 0.145 |
|  | Substrate C content | 0.888 | 0.346 |
|  | Soil pH:Substrate C content | 0.543 | 0.461 |
| Soil pH * Substrate N input | Soil pH | 0.124 | 0.725 |
|  | Substrate N input | 1.494 | 0.222 |
|  | Soil pH:Substrate N input | 1.238 | 0.266 |
| Soil pH * Substrate P input | Soil pH | 0.023 | 0.879 |
|  | Substrate P input | 1.218 | 0.270 |
|  | Soil pH:Substrate P input | 0.840 | 0.359 |
| Soil pH * Substrate C input | Soil pH | 1.915 | 0.166 |
|  | Substrate C input | 4.389 | 0.036 |
|  | Soil pH:Substrate C input | 3.789 | 0.050 |
| Soil pH * Substrate C/N ratio | Soil pH | 0.063 | 0.801 |
|  | Substrate C/N ratio | 0.286 | 0.593 |
|  | Soil pH:Substrate C/N ratio | 0.145 | 0.704 |
| Soil pH * Substrate N/P ratio | Soil pH | 2.730 | 0.099 |
|  | Substrate N/P ratio | 0.161 | 0.688 |
|  | Soil pH:Substrate N/P ratio | 0.172 | 0.679 |
| Soil pH * Substrate C/P ratio | Soil pH | 2.078 | 0.149 |
|  | Substrate C/P ratio | 0.976 | 0.323 |
|  | Soil pH:Substrate C/P ratio | 0.509 | 0.476 |
| Soil C/N ratio * Substrate N content | Soil C/N ratio | 0.013 | 0.908 |
|  | Substrate N content | 0.017 | 0.898 |
|  | Soil C/N ratio:Substrate N content | 0.031 | 0.860 |
| Soil C/N ratio * Substrate P content | Soil C/N ratio | 0.606 | 0.436 |
|  | Substrate P content | 0.071 | 0.790 |
|  | Soil C/N ratio:Substrate P content | 0.088 | 0.767 |
| Soil C/N ratio * Substrate C content | Soil C/N ratio | 0.360 | 0.549 |
|  | Substrate C content | 0.489 | 0.484 |
|  | Soil C/N ratio:Substrate C content | 0.219 | 0.640 |
| Soil C/N ratio * Substrate N input | Soil C/N ratio | 11.02 | 0.001 |
|  | Substrate N input | 2.996 | 0.083 |
|  | Soil C/N ratio:Substrate N input | 3.428 | 0.064 |
| Soil C/N ratio * Substrate P input | Soil C/N ratio | 4.473 | 0.034 |
|  | Substrate P input | 0.108 | 0.743 |
|  | Soil C/N ratio:Substrate P input | 0.215 | 0.643 |
| Soil C/N ratio * Substrate C input | Soil C/N ratio | 0.377 | 0.539 |
|  | Substrate C input | 3.348 | 0.067 |
|  | Soil C/N ratio:Substrate C input | 2.465 | 0.116 |
| Soil C/N ratio * Substrate C/N ratio | Soil C/N ratio | 2.163 | 0.141 |
|  | Substrate C/N ratio | 4.503 | 0.034 |
|  | Soil C/N ratio:Substrate C/N ratio | 3.589 | 0.058 |
| Soil C/N ratio * Substrate N/P ratio | Soil C/N ratio | 11.423 | 0.007 |
|  | Substrate N/P ratio | 4.293 | 0.038 |
|  | Soil C/N ratio:Substrate N/P ratio | 4.197 | 0.041 |
| Soil C/N ratio * Substrate C/P ratio | Soil C/N ratio | 0.639 | 0.424 |
|  | Substrate C/P ratio | 0.675 | 0.412 |
|  | Soil C/N ratio:Substrate C/P ratio | 0.500 | 0.480 |

**Supplementary Table 8.** Summary of moderator interaction effects on soil biota abundance under plant- and animal-based carbon inputs. Abbreviations: MAT, mean annual temperature; MAP, mean annual precipitation. **P* < 0.05, ***P* < 0.01 and ****P* < 0.001.

| **Interaction** | **Factor 1** | **Factor 2** | **ESs** | **Changes %** |
| --- | --- | --- | --- | --- |
| **Plant-based carbon inputs** | | | | |
| Climate factors  ×  Substrate characteristics | MAP ≤ 1,035 mm | Substrate P input ≤ 40 kg ha^-1^ yr^-1^ | 0.229** | 25.68 |
|  | MAP ≤ 1,035 mm | Substrate P input > 40 kg ha^-1^ yr^-1^ | 1.783* | 494.89 |
|  | MAP > 1,035 mm | Substrate P input ≤ 40 kg ha^-1^ yr^-1^ | 0.679** | 97.27 |
|  | MAP > 1,035 mm | Substrate P input > 40 kg ha^-1^ yr^-1^ | 0.218 | 24.30 |
|  | MAP ≤ 1,043 mm | Substrate N/P ratio ≤ 7.6 | 1.783* | 494.89 |
|  | MAP ≤ 1,043 mm | Substrate N/P ratio > 7.6 | 0.229** | 25.73 |
|  | MAP > 1,043 mm | Substrate N/P ratio ≤ 7.6 | 0.266** | 30.42 |
|  | MAP > 1,043 mm | Substrate N/P ratio > 7.6 | 0.764** | 114.58 |
|  | MAP ≤ 1,066 mm | Substrate C/P ratio ≤ 98 | 1.783* | 494.89 |
|  | MAP ≤ 1,066 mm | Substrate C/P ratio > 98 | 0.176 | 19.23 |
|  | MAP > 1,066 mm | Substrate C/P ratio ≤ 98 | 0.518*** | 67.82 |
|  | MAP > 1,066 mm | Substrate C/P ratio > 98 | 0.708** | 103.07 |
| Soil properties  ×  Substrate characteristics | Fine texture ≤ 70% | Substrate C/P ratio ≤ 180 | 1.311** | 271.10 |
|  | Fine texture ≤ 70% | Substrate C/P ratio > 180 | 0.176 | 19.23 |
|  | Fine texture > 70% | Substrate C/P ratio ≤ 180 | 0.237** | 26.77 |
|  | Fine texture > 70% | Substrate C/P ratio > 180 | 0.571** | 76.97 |
| **Animal-based carbon inputs** | | | | |
| Climate factors  ×  Soil properties | MAP ≤ 972 mm | Soil organic C ≤ 29 g kg^-1^ | 0.520* | 68.25 |
|  | MAP > 972 mm | Soil organic C ≤ 29 g kg^-1^ | 0.582*** | 79.03 |
|  | MAP > 972 mm | Soil organic C > 29 g kg^-1^ | 0.256* | 29.20 |
|  | MAP ≤ 1,132 mm | Soil pH ≤ 7 | 0.341*** | 40.58 |
|  | MAP ≤ 1,132 mm | Soil pH > 7 | 0.508* | 66.13 |
|  | MAP > 1,132 mm | Soil pH ≤ 7 | 0.671*** | 95.54 |
|  | MAP > 1,132 mm | Soil pH > 7 | 0.062*** | 6.42 |
| Climate factors  ×  Substrate characteristics | MAP ≤ 1,148 mm | Substrate C input ≤ 0.6 t ha^-1^ yr^-1^ | 0.173 | 18.89 |
|  | MAP ≤ 1,148 mm | Substrate C input > 0.6 t ha^-1^ yr^-1^ | 0.375*** | 45.50 |
|  | MAP > 1,148 mm | Substrate C input ≤ 0.6 t ha^-1^ yr^-1^ | 0.226*** | 25.36 |
|  | MAP > 1,148 mm | Substrate C input > 0.6 t ha^-1^ yr^-1^ | 0.680*** | 97.39 |
| Soil properties  ×  Substrate characteristics | Soil organic C ≤ 18 g kg^-1^ | Substrate C/N ratio ≤ 12.5 | 0.442*** | 55.61 |
|  | Soil organic C ≤ 18 g kg^-1^ | Substrate C/N ratio > 12.5 | 0.523*** | 68.64 |
|  | Soil organic C > 18 g kg^-1^ | Substrate C/N ratio ≤ 12.5 | 0.337*** | 40.07 |
|  | Soil organic C > 18 g kg^-1^ | Substrate C/N ratio > 12.5 | 0.221 | 24.74 |
|  | Soil pH ≤ 5.6 | Substrate C input ≤ 0.9 t ha^-1^ yr^-1^ | 0.146 | 15.67 |
|  | Soil pH ≤ 5.6 | Substrate C input > 0.9 t ha^-1^ yr^-1^ | 0.601*** | 82.39 |
|  | Soil pH > 5.6 | Substrate C input ≤ 0.9 t ha^-1^ yr^-1^ | 1.195** | 230.42 |
|  | Soil pH > 5.6 | Substrate C input > 0.9 t ha^-1^ yr^-1^ | 0.366*** | 44.22 |

**Supplementary Table 9.** Comparison of random-effects and mixed-effects models for soil biota abundance under plant-based carbon inputs. Q_m_, moderator heterogeneity. LRT, likelihood ratio test. Abbreviations: MAT, mean annual temperature; MAP, mean annual precipitation; fine texture, percentage of clay plus silt.

| **Model ID** | **Fixed term (s)** | **df** | **AIC** | **Moderators** | | **Comparison** | **LRT** | |
| --- | --- | --- | --- | --- | --- | --- | --- | --- |
|  |  |  |  | **Q_m_** | ***P* value** |  | **Statistic** | ***P* value** |
| M_1_ | *β*_0_ | 403 | 731.660 |  |  | - |  |  |
| M_2_ | *β*_0_ + MAT | 402 | 733.649 | 0.012 | 0.914 | M_2_ vs. M_1_ | 0.012 | 0.914 |
| M_3_ | *β*_0_ + MAT + MAT^2^ | 401 | 734.581 | 1.083 | 0.582 | M_3_ vs. M_1_ | 1.080 | 0.583 |
|  |  |  |  |  |  | M_3_ vs. M_2_ | 1.068 | 0.301 |
| M_4_ | *β*_0_ | 403 | 734.534 |  |  | - |  |  |
| M_5_ | *β*_0_ + MAP | 402 | 736.504 | 0.030 | 0.862 | M_5_ vs. M_4_ | 0.030 | 0.863 |
| M_6_ | *β*_0_ + MAP + MAP^2^ | 401 | 727.708 | 11.15 | 0.004 | M_6_ vs. M_4_ | 10.826 | 0.004 |
|  |  |  |  |  |  | M_6_ vs. M_5_ | 10.796 | 0.001 |
| M_7_ | *β*_0_ | 403 | 731.802 |  |  | - |  |  |
| M_8_ | β_0_ + Fine texture | 402 | 724.166 | 10.88 | 0.001 | M_8_ vs. M_7_ | 9.636 | 0.002 |
| M_9_ | β_0_ + Fine texture + Fine texture^2^ | 401 | 724.989 | 12.43 | 0.002 | M_9_ vs. M_7_ | 10.813 | 0.004 |
|  |  |  |  |  |  | M_9_ vs. M_8_ | 1.177 | 0.278 |
| M_10_ | *β*_0_ | 403 | 724.402 |  |  | - |  |  |
| M_11_ | *β*_0_ + Soil organic C | 402 | 726.393 | 0.010 | 0.921 | M_11_ vs. M_10_ | 0.010 | 0.921 |
| M_12_ | *β*_0_ + Soil organic C + Soil organic C^2^ | 401 | 728.390 | 0.012 | 0.994 | M_12_ vs. M_10_ | 0.012 | 0.994 |
|  |  |  |  |  |  | M_12_ vs. M_11_ | 0.002 | 0.963 |
| M_13_ | *β*_0_ | 403 | 734.259 |  |  | - |  |  |
| M_14_ | *β*_0_ + Soil total N | 402 | 736.229 | 0.030 | 0.862 | M_14_ vs. M_13_ | 0.030 | 0.862 |
| M_15_ | *β*_0_ + Soil total N +Soil total N^2^ | 401 | 737.300 | 0.970 | 0.616 | M_15_ vs. M_13_ | 0.959 | 0.619 |
|  |  |  |  |  |  | M_15_ vs. M_14_ | 0.929 | 0.335 |
| M_16_ | *β*_0_ | 403 | 734.415 |  |  | - |  |  |
| M_17_ | *β*_0_ + Soil C/N ratio | 402 | 736.403 | 0.012 | 0.912 | M_17_ vs. M_16_ | 0.012 | 0.912 |
| M_18_ | *β*_0_ + Soil C/N ratio +Soil C/N ratio^2^ | 401 | 738.154 | 0.263 | 0.877 | M_18_ vs. M_16_ | 0.261 | 0.618 |
|  |  |  |  |  |  | M_18_ vs. M_17_ | 0.249 | 0.618 |
| M_19_ | *β*_0_ | 403 | 729.323 |  |  | - |  |  |
| M_20_ | *β*_0_ + Soil pH | 402 | 729.995 | 1.373 | 0.241 | M_20_ vs. M_19_ | 1.329 | 0.249 |
| M_21_ | *β*_0_ + Soil pH + Soil pH^2^ | 401 | 731.993 | 1.377 | 0.502 | M_21_ vs. M_19_ | 1.330 | 0.514 |
|  |  |  |  |  |  | M_21_ vs. M_20_ | 0.002 | 0.966 |
| M_22_ | *β*_0_ | 124 | 202.925 |  |  | - |  |  |
| M_23_ | *β*_0_ + Substrate N content | 123 | 204.888 | 0.040 | 0.842 | M_23_ vs. M_22_ | 0.037 | 0.847 |
| M_24_ | *β*_0_ + Substrate N content + Substrate N content^2^ | 122 | 205.924 | 1.006 | 0.605 | M_24_ vs. M_22_ | 1.001 | 0.606 |
|  |  |  |  |  |  | M_24_ vs. M_23_ | 0.964 | 0.326 |
| M_25_ | *β*_0_ | 81 | 138.453 |  |  | - |  |  |
| M_26_ | *β*_0_ + Substrate P content | 80 | 139.019 | 1.607 | 0.205 | M_26_ vs. M_25_ | 1.434 | 0.231 |
| M_27_ | *β*_0_ + Substrate P content +Substrate P content^2^ | 79 | 138.566 | 4.821 | 0.090 | M_27_ vs. M_25_ | 3.887 | 0.143 |
|  |  |  |  |  |  | M_27_ vs. M_26_ | 2.453 | 0.117 |
| M_28_ | *β*_0_ | 116 | 197.549 |  |  | - |  |  |
| M_29_ | *β*_0_ + Substrate C content | 115 | 199.371 | 0.196 | 0.658 | M_29_ vs. M_28_ | 0.179 | 0.673 |
| M_30_ | *β*_0_ + Substrate C content +Substrate C content^2^ | 114 | 200.930 | 0.626 | 0.731 | M_30_ vs. M_28_ | 0.620 | 0.734 |
|  |  |  |  |  |  | M_30_ vs. M_29_ | 0.441 | 0.507 |
| M_31_ | *β*_0_ | 113 | 204.412 |  |  | - |  |  |
| M_32_ | *β*_0_ + Substrate N input | 112 | 206.105 | 0.312 | 0.577 | M_32_ vs. M_31_ | 0.307 | 0.579 |
| M_33_ | *β*_0_ + Substrate N input +Substrate N input^2^ | 111 | 206.623 | 1.810 | 0.405 | M_33_ vs. M_31_ | 1.788 | 0.409 |
|  |  |  |  |  |  | M_33_ vs. M_32_ | 1.482 | 0.224 |
| M_34_ | *β*_0_ | 71 | 138.646 |  |  | - |  |  |
| M_35_ | *β*_0_ + Substrate P input | 70 | 140.609 | 0.039 | 0.843 | M_35_ vs. M_34_ | 0.037 | 0.847 |
| M_36_ | *β*_0_ + Substrate P input +Substrate P input^2^ | 69 | 136.740 | 10.30 | 0.006 | M_36_ vs. M_34_ | 5.906 | 0.052 |
|  |  |  |  |  |  | M_36_ vs. M_35_ | 5.869 | 0.015 |
| M_37_ | *β*_0_ | 110 | 207.234 |  |  | - |  |  |
| M_38_ | *β*_0_ + Substrate C input | 109 | 209.151 | 0.084 | 0.773 | M_38_ vs. M_37_ | 0.084 | 0.773 |
| M_39_ | *β*_0_ + Substrate C input +Substrate C input^2^ | 108 | 208.462 | 2.866 | 0.239 | M_39_ vs. M_37_ | 2.772 | 0.250 |
|  |  |  |  |  |  | M_39_ vs. M_38_ | 2.689 | 0.101 |
| M_40_ | *β*_0_ | 120 | 214.888 |  |  | - |  |  |
| M_41_ | *β*_0_ + Substrate C/N ratio | 119 | 216.602 | 0.302 | 0.583 | M_41_ vs. M_40_ | 0.286 | 0.593 |
| M_42_ | *β*_0_ + Substrate C/N ratio + Substrate C/N ratio^2^ | 118 | 218.480 | 0.413 | 0.813 | M_42_ vs. M_40_ | 0.408 | 0.816 |
|  |  |  |  |  |  | M_42_ vs. M_41_ | 0.121 | 0.728 |
| M_43_ | *β*_0_ | 83 | 152.988 |  |  | - |  |  |
| M_44_ | *β*_0_ + Substrate N/P ratio | 82 | 153.131 | 1.877 | 0.171 | M_44_ vs. M_43_ | 1.858 | 0.173 |
| M_45_ | *β*_0_ + Substrate N/P ratio + Substrate N/P ratio^2^ | 81 | 154.146 | 2.958 | 0.228 | M_45_ vs. M_43_ | 2.843 | 0.241 |
|  |  |  |  |  |  | M_45_ vs. M_44_ | 0.985 | 0.321 |
| M_46_ | *β*_0_ | 72 | 141.195 |  |  | - |  |  |
| M_47_ | *β*_0_ + Substrate C/P ratio | 71 | 143.117 | 0.079 | 0.778 | M_47_ vs. M_46_ | 0.077 | 0.781 |
| M_48_ | *β*_0_ + Substrate C/P ratio + Substrate C/P ratio^2^ | 70 | 142.941 | 12.44 | 0.002 | M_48_ vs. M_46_ | 2.254 | 0.324 |
|  |  |  |  |  |  | M_48_ vs. M_47_ | 2.176 | 0.140 |

**Supplementary Table 10.** Comparison of random-effects and mixed-effects models for soil biota abundance under animal-based carbon inputs. Q_m_, moderator heterogeneity. LRT, likelihood ratio test. Abbreviations: MAT, mean annual temperature; MAP, mean annual precipitation; fine texture, percentage of clay plus silt.

| **Model ID** | **Fixed term (s)** | **df** | **AIC** | **Moderators** | | **Comparison** | **LRT** | |
| --- | --- | --- | --- | --- | --- | --- | --- | --- |
|  |  |  |  | **Q_m_** | ***P* value** |  | **Statistic** | ***P* value** |
| M_1_ | *β*_0_ | 191 | 367.868 |  |  | - |  |  |
| M_2_ | *β*_0_ + MAT | 190 | 365.803 | 5.639 | 0.018 | M_2_ vs. M_1_ | 4.065 | 0.044 |
| M_3_ | *β*_0_ + MAT + MAT^2^ | 189 | 367.667 | 5.949 | 0.051 | M_3_ vs. M_1_ | 4.201 | 0.122 |
|  |  |  |  |  |  | M_3_ vs. M_2_ | 0.136 | 0.712 |
| M_4_ | *β*_0_ | 191 | 359.462 |  |  | - |  |  |
| M_5_ | *β*_0_ + MAP | 190 | 356.011 | 7.391 | 0.007 | M_5_ vs. M_4_ | 5.452 | 0.020 |
| M_6_ | *β*_0_ + MAP + MAP^2^ | 189 | 357.316 | 8.203 | 0.017 | M_6_ vs. M_4_ | 2.147 | 0.046 |
|  |  |  |  |  |  | M_6_ vs. M_5_ | 0.695 | 0.405 |
| M_7_ | *β*_0_ | 191 | 364.772 |  |  | - |  |  |
| M_8_ | β_0_ + Fine texture | 190 | 364.985 | 1.822 | 0.177 | M_8_ vs. M_7_ | 1.787 | 0.181 |
| M_9_ | β_0_ + Fine texture + Fine texture^2^ | 189 | 359.888 | 12.780 | 0.002 | M_9_ vs. M_7_ | 8.884 | 0.012 |
|  |  |  |  |  |  | M_9_ vs. M_8_ | 7.097 | 0.008 |
| M_10_ | *β*_0_ | 191 | 369.224 |  |  | - |  |  |
| M_11_ | *β*_0_ + Soil organic C | 190 | 365.693 | 6.777 | 0.009 | M_11_ vs. M_10_ | 5.532 | 0.019 |
| M_12_ | *β*_0_ + Soil organic C + Soil organic C^2^ | 189 | 356.750 | 33.528 | < 0.001 | M_12_ vs. M_10_ | 16.474 | < 0.001 |
|  |  |  |  |  |  | M_12_ vs. M_11_ | 10.942 | 0.001 |
| M_13_ | *β*_0_ | 191 | 369.968 |  |  | - |  |  |
| M_14_ | *β*_0_ + Soil total N | 190 | 368.125 | 4.632 | 0.031 | M_14_ vs. M_13_ | 3.843 | 0.050 |
| M_15_ | *β*_0_ + Soil total N +Soil total N^2^ | 189 | 365.369 | 11.625 | 0.003 | M_15_ vs. M_13_ | 8.599 | 0.014 |
|  |  |  |  |  |  | M_15_ vs. M_14_ | 4.756 | 0.029 |
| M_16_ | *β*_0_ | 191 | 363.841 |  |  | - |  |  |
| M_17_ | *β*_0_ + Soil C/N ratio | 190 | 365.193 | 0.737 | 0.391 | M_17_ vs. M_16_ | 0.648 | 0.421 |
| M_18_ | *β*_0_ + Soil C/N ratio +Soil C/N ratio^2^ | 189 | 366.314 | 1.632 | 0.442 | M_18_ vs. M_16_ | 1.527 | 0.466 |
|  |  |  |  |  |  | M_18_ vs. M_17_ | 0.878 | 0.349 |
| M_19_ | *β*_0_ | 191 | 372.030 |  |  | - |  |  |
| M_20_ | *β*_0_ + Soil pH | 190 | 369.337 | 5.239 | 0.022 | M_20_ vs. M_19_ | 4.693 | 0.030 |
| M_21_ | *β*_0_ + Soil pH + Soil pH^2^ | 189 | 371.297 | 5.226 | 0.073 | M_21_ vs. M_19_ | 4.372 | 0.094 |
|  |  |  |  |  |  | M_21_ vs. M_20_ | 0.040 | 0.842 |
| M_22_ | *β*_0_ | 184 | 347.987 |  |  | - |  |  |
| M_23_ | *β*_0_ + Substrate N content | 183 | 345.778 | 7.832 | 0.005 | M_23_ vs. M_22_ | 4.209 | 0.040 |
| M_24_ | *β*_0_ + Substrate N content +Substrate N content^2^ | 182 | 345.299 | 14.586 | 0.001 | M_24_ vs. M_22_ | 6.688 | 0.035 |
|  |  |  |  |  |  | M_24_ vs. M_23_ | 2.478 | 0.115 |
| M_25_ | *β*_0_ | 178 | 346.308 |  |  | - |  |  |
| M_26_ | *β*_0_ + Substrate P content | 177 | 348.238 | 0.076 | 0.783 | M_26_ vs. M_25_ | 0.070 | 0.791 |
| M_27_ | *β*_0_ + Substrate P content +Substrate P content^2^ | 176 | 349.993 | 0.320 | 0.852 | M_27_ vs. M_25_ | 0.315 | 0.855 |
|  |  |  |  |  |  | M_27_ vs. M_26_ | 0.245 | 0.621 |
| M_28_ | *β*_0_ | 144 | 276.410 |  |  | - |  |  |
| M_29_ | *β*_0_ + Substrate C content | 143 | 276.344 | 2.203 | 0.138 | M_29_ vs. M_28_ | 2.066 | 0.151 |
| M_30_ | *β*_0_ + Substrate C content +Substrate C content^2^ | 142 | 278.309 | 2.250 | 0.325 | M_30_ vs. M_28_ | 2.102 | 0.350 |
|  |  |  |  |  |  | M_30_ vs. M_29_ | 0.035 | 0.851 |
| M_31_ | *β*_0_ | 175 | 340.379 |  |  | - |  |  |
| M_32_ | *β*_0_ + Substrate N input | 174 | 341.593 | 0.792 | 0.373 | M_32_ vs. M_31_ | 0.787 | 0.375 |
| M_33_ | *β*_0_ + Substrate N input +Substrate N input^2^ | 173 | 343.282 | 1.101 | 0.577 | M_33_ vs. M_31_ | 1.098 | 0.578 |
|  |  |  |  |  |  | M_33_ vs. M_32_ | 0.311 | 0.578 |
| M_34_ | *β*_0_ | 169 | 337.043 |  |  | - |  |  |
| M_35_ | *β*_0_ + Substrate P input | 168 | 338.649 | 0.580 | 0.446 | M_35_ vs. M_34_ | 0.394 | 0.530 |
| M_36_ | *β*_0_ + Substrate P input +Substrate P input^2^ | 167 | 340.629 | 0.575 | 0.750 | M_36_ vs. M_34_ | 0.414 | 0.813 |
|  |  |  |  |  |  | M_36_ vs. M_35_ | 0.020 | 0.888 |
| M_37_ | *β*_0_ | 139 | 270.118 |  |  | - |  |  |
| M_38_ | *β*_0_ + Substrate C input | 138 | 271.078 | 1.096 | 0.295 | M_38_ vs. M_37_ | 1.040 | 0.308 |
| M_39_ | *β*_0_ + Substrate C input +Substrate C input^2^ | 137 | 272.771 | 1.597 | 0.450 | M_39_ vs. M_37_ | 1.347 | 0.510 |
|  |  |  |  |  |  | M_39_ vs. M_38_ | 0.307 | 0.580 |
| M_40_ | *β*_0_ | 144 | 275.095 |  |  | - |  |  |
| M_41_ | *β*_0_ + Substrate C/N ratio | 143 | 276.808 | 0.325 | 0.569 | M_41_ vs. M_40_ | 0.287 | 0.592 |
| M_42_ | *β*_0_ + Substrate C/N ratio + Substrate C/N ratio^2^ | 142 | 272.176 | 12.012 | 0.003 | M_42_ vs. M_40_ | 6.919 | 0.031 |
|  |  |  |  |  |  | M_42_ vs. M_41_ | 6.632 | 0.010 |
| M_43_ | *β*_0_ | 178 | 348.131 |  |  | - |  |  |
| M_44_ | *β*_0_ + Substrate N/P ratio | 177 | 350.129 | 0.002 | 0.966 | M_44_ vs. M_43_ | 0.002 | 0.967 |
| M_45_ | *β*_0_ + Substrate N/P ratio + Substrate N/P ratio^2^ | 176 | 351.290 | 1.031 | 0.597 | M_45_ vs. M_43_ | 0.841 | 0.657 |
|  |  |  |  |  |  | M_45_ vs. M_44_ | 0.839 | 0.360 |
| M_46_ | *β*_0_ | 138 | 258.060 |  |  | - |  |  |
| M_47_ | *β*_0_ + Substrate C/P ratio | 137 | 259.401 | 0.999 | 0.318 | M_47_ vs. M_46_ | 0.659 | 0.417 |
| M_48_ | *β*_0_ + Substrate C/P ratio + Substrate C/P ratio^2^ | 136 | 259.541 | 4.232 | 0.121 | M_48_ vs. M_46_ | 2.519 | 0.284 |
|  |  |  |  |  |  | M_48_ vs. M_47_ | 1.860 | 0.173 |

**Supplementary Table 11.** Heterogeneity tests (Q statistics) for soil biota abundance under plant- and animal-based carbon inputs. ****P* < 0.001.

| **Function** | **Plant-based carbon inputs** | | |  | **Animal-based carbon inputs** | | |
| --- | --- | --- | --- | --- | --- | --- | --- |
|  | **df** | **Q** | ***P* value** |  | **df** | **Q** | ***P* value** |
| Soil biota abundance | 403 | 778774 | < 0.001*** |  | 191 | 98443 | < 0.001*** |

**Supplementary References**

1. Assessment ME. *Ecosystems and Human Well-being: Synthesis*: Island press, 2005.

2. Carpenter SR, Mooney HA, Agard J *et al.* Science for managing ecosystem services: beyond the millennium ecosystem assessment. *Proc Natl Acad Sci USA* 2009; **106**(5): 1305-1312.

3. Gibson L, Lee TM, Koh LP *et al.* Primary forests are irreplaceable for sustaining tropical biodiversity. *Nature* 2011; **478**(7369): 378-381.

4. Hedges LV, Gurevitch J, Curtis PS. The meta-analysis of response ratios in experimental ecology. *Ecology* 1999; **80**(4): 1150-1156.

5. Rossetti MR, Tscharntke T, Aguilar R *et al.* Responses of insect herbivores and herbivory to habitat fragmentation: a hierarchical meta-analysis. *Ecol Lett* 2017; **20**(2): 264-272.

6. Tamburini G, Bommarco R, Wanger TC *et al.* Agricultural diversification promotes multiple ecosystem services without compromising yield. *Sci Adv* 2020; **6**(45): eaba1715.

7. Benitez-Lopez A, Alkemade R, Schipper AM *et al.* The impact of hunting on tropical mammal and bird populations. *Science* 2017; **356**(6334): 180-183.

8. Viechtbauer W. Conducting Meta-Analyses in R with the metafor Package. *J Stat Softw* 2010; **36**(3): 1-48.

9. Feng Y, Schmid B, Loreau M *et al.* Multispecies forest plantations outyield monocultures across a broad range of conditions. *Science* 2022; **376**(6595): 865-868.

10. Hedges LV, Olkin I. *Statistical methods for meta-analysis*: Academic press, 2014.

11. Seufert V, Ramankutty N, Foley JA. Comparing the yields of organic and conventional agriculture. *Nature* 2012; **485**(7397): 229-232.

12. Spake R, Mori AS, Beckmann M *et al.* Implications of scale dependence for cross-study syntheses of biodiversity differences. *Ecol Lett* 2021; **24**(2): 374-390.

13. Olkin I, Gleser L. Stochastically dependent effect sizes. In: Cooper H, Hedges LV, Valentine JC (eds.). *The Handbook Of Research Synthesis Meta-analysis*: Russell Sage Foundation; 2009. 357-376.

14. Lajeunesse MJ. On the meta-analysis of response ratios for studies with correlated and multi-group designs. *Ecology* 2011; **92**(11): 2049-2055.

15. Lajeunesse MJ, Fitzjohn R. Facilitating systematic reviews, data extraction and meta‐analysis with the metagear package for R. *Methods Ecol Evol* 2015; **7**(3): 323-330.

16. Midolo G, De Frenne P, Holzel N *et al.* Global patterns of intraspecific leaf trait responses to elevation. *Glob Chang Biol* 2019; **25**(7): 2485-2498.

17. Zhang P, Li B, Wu J *et al.* Invasive plants differentially affect soil biota through litter and rhizosphere pathways: a meta-analysis. *Ecol Lett* 2019; **22**(1): 200-210.

18. Jennions MD, Moller AP. Publication bias in ecology and evolution: an empirical assessment using the 'trim and fill' method. *Bio Rev* 2002; **77**(2): 211-222.

19. Rosenberg MS. The File-Drawer Problem Revisited: A General Weighted Method for Calculating Fail-Safe Numbers in Meta-Analysis. *Evolution* 2005; **59**(2): 464-468.

20. Egger M, Davey Smith G, Schneider M *et al.* Bias in meta-analysis detected by a simple, graphical test. *BMJ* 1997; **315**(7109): 629-634.

21. Zhao SX, Schmidt S, Gao HJ *et al.* A precision compost strategy aligning composts and application methods with target crops and growth environments can increase global food production. *Nat Food* 2022; **3**(9): 741-752.

22. Breheny P, Burchett W. Visualization of regression models using visreg. *The R Journal* 2017; **9**(2): 56-71.

23. Vendig I, Guzman A, De La Cerda G *et al.* Quantifying direct yield benefits of soil carbon increases from cover cropping. *Nat Sustain* 2023; **6**(9): 1125-1134.

24. Wittwer RA, Bender SF, Hartman K *et al.* Organic and conservation agriculture promote ecosystem multifunctionality. *Sci Adv* 2021; **7**(34): eabg6995.
